# Rescue of Glycosylphosphatidylinositol-Anchored Protein Biosynthesis Using Synthetic Glycosylphosphatidylinositol Oligosaccharides

**DOI:** 10.1021/acschembio.1c00465

**Published:** 2021-10-07

**Authors:** Paula A. Guerrero, Yoshiko Murakami, Ankita Malik, Peter H. Seeberger, Taroh Kinoshita, Daniel Varón Silva

**Affiliations:** † Department of Biomolecular Systems, Max Planck Institute of Colloids and Interfaces, Am Muehlenberg 1, 14424 Potsdam, Germany; ‡ Department of Chemistry and Biochemistry, Freie Universität Berlin, Arnimallee 22, 14195 Berlin, Germany; § Yabumoto Department of Intractable Disease Research, Research Institute for Microbial Diseases, Osaka University, 3-1 Yamada-Oka, Osaka 565-0871, Japan; ∥ Laboratory of Immunoglycobiology, WPI Immunology Frontier Research Center, Osaka University, 3-1 Yamada-Oka, Osaka 565-0871, Japan

## Abstract

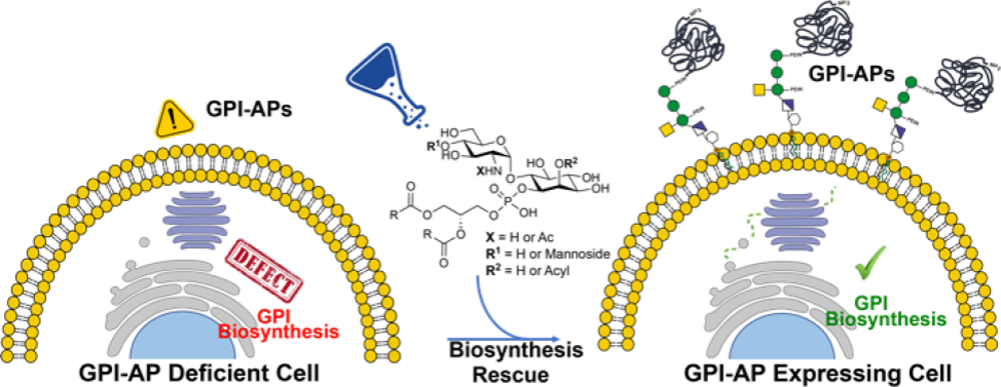

The attachment of proteins to the cell membrane using a glycosylphosphatidylinositol (GPI) anchor is a ubiquitous process in eukaryotic cells. Deficiencies in the biosynthesis of GPIs and the concomitant production of GPI-anchored proteins lead to a series of rare and complicated disorders associated with inherited GPI deficiencies (IGDs) in humans. Currently, there is no treatment for patients suffering from IGDs. Here, we report the design, synthesis, and use of GPI fragments to rescue the biosynthesis of GPI-anchored proteins (GPI-APs) caused by mutation in genes involved in the assembly of GPI-glycolipids in cells. We demonstrated that the synthetic fragments GlcNAc-PI (**1**), Man-GlcN-PI (**5**), and GlcN-PI with two (**3**) and three lipid chains (**4**) rescue the deletion of the GPI biosynthesis in cells devoid of the PIGA, PIGL, and PIGW genes in vitro. The compounds allowed for concentration-dependent recovery of GPI biosynthesis and were highly active on the cytoplasmic face of the endoplasmic reticulum membrane. These synthetic molecules are leads for the development of treatments for IGDs and tools to study GPI-AP biosynthesis.

## Introduction

**Figure 1:**
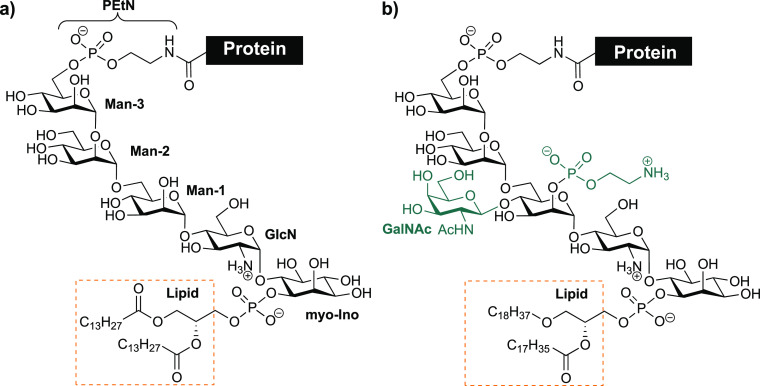
GPI Structure. (a) Core structure found in all GPIs. (b) Common structure found in human GPIs. Man = mannose, GlcN = glucosamine, *myo*-Ino = *myo*-Inositol, GalNAc = *N*-acetylgalactosamine, and PEtN = phosphoethanolamine.

Glycosylphosphatidylinositols (GPIs) are complex glycolipids attaching many eukaryotic proteins to the cell membrane.^1^ GPIs are added as a post-translational modification to the C-terminus of proteins that contain a signal peptide sequence directing GPI attachment.^2^ Around 150 human proteins use GPI for anchoring to the cell membrane forming GPI-anchored proteins (GPI-APs).^3^ The proteins include hydrolytic enzymes, adhesion molecules, protease inhibitors, receptors, and regulatory proteins of the immune system.^4^ The structure of all GPIs has a conserved core structure having the pseudopentasaccharide glycan core Man-α-(1 → 2)-Man-α-(1 → 6)-Man-α-(1 → 4)-GlcN-α-(1 → 6)-myo-Ino, a phosphoethanolamine unit (PEtN), and a phospholipid (Figure 1a).^1^ This structure can be modified in a cell- and tissue-dependent manner with additional glycans and fatty acid chains and by phosphorylation.^5^ Two additional units are common in mammalian GPIs: a PEtN at the 2-O position and a GalNAc branch at the 4-O position on the Man-1 (Figure 1b).^6^ Further modifications of mammalian GPI glycans include a Man-4 residue that is attached to the 2-O position of Man-3 and the addition of galactose and *N*-acetylneuraminic acid to the GalNAc branch,^7^ among others.

The lipid part of GPI-APs is highly variable and can contain saturated and unsaturated alkyl chains of different lengths as part of a diacylglycerol, an alkylacylglycerol (AAG), or a ceramide.^8^ In mammalian cells, the most common lipids are 1-alkyl-2-acyl-sn-glycerol and 1,2-diacyl-sn-glycerol having an sn-1 saturated C18 or C16 alkyl chain and a C18:0 at the sn-2 position.^9,10^ However, a small fraction of GPIs containing unsaturated alkyl chains have been identified as well.^10^

**Figure 2:**
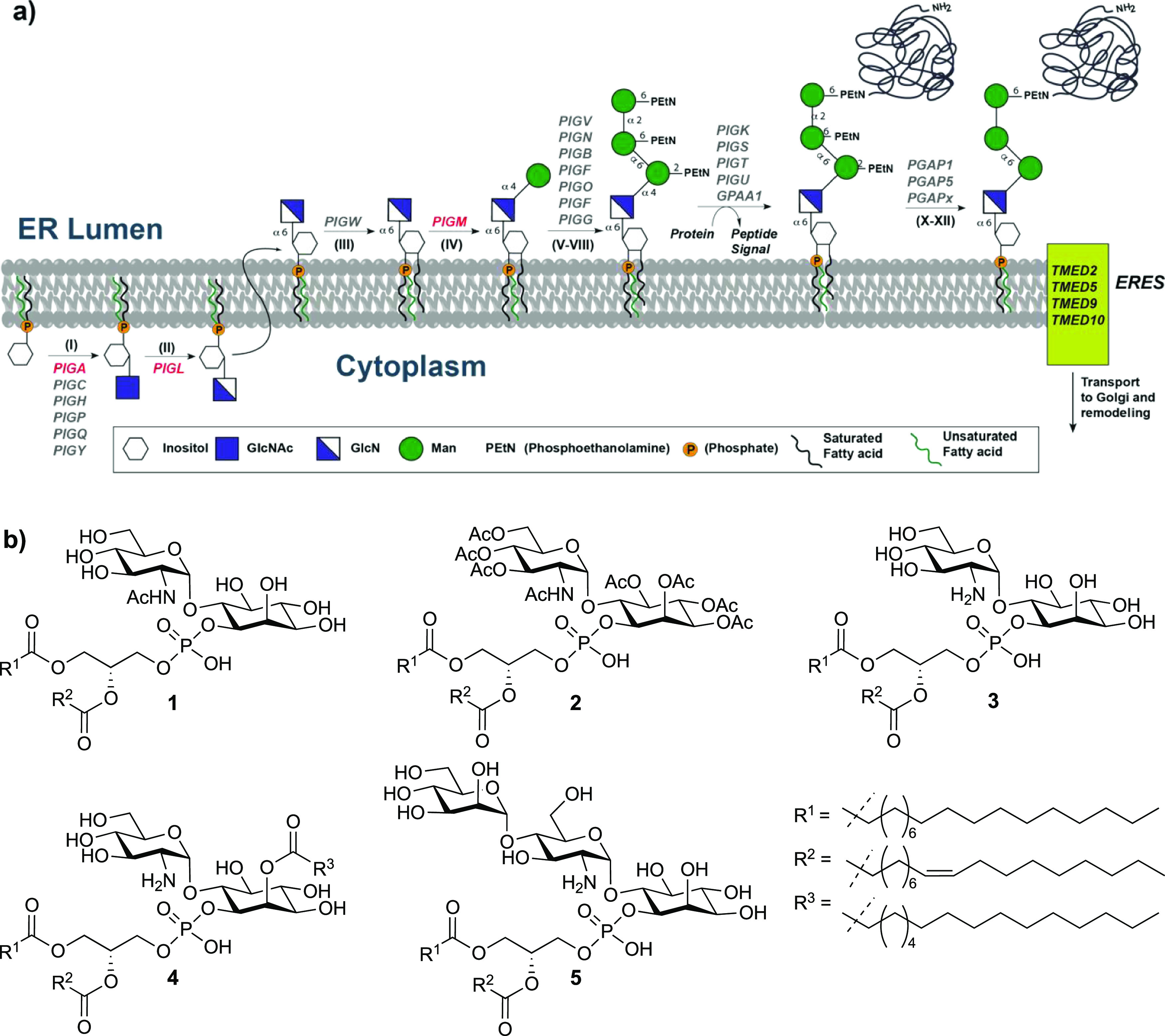
Biosynthesis of GPI-APs. (a) Schematic representation of GPI-anchored protein biosynthesis. (b) Designed GPI fragments (**1**–**5**) to rescue GPI biosynthesis.

The biosynthesis of GPIs involves the assembly of the GPI glycolipid, the transfer to the protein, and a glycan and lipid remodeling.^11,12^ The process starts on the cytoplasmic side of the (endoplasmic reticulum) ER membrane with the transfer of *N*-acetylglucosamine (GlcNAc) to phosphatidylinositol (PI) forming GlcNAc-PI (step I, Figure 2a).^13,14^ This *N*-acetylglucosamine is deacetylated in the next step, and the product flips into the ER lumen. In the ER lumen, a third acyl chain is added, and the glycan is elongated with mannoses and the additions of PEtN units up to obtain the GPI anchor.^9,15^ The fully assembled glycolipid is transferred to proteins containing the corresponding peptide signal by action of the GPI–transamidase complex.^9,12^ Upon attachment of the GPI to the protein, the GPI–glycan and lipid are modified in the ER and then transported to the Golgi bodies for further modification.^16,17^ Little is known about GPI–glycan remodeling, but it may depend on the glycosylation machinery of the cell type and the protein.

At least 27 genes participate in the biosynthesis of GPI-APs. Among them, 22 genes are phosphatidylinositolglycan genes (PIG) involved in the GPI-core preassembly and following attachment to the protein (Figure 2a). The other five genes participate in the modification of GPIs and are called post GPI attachment to protein genes.^15^ The variability observed in GPIs results from a series of enzymatic modifications taking place in the Golgi after the GPI-anchored proteins are released from the ER. Defects in any of the 21 genes involved in GPI biosynthesis can induce a decreased or deleted expression of GPI-APs on the cell surface leading to inherited GPI deficiency disorders (IGDs).^11,15^

The majority of IGD patients with GPI biosynthesis deficiencies in the first two steps of the process, attachment of GlcNAc to PI and de-N-acetylation to GlcN-PI, present developmental delay, intellectual disability, and frequent epilepsy.^18^ In addition, hypotonia, spasticity, rigidity, dystonia, ataxia, reflex anomalies, tremors, and choreiform movements have been reported. Somatic mutation of PIGA leads to paroxysmal nocturnal hemoglobinuria (PNH),^19^ a disorder involving red blood cell destruction due to a lack of CD55 and CD59, two GPI-anchored proteins that regulate the complement system.^20^ Other anomalies and clinical features are widespread and correspond to the deleted expression of PIGA in various tissues, including the brain, liver, heart, and blood cells.^19^ Patients with PIGL gene mutations suffer from developmental delay, seizures, dysmorphic features, and cranial shape anomalies. A feature that was not observed in patients with mutations in other PIG genes is ophthalmologic involvement, in particular formation of colobomas.^21^

Treatment with pyridoxine (dephosphorylated vitamin B6) is effective to control seizures in some IGD patients.^22^ However, treatment options for GPI-AP biosynthesis deficiencies are limited and focus mostly on treating PNH caused by somatic mutations of the PIGA gene. PNH treatments include the use of monoclonal antibodies to regulate the activity of the complement system,^23,24^ immunosuppressive therapy to reduce the activity of the immune system and protect the bone marrow,^25^ and bone marrow transplantation, the only potential cure for this disorder.^26^ Efficient therapies for IGD patients are direly needed.

GPIs are difficult to isolate from natural sources to investigate their biological activity. Several groups have established synthetic strategies to obtain these molecules and GPI fragments with different modifications.^27−32^ A major challenge in GPI synthesis is the incorporation of unsaturated fatty acids that is not compatible with the reductive conditions used to cleave benzyl ethers and esters that are commonly used as permanent protecting groups. Thus, methods using 4-methoxybenzyl ether and benzoyl ester protection have been evaluated for this purpose.^28,33^ We developed a strategy using 2-naphthylmethyl (Nap) ether group for permanent protection during the synthesis of GPI-derivatives containing unsaturated lipids.^34^ The Nap ethers are stable and can be cleaved by treatment with acid (TFA) or under oxidative conditions using 2,3-dichloro-5,6-dicyano-1,4-benzoquinone (DDQ).

Here, we designed a series of synthetic glycolipids containing an unsaturated fatty lipid chain and evaluated their activity for in vitro rescue of GPI biosynthesis. The compounds contain the pseudotrisaccharide (Man-GlcN-*myo*-Ino), the pseudodisaccharide GlcNAc-*myo*-Ino or GlcN-*myo*-Ino glycan fragments, and a diacylglycerol containing an oleic acid chain at the *sn*-2 position, which resemble the products of the four initial steps of GPI biosynthesis. We established the activity of these compounds as substrates to rescue the biosynthesis of GPI-APs in HEK293 cells having knockout the PIGA, PIGL, PIGW, and PIGM genes. We determined the concentration dependency and cross responses of the compounds and showed that synthetic GPI fragments can rescue the expression of GPI-APs. Synthetic GPI can serve as a starting point to develop glycan-based treatments of IGDs derived from depletion of enzymes involved in the GPI biosynthesis.

## Results and Discussion

### Glycan Design

Glycolipids **1**–**5** resemble the products and substrates of the enzymes involved in the four initial steps of GPI biosynthesis (Figure 2b). Compounds **1** (GlcNAc-PI) and **2** (peracetylated GlcNAc-PI) were designed to resemble the product of the first step of the biosynthesis, the transfer of GlcNAc to PI. The pseudodisaccharide **3** (GlcN-PI) was designed to cover the product of the deacetylation of GlcNAc-PI. The trilipidated glycolipid **4** (GlcN-acylPI) resembles the product of the following step after the flip of the GPI precursor into the lumen of the ER and contains a palmitate (C16:0) at the C-2 position of *myo*-inositol. Pseudotrisaccharide **5** (Man-GlcN-PI) was synthesized to determine the activity in the following step, the transfer of the first mannose. We selected the naturally occurring 1-stearoyl(C18:0)-2-oleoyl(C18:1)-sn-glycero-phosphate as a lipid moiety containing an unsaturated chain at the sn-2 position to represent the lipid of the endogenous PI that contains predominantly 1-stearoyl-2-arachidonoyl(C20:4)-sn-glycerolipid.^35^ The PI is the starting building block for the GPI biosynthesis that is modified after acylation of *myo*-inositol in GlcN-PI and again following the assembly of the entire GPI and the transfer to the proteins.^16,36^

### Synthesis of GPI Fragments

**Scheme 1:**
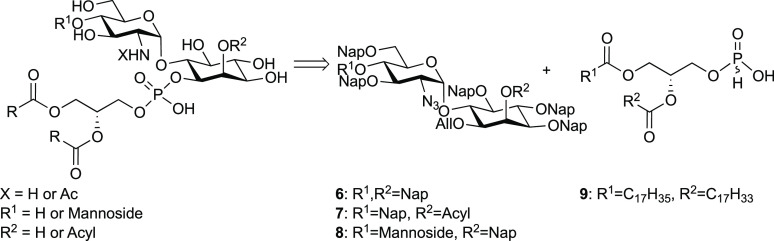
Retrosynthetic Analysis of GPI Fragments **1–5** (Nap = 2-Naphthylmethyl)

The strategy for the synthesis of the compounds involved the assembly of the glycan using a mannose, a glucosamine and a *myo*-inositol building block, and late-stage installation of the lipid by phosphitylation of the glycans with the corresponding H-phosphonate **9** (Scheme 1). The assembly of the glycan relied on Nap protected inositol to synthesize the pseudodisaccharide **10**(34) and elongation with the mannosyl imidate **23**(37) to obtain the trisaccharide **8** (Scheme 1).

GPI fragment assembly commenced with the deacetylation of pseudodisaccharide **10**(34) and the protection of the triol using 2-(naphthyl)methyl bromide and NaH to give the fully protected pseudodisaccharide **6**. Removal of the allyl group from the pseudodisaccharide **6** with palladium chloride provided the pseudodisaccharide alcohol **11** ready for phospholipid installation. The lipid was installed following a two-step protocol of phosphitylation with the H-phosphonate **9** using pivaloyl chloride activation and oxidation with iodine and water to deliver the phospholipidated protected pseudodisaccharide **12**. Next, the 2-naphthylmethyl groups of **12** were removed under oxidative conditions using DDQ before the remaining azide was reduced with trimethylphosphine in THF, to furnish GPI fragment **3** in pure form in 47% yield over two steps.

**Scheme 2:**
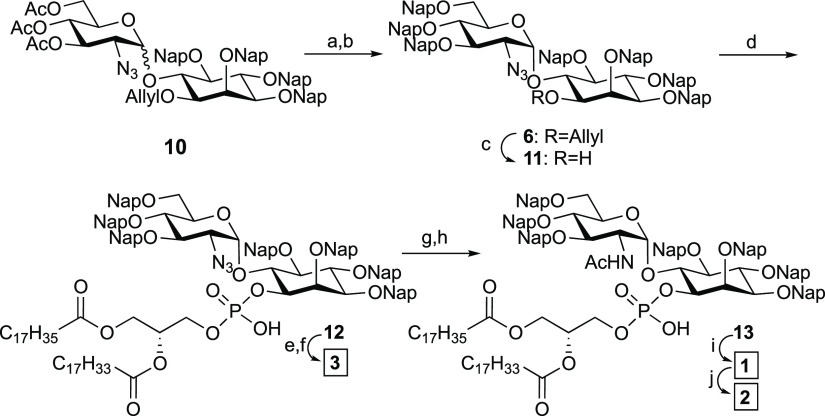
Synthesis of the GPI Fragments **1–3** Reagents and conditions: (a) NaOMe, MeOH, 40 °C, 94% (two steps); (b) NapBr, NaH, DMF, r.t., 92%; (c) PdCl_2_, DCM/MeOH, r.t., 85%; (d) **9**, (i) Piv-Cl, pyridine, r.t.; (ii) I_2_, H_2_O, r.t., 98%; (e) DDQ, MeOH/DCM, r.t.; (f) P(CH_3_)_3_, THF, r.t., 47% (two steps); (g) P(CH_3_)_3_, THF, r.t.; (h) Ac_2_O, pyridine, r.t.; (i) DDQ, MeOH/DCM, r.t., 62% (three steps); (j) Ac_2_O, pyridine. Nap = 2-naphthylmethyl, Piv-Cl = pivaloyl chloride.

GPI fragments **1** and **2** were prepared from **12** (Scheme 2). The azide in **12** was reduced with trimethylphosphine in THF, and the obtained amine was acetylated using acetic anhydride and pyridine to give the fully protected pseudodisaccharide. Removal of the 2-naphthylmethyl ethers of **13** by treatment with DDQ in a MeOH/DCM mixture provided the glycolipid **1** in 62% yield over three steps. Finally, the N-acetylated pseudodisaccharide **1** was peracetylated by treatment with acetic anhydride and pyridine to obtain the fully acetylated fragment **2**.

**Scheme 3:**
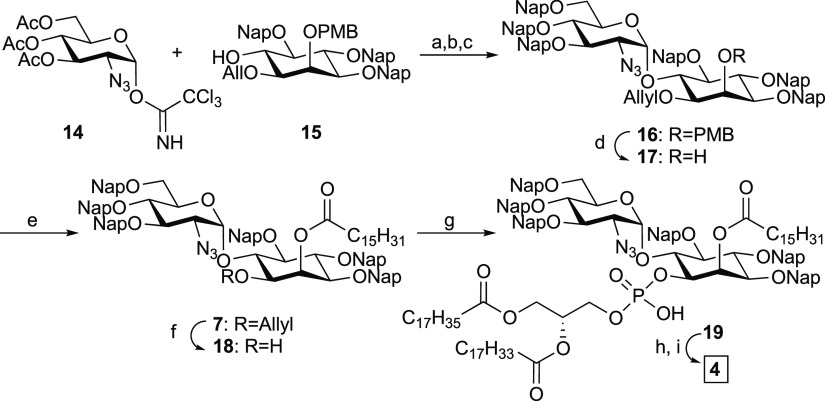
Synthesis of GPI Fragment **4** Reagents and conditions: (a) TMSOTf, Et_2_O/DCM, 0 °C, 7:1 (α/β); (b) NaOMe, MeOH, 40 °C, 77% (two steps); (c) NapBr, NaH, DMF, r.t., 79%; (d) TFA, DCM, 0 °C, 97%; (e) palmitic acid, DIC, DMAP, DCM, r.t., 86%; (f) PdCl_2_, DCM/MeOH, r.t., 85%; g) (i) **9**, Piv-Cl, pyridine, r.t.; (ii) I_2_, H_2_O, r.t., 82%; (h) DDQ, MeOH/DCM, r.t.; (i) P(CH_3_)_3_, THF, r.t., 48% (two steps). PMB = 4-methoxybenzyl, Nap = 2-naphthylmethyl, TFA = trifluoroacetic acid, DIC = *N*,*N*′-diisopropylcarbodiimide, DMAP = 4-dimethylaminopyridine, and Piv-Cl = pivaloyl chloride.

The synthesis of trilipidated pseudodisaccharide **4** required the synthesis of a modified pseudodisaccharide having an orthogonal group at the 2-O position of inositol.^38^ The process started with the glycosylation of inositol **15**(38) with glycosyl imidate **14** by TMSOTf activation to obtain a pseudodisaccharide. Following deacetylation with sodium methoxide in methanol and etherification with 2-naphthylmethyl bromide delivered the protected pseudodisaccharide **16** in 61% yield over the three steps. The 4-methoxybenzyl group on inositol was selectively removed under acidic conditions, and the resulting alcohol was acylated with the palmitic acid by DIC/DMAP activation to give **7**. The allyl group was removed using palladium chloride, and the phospholipid was installed using H-phosphonate **9**. Finally, the 2-naphthylmethyl groups were removed with DDQ, and the remaining azide was reduced using trimethylphosphine to give trilipidated fragment **4** in 48% yield (Scheme 3).

**Scheme 4:**
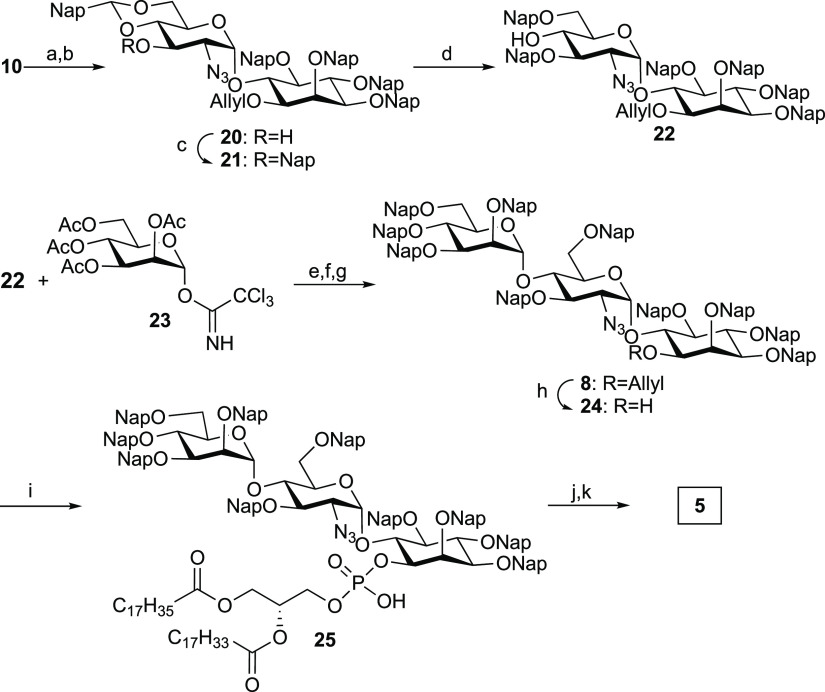
Synthesis of GPI Fragment **5** Reagents and conditions: (a) NaOMe, MeOH, 40 °C, 94% (two steps); (b) CSA, 2-(dimethoxymethyl)naphthalene, ACN, r.t., 91%; (c) TBAI, NapBr, NaH, DMF, r.t., 98%; (d) Me_2_EtSiH, Cu(OTf)_2_, ACN/DCM, 0 °C, 74%; (e) TMSOTf, Et_2_O/DCM, 0 °C, 84%; (f) NaOMe, MeOH, r.t.; (g) NapBr, NaH, DMF, r.t., 75% (two steps); (h) PdCl_2_, DCM/MeOH, r.t., 83%; (i) (i) **9**, Piv-Cl, pyridine, r.t.; (ii) I_2_, H_2_O, r.t., 93%; (j) DDQ, MeOH/DCM, r.t.; (k) P(CH_3_)_3_, THF, r.t., 56% (two steps). Nap = 2-naphthylmethyl, CSA = camphorsulfonic acid, TBAI = tetra-n-butylammonium iodide.

The synthesis of pseudotrisaccharide **5** commenced with the deacetylation of pseudodisaccharide **10** and treatment under acidic conditions with freshly prepared 2-(dimethoxymethyl)naphthalene to give the 4,6-cyclic acetal **20** (Scheme 4). The remaining free hydroxyl group of **20** was protected as a 2-naphthylmethyl ether to obtain the fully protected pseudodisaccharide **21**. Selective opening of the cyclic acetal in **21** using dimethylethylsilane and catalytic amounts of Cu(OTf)_2_ afforded pseudodisaccharide glycosyl acceptor **22** that was glycosylated at 0 °C with the mannosyl glycosylating agent **23** under TMSOTf activation in 84% yield. The resulting pseudotrisaccharide was deacetylated and reprotected using 2-naphthylmethyl bromide using Williamson conditions to give the fully protected pseudotrisaccharide **8**. Following deallylation to furnish **24** and phospholipidation of the alcohol using the H-phosphonate **9** and oxidation with iodine and water delivered the phosphorylated pseudotrisaccharide **25**. Finally, a two-step global deprotection involving the removal of the 2-naphthylmethyl groups with DDQ and the reduction of the azide with trimethylphosphine delivered GPI fragment **5** in good yield.

### Recovery of GPI-AP Biosynthesis in HEK293 Cells

To determine the activity of the synthetic GPI fragments in the recovery of the GPI-AP biosynthesis, HEK293 cells lacking the PIGA gene (PIGA-KO) were treated first in a serum-free DMEM/F-12 culture medium with concentrations between 1 and 50 μM of compounds **1** (GlcNAc-PI) and **2** (peracetylated GlcNAc-PI). After 24 h, the restored expression of total GPI-APs, CD59 and DAF on the cells was analyzed by flow cytometry using fluorescent-labeled inactive toxin aerolysin (FLAER), anti-CD59 and anti-DAF antibodies, respectively, for detection.

**Figure 3:**
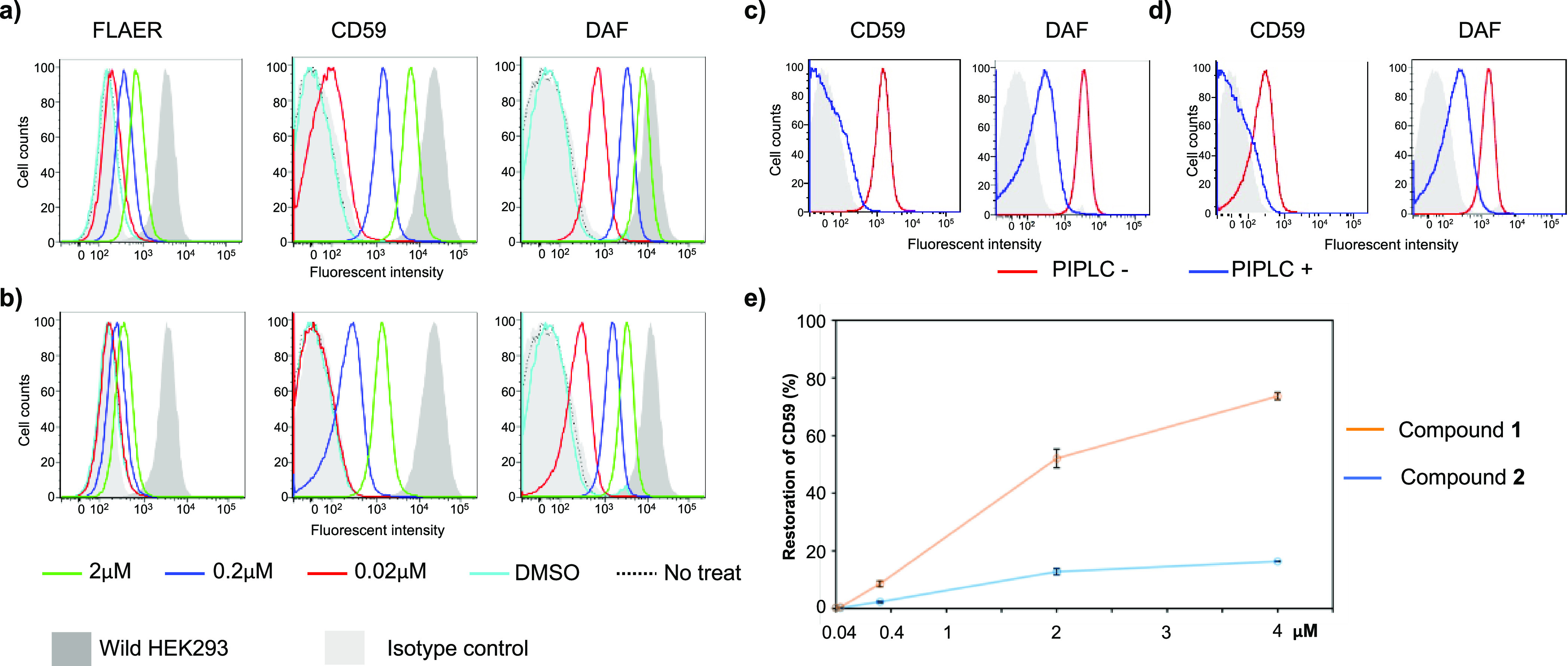
Determination of GPI-APs by flow cytometry after treatment of PIGA-KO HEK293 cells with compounds **1** (GlcNAc-PI) and **2** (peracetylated GlcNAc-PI). (a) Production of GPI-APs, as determined by FLAER, CD59, and DAF after treatment with compound **1**. (b) Production of GPI-AP FLAER, CD59, and DAF after treatment with **2**. (c) Analysis of the cells treated with **1** with and without PIPLC. (d) Analysis of the cells treated with **2** with and without PIPLC. (e) Percent of rescued expression of CD59 with various concentrations of compounds **1** and **2**. CD59 expression of the treatment with compounds **1** and **2** was the average of two independent experiments. Histograms in (a–d): *y* axis shows cell counts; the *x* axis shows fluorescence intensity.

Cells treated with compounds **1** and **2** showed the rescue of the biosynthesis pathway and the expression of GPI-APs. The expression of total GPI-APs (determined by FLAER), CD59, and DAF in PIGA-KO HEK293 was increased in a dose-dependent manner and showed 22, 30, and 70% of wild-type cell levels, respectively, by 2 μM compound **1** (GlcNAc-PI) treatment (Figure 3a). The expression of the GPI-APs in cells treated with compound **2** (peracetylated GlcNAc-PI) was also efficient but with lower intensities than those observed with GlcNAc-PI (Figure 3b). Peracetylation of the compound might have facilitated transmembrane incorporation of compound **2** into the cells; however, a removal of the acetyl groups by cellular esterases is needed before the compound can enter the GPI biosynthesis. The latter might have decreased a rescue efficiency by compound **2**. Thus, we consider compound **1** an ideal candidate for future in vivo studies and a reference to compare the activity of the other compounds to rescue GPI biosynthesis.

To demonstrate the presence of GPI anchoring for the proteins, the expression of the CD59 and DAF proteins was analyzed after treating the cells with PI-specific phospholipase C (PIPLC), an enzyme that cleaves at the PI releasing the GPI-APs from the membrane. The expression of CD59 and DAF was sensitive to treatment with the PIPLC showing nearly complete removal of CD59 and a partial removal of DAF from the membrane, which confirmed that they were expressed as GPI-APs (Figure 3c,d). Compounds **1** and **2** recovered the surface expression of CD59 in a dose-dependent manner (Figure 3e).

**Figure 4:**
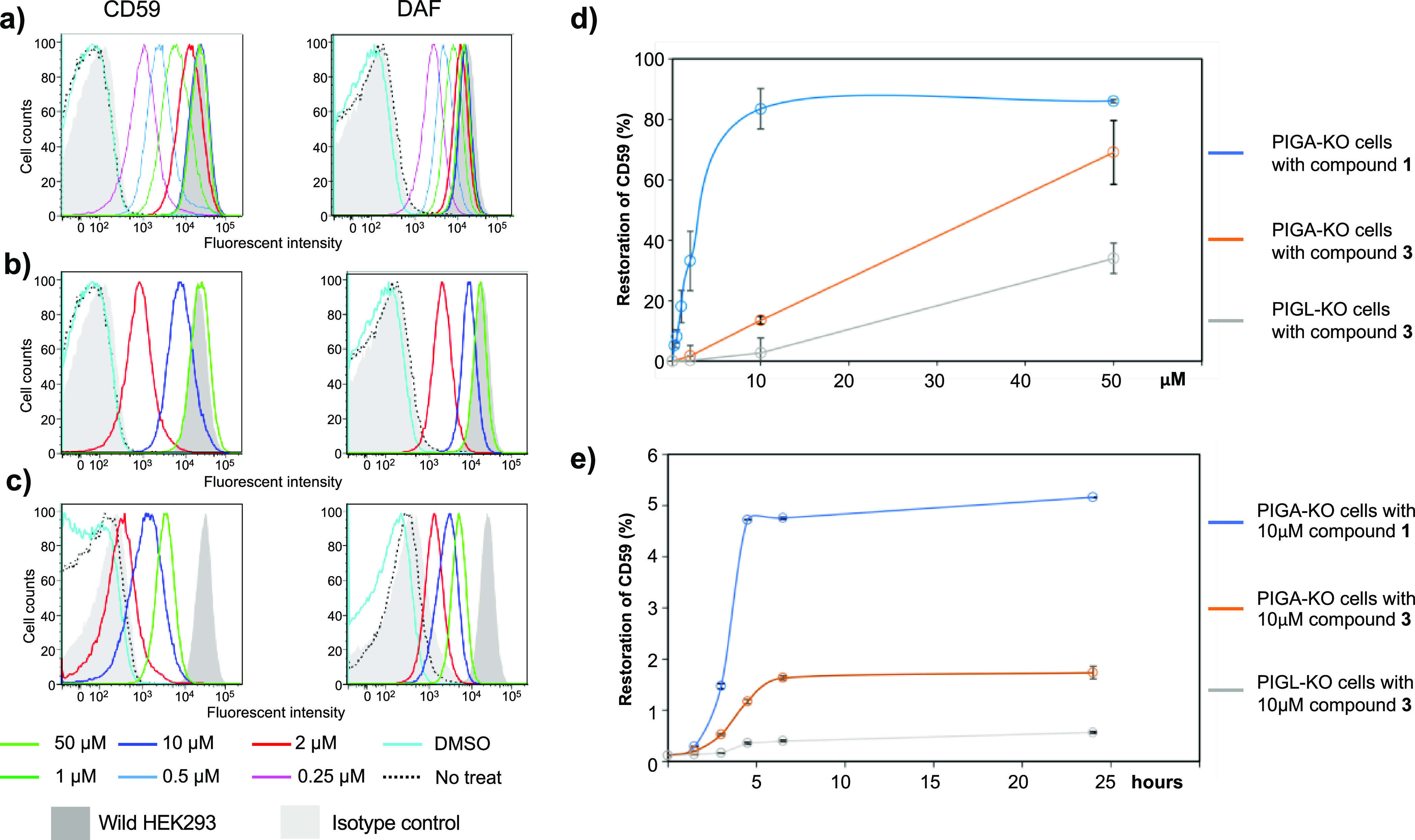
Determination of GPI-APs by flow cytometry after treatment of PIGA-KO and PIGL-KO HEK293 cells with **3** (GlcN-PI) in comparison with **1** (GlcNAc-PI). (a) Restoration of CD59 and DAF expression on PIGA-KO cells after 24 h incubation with compound **1** at various concentrations. (b) Restoration of CD59 and DAF expression on PIGA-KO cells after 24 h incubation with **3** at various concentrations. (c) Restoration of CD59 and DAF expression on PIGL-KO cells after 24 h incubation with compound **3** at various concentrations. (d) Percent CD59 restoration of wild-type cells at various concentrations of compounds **1** and **3** calculated from the fluorescence intensity in histograms (a–c). (e) Percent restorations of CD59 expression were plotted at various time points. Histograms in (a–c): *y* axis shows cell counts; the *x* axis shows fluorescence intensity. The values plotted in charts (d) and (e) are the result of duplicate experiments.

Next, we analyzed the activity of compound **3** (GlcN-PI) to restore the expression of GPI-APs in PIGA-KO HEK293 cells and compared it to compound **1** (GlcNAc-PI). The activity of compound **3** was also compared in PIGA-KO cells and in PIGL-KO cells. FACS analysis showed the activity of compound **1** in various concentrations from 0.25 to 50 μM to restore the expression of GPI-anchored CD59 and DAF in PIGA-KO cells (Figure 4a). Compound **3** restored the surface expression of GPI-APs in PIGA-KO cells, however less efficiently than compound **1** (Figure 4b). Compound **3** was less efficient in PIGL-KO cells than in PIGA-KO cells (Figure 4c). These data are graphically presented, showing that the restoration of wild-type CD59 levels correlates with compound concentrations (Figure 4d).

Pulse-chase analysis using compounds **1** and **3** in PIGA-KO cells and PIGL-KO cells was performed to further compare their efficiencies. After pulse incubation with 10 μM compounds for 1 h at 4 ° C, the cells were washed and then cultured at 37 °C for various time periods to chase restoration of CD59. Percent restoration of wild-type CD59 levels was plotted as a function of time (Figure 4e). While restored levels of CD59 were different, both compounds showed similar kinetics of CD59 restoration in which the cell surface CD59 started to appear after only 1.5 h chase and plateaued at 5–6 h of chase. It is not clear what factors reduced the activity of compound **3** compared to **1**. Thus, further studies are necessary to evaluate the effects of the zwitterionic character of **3** and the introduction of modifications to improve the incorporation of this pseudodisaccharide to the ER membrane.

**Figure 5:**
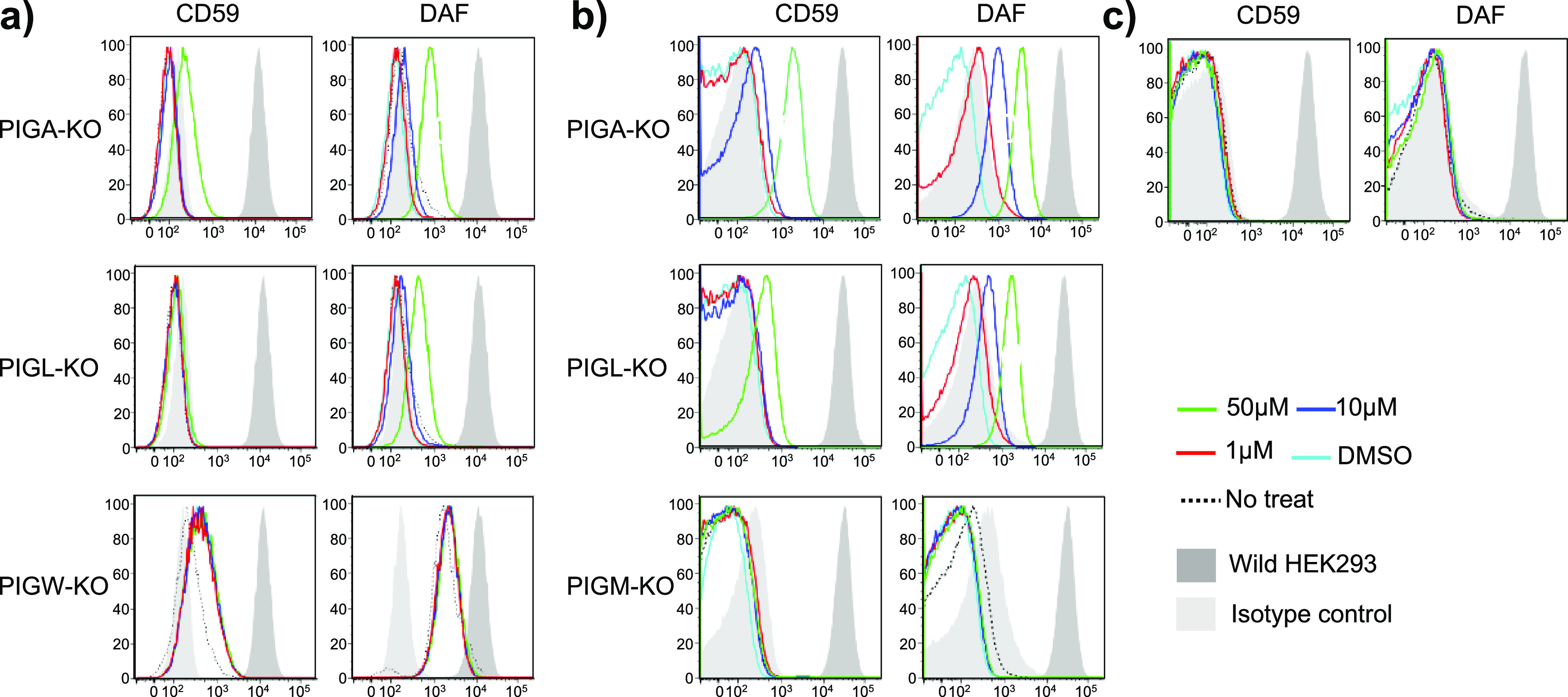
Determination of GPI-APs by flow cytometry after treatment of PIGA-, PIGL-, PIGW-, and PIGM-KO cells with compounds **4** (GlcN-acyPI) and **5** (Man-GlcN-PI). **(**a) CD59 and DAF expression on PIGA-, PIGL-, and PIGW-KO cells after 24 h incubation with compound **4**; (b) CD59 and DAF expression on PIGA-, PIGL-, and PIGM-KO cells after 24 h incubation with compound **5**; (c) CD59 and DAF expression on PIGM/PIGA DKO cells after 24 h incubation with compound **5**. Histograms in (a–c): *y* axis shows cell counts; the *x* axis shows fluorescence intensity.

We then tested compound **4** (GlcN-acyPI) for its ability to restore PIGW-KO cells. It could not restore the surface expression of GPI-APs in PIGW-KO cells at all, suggesting that trilipidated compound **4** cannot reach the luminal side of the ER (Figure 5a, bottom panels). The GPI biosynthesis in PIGW-KO cells proceeds without inositol acylation by PIGW that leads to a partial and low-level expression of GPI-APs (Figure 5a). Based on this finding, the activity of pseudotrisaccharide **5** (Man-GlcN-PI) was examined since we expected that this dilipidated compound might reach the ER lumen. However, compound **5** was unable to restore the expression of GPI-APs in PIGM-KO cells (Figure 5b, bottom panels). To eliminate a potential effect of the accumulation of the endogenous products of GPI intermediates, which might inhibit utilization of the compound, trisaccharide **5** (Man-GlcN-PI) was employed to study rescue of GPI-AP biosynthesis in PIGM/PIGA double KO (DKO) cells instead of PIGM-KO cells. Still, no recovery of the GPI-APs by compound **5** was observed (Figure 5c), suggesting that the lack of the activity of compound **5** was due to the poor transport of this compound to the lumen of the ER.

To determine whether compounds **4** and **5** entered the cells, we evaluated the activity of these compounds in PIGA-KO and PIGL-KO cells. Compounds **4** and **5** very weakly restored GPI-APs on both PIGA-KO cells and PIGL-KO cells (Figure 5a,b) with an activity only 1/100 to 1/20 of the activity found with compounds **1** and **3**. These results suggested that entrance of fragments **4** (GlcN-acylPI) and **5** (Man-GlcN-PI) into the GPI biosynthesis requires the removal of the inositol-linked acyl chain from compound **4** or the mannose unit from compound **5** during the transport from the cell surface to the cytoplasmic side of the ER and the generation of small amounts of fragment **3** (GlcN-PI). This portion of fragment **3** forming in the cells recovered the biosynthesis, although with the low expression of GPI-APs on the cells. Finally, we performed rescue experiments with glycolipids **1**, **4**, and **5** using either streptolysin O from Hemolytic *streptococcus* or compounds formulated in liposomes to increase cell membrane permeability and compound incorporation.^39^ Only compound **1** showed activity under these conditions (Figures S1 and S2), confirming that the lack of transport into the lumen of the ER may be the limitation for getting activity of compounds **4** and **5** in PIGW- and PIGM-KO cells.

## Discussion

The complex biosynthesis of GPI-APs suggests multiple effects of the glycolipid on the activity and properties of the proteins.^11^ GPI-APs are associated with rafts,^40^ the platforms of the interaction of protein complexes on the membrane, and the modulation of the immune system in parasitic infections.^6,41^ The biosynthesis process involves enzymatic steps in the cytoplasmic and luminal sides of the ER membrane that can be affected by mutations of the genes of the corresponding enzymes. Mutations and defects in the production of GPI-APs and the deficiency of these molecules on the surface cause complicated disorders (IGDs) in humans.^11,15^

We synthesized five glycolipids (**1**–**5**) to resemble four products of GPI biosynthesis and evaluated the activity of these compounds to rescue gene knockouts for these enzymatic steps in HEK293 cells. To obtain lipid compositions similar to the natural GPI intermediates, the compounds were phospholipidated with an H-phosphonate having a diacylglycerol with a lipid composition found in human cells.^35^ The synthesis involved a convergent strategy and required 2-naphthylmethyl ethers for glycan protection and a global deprotection under oxidative conditions with DDQ to maintain lipid unsaturation. Recovery of the depletion of the transfer of *N*-acetylglucosamine and the deacetylation of GlcNAc in PIGA and PIGL-KO HEK293 cells showed high activity and a concentration-dependent activity of compounds **1 (**GlcNAc-PI), **2 (**peracetylated GlcNAc-PI), and **3** (GlcN-PI) (Figures 3 and 4). Unexpectedly, compounds **4** (GlcN-acylPI) and **5** (Man-GlcN-PI) could not rescue the steps in the ER lumen (Figure 5). The transport of the compounds to the cytoplasmic side of the ER membrane is unknown. Possible mechanisms may involve a flip to the cytoplasmic leaflet of the plasma membrane (PM), where the compounds are endocytosed or transferred through membrane contact sites between the PM and ER.^42^

The difference of activity between structures **1**–**3** and **4**–**5** correlated with the localization of the processes. The rescue was only possible for the steps taking place at the cytoplasmic side of the ER. The acylation of inositol and the first mannosylation in the ER lumen could not be rescued by compounds **4** and **5**, and this lack of activity persisted using cell membrane permeabilization with streptolysin O or formulation with liposomes. However, these compounds were also active and reestablished the production of GPI-APs in PIGA-KO cells, albeit at lower levels. The reduced rescue activity and acceptance of compounds **2**, **4**, and **5** in the process denote the need for further intracellular transformations of these compounds before their incorporation into the biosynthesis.

Compounds **4** and **5** did not rescue the biosynthesis steps in the ER lumen, suggesting either the lack of insertion into the ER membrane or the absence of the required transport or flip into the lumen of the ER. The lack of transport into the ER may depend on the absence of active transport mechanisms for the compounds or a defective passive crossing through the ER membrane by **4** and **5** due to the lower concentration of cholesterol and high concentration of unsaturated lipids in the ER membrane compared to the cell membrane.^43^

Several lines of evidence indicate that free GPIs and GPI intermediates are expressed on the cell surface of normal cells and GPI biosynthesis defective cells.^44−49^ According to our preliminary data on subcellular fractionation and lipidomic analysis, some GlcNAc-PI accumulated in PIGL-KO cells and some Man-(EtNP)Man-GlcN-acylPI accumulated in PIGB-KO cells are transported to the PM. Those intermediates might be transported back to the ER or to lysosomes for degradation. It is feasible that all synthetic compounds were inserted into the PM and were transported by these mechanisms to obtain the observed activity. These studies are in progress.

## Conclusions

We designed and synthesized a series of glycolipids corresponding to the initial products of the GPI biosynthesis in eukaryotes. We used these compounds to study the biosynthesis of GPI and showed the production of GPI-APs in PIGA and PIGL knockout HEK293 cells. The pseudodisaccharides **1** (GlcNAc-PI) and **3** (GlcN-PI) are highly active at micromolar concentration and serve as substrates to rescue the steps in the cytoplasmic side of the ER and recovery of expression of the GPI-APs. Synthetic GPI fragments **4** (GlcN-acylPI) and **5** (Man-GlcN-PI) cannot reach the ER lumen and were found to be unable to rescue GPI biosynthesis. Most likely, GPI fragments larger than **1** and **3** cannot be used by cells for GPI biosynthesis without the support transport mechanism or delivery systems bringing them to the luminal side of the ER membrane. Compounds **1** and **3** are leads for the development of treatments against IGD pathologies involving mutations in the PIGA and PIGL genes.

## Methods

### Synthesis of the Compounds

The experiments for the synthesis and characterization of the glycolipids **1**–**5** are described in the Supporting Information.

### Generation of PIGA-, PIGL-, PIGW-, and PIGM-KO HEK293 Cells

Knockout cells were generated using the CRISPR/Cas9 system. HEK293 cells were transfected with pX330-U6-Chimeric_BB-CBh-hSpCas9 containing each gRNA (AGCATTCTGCATGGCGATCG for PIGA, GACGGCTGGGAGCCGAAAGC and GCTTGGCCCGCCTAAGGCAC for PIGL, TGTTGTATCAAATATACCGA and AGAGATTATTCCTTCGCGGT for PIGW, TGTCCGTATACCTCACGTGC for PIGM). Two weeks later, the cells were stained for CD59 expression and negative cells were sorted by FACS. Several clones of each gene knockout were selected by limiting dilution and sanger sequenced to confirm mutations.

### Compound Treatment of Cells

Dried compounds were reconstituted with DMSO at a concentration of 10 mM (stock solution). The cells (1 × 10^5^/well) were incubated overnight in 12-well plates, and the medium was changed into 500 μL of serum-free medium (D-MEM/Ham‘s F-12) containing the described concentrations of compounds. The negative control contained the same percentage of DMSO as the sample with the highest compound concentration. After 24 h incubation, the cells were analyzed by FACS. For further PIPLC treatment, compound-treated cells were harvested and incubated in 50 μL of serum-free medium containing 0.05 U/mL of PIPLC (Thermo Fisher Scientific) for 1.5 h at 37 °C and were analyzed by FACS.

### Pulse-Chase Analysis of Treated Cells

PIGA-KO and PIGL-KO cells were incubated in 24-well plates with 5 × 10^4^ cells per well overnight and pulsed with 250 μL of serum-free medium containing a 10 μM concentration of compounds for 1 h at 4 °C. After washing, they were chased and analyzed by FACS. Percent restorations of CD59 expression to wild-type cells were plotted in various time points.

### Flow Cytometry Analysis

The cells were harvested and stained with anti-CD59 (5H8) or anti-DAF (IA10) monoclonal antibodies followed by second antibody, anti-mouse IgG-PE. Some cells were also stained with Alexa488 labeled inactive toxin aerolysin (FLAER) to detect total GPI-APs. Stained cells were analyzed using a flow cytometer (MACSQuant VYB, Milteny Biotec).

## Supporting Information Available

Full experimental details, characterization and spectra of the compounds, and FACS data for the studies with liposomes and streptolysin O (PDF)
