# Supplementary material for: Rescue of Glycosylphosphatidylinositol-Anchored Protein Biosynthesis Using Synthetic Glycosylphosphatidylinositol Oligosaccharides
Source: ACS Chem Biol. 2021 Oct 7;16(11):2297–306. doi: 10.1021/acschembio.1c00465 (PMC8609528; doi:10.1021/acschembio.1c00465)

# Rescue of Glycosylphosphatidylinositol-Anchored Protein Biosynthesis Using Synthetic Glycosylphosphatidylinositol Oligosaccharides

Paula A. Guerrero,[a,b,†] Yoshiko Murakami,[c,d,†] Ankita Malik,[a,b] Peter H. Seeberger,[a,b] Taroh Kinoshita[c,d] and Daniel Varón Silva[a,b*]

[a] Department of Biomolecular Systems, Max Planck Institute of Colloids and Interfaces, Am Muehlenberg 1, 14424 Potsdam, Germany

[b] Department of Chemistry and Biochemistry, Freie Universität Berlin, Arnimallee 22, 14195 Berlin, Germany

[c] Yabumoto Department of Intractable Disease Research, Research Institute for Microbial Diseases, Osaka University, 3-1 Yamada-Oka, Osaka 565-0871, Japan

[d] Laboratory of Immunoglycobiology, WPI Immunology Frontier Research Center, Osaka University, 3-1 Yamada-Oka, Osaka 565-0871, Japan

† These authors contributed equally

* Corresponding Author: Dr. Daniel Varón Silva, email: daniel.varon@mpikg.mpg.de

## SUPPORTING INFORMATION

### Table of contents

General Information ........ 1
Synthesis of Compounds 1-3 ........ 2
Synthesis of compound 4 ........ 7
Synthesis of Compound 5 ........ 13
Cell Assays ........ 20
References ........ 22
NMR Spectra ........ 23

## General Information

All purchased chemicals were of reagent grade and all anhydrous solvents were of high-purity grade and used as received from commercial suppliers. The glassware was dried under vacuum with a flameless heat gun, and all reactions were carried out under an argon atmosphere. Reaction progress was monitored by thin layer chromatography (TLC) performed on aluminum plates coated with silica gel F254 with a 0.25 mm thickness. Visualization was performed with UV light and staining with developing agents, followed by heating. The staining solutions were 3-methoxyphenol ethanol solution containing sulfuric acid, cerium sulfate-ammonium molybdate (CAM), acidic ninhydrin-acetone solution and basic potassium permanganate solution. Flash column chromatography was performed using Sigma Aldrich silica gel high purity grade 60 Å (230–400 mesh). Yields refer to spectroscopically homogeneous materials, unless otherwise stated.

$^{1}$H, $^{13}$C and $^{31}$P-NMR as well as 2D-spectra (COSY, HSQC and HMBC) were recorded on a Bruker 400 (400 MHz) or a Bruker Ascend 400 (400 MHz) spectrometers; the chemical shifts are expressed in parts per million (pmm) referenced to solvent signals. Data for $^{1}$H RMN are reported as follows: δ, chemical shift; multiplicity (recorded as br, broad; app, apparent; s, singlet; d, doublet; t, triplet; q, quadruplet; and m, multiplet), coupling constants (J in Hertz,

Hz) and integration. Data for $^{13}C$ NMR and $^{31}P$ NMR are reported as the chemical shifts, expressed in parts per million. High resolution mass spectrometry (HRMS) was performed on a Waters Xevo G2-XS Q-TOF spectrometer with an Acquity H-class UPLC. Mass spectra (MS) were recorded on a 1260 Infinity II LC System coupled to an Agilent LC/MSD iQ Mass Selective Detector (G6160AA). Ionization was done by Electrospray (ESI). Mass spectrum data are reported as m/z. Infrared spectra (IR) were obtained on a Perkin Elmer Spectrum 100 FTIR spectrophotometer with PIKE MIRacle ATR cell and are reported in terms of frequency of absorption (v, $cm^{-1}$). Optical rotation was determined on a Schmidt & Haensch UniPol L 1000 polarimeter and is reported as follows: $[\alpha]_{20}^{D}$ : (c, solvent), where c = concentration is expressed in g/100 mL.

## Synthesis of Compounds 1-3

### 2-Azido-2-deoxy-3,4,6-tri-*O*-(2-naphthyl)methyl-α-D-glucopyranosyl-(1→6)-1-*O*-allyl-2,3,4,5-tetra-*O*-(2-naphthyl)methyl-D-*myo*-inositol (6)

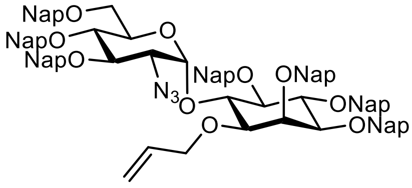


A solution of a 11/1 α:β mixture of *pseudo*-disaccharide **10**[1] (0.0629 mmol) and NaOMe (10 mg, 0.189 mmol) in anhydrous MeOH (2 mL) was stirred at 40 °C for 3 h. The reaction was neutralized with Amberlite IR 120 ($H^+$) resin, filtered, and concentrated *in vacuo*. The resulting residue was purified by flash silica gel column chromatography to give the desired triol in 94% yield over two steps (57.5 mg, 0.0594 mmol) as a white solid. The α and β anomers were separated during the column; $R_f$ α-anomer= 0.31, $R_f$ β-anomer= 0.2 (hexane/EtOAc, 3:7).

NaH (16.9 mg, 0.424 mmol, 60% in mineral oil) was added to a solution of isolated α-anomer (68.5 g, 0.0708 mmol) and TBAI (118 mg, 0.318 mmol) in DMF (1.8 mL) at 0 °C. After 20 min, 2-(Bromomethyl)naphthalene (70.4 mg, 0.318 mmol) was added, and the reaction mixture was stirred at room temperature overnight. The reaction was quenched with water, and volatiles were removed under reduced pressure. The remaining was dissolved with ethyl acetate, and the solution was extracted with water. The layers were separated, and the aqueous phase was extracted with ethyl acetate. The organic layers were combined, washed with brine, dried with $Na_2SO_4$, filtered, and evaporated. The resulting residue was purified by flash silica gel column chromatography to afford **6** in 92% (90.4 mg, 0.0651 mmol) yield as a white solid; $R_f$ = 0.37 (hexane/EtOAc, 8:2).

$[\alpha]_D^{20}$**:** 43.5 (c = 1, $CHCl_3$).

**IR (ATR) v($cm^{-1}$)** 3056, 2866, 2106 (N=N=N), 1603, 1510, 1461, 1365, 1347, 1272, 1216, 1172, 1125, 1055, 953, 894, 856, 816, 753, 667.

**$^1H$ NMR (400 MHz, $CDCl_3$) δ (ppm)** 7.65 – 7.89 (m, 23H, Ar), 7.61 (t, *J* = 7.5 Hz, 4H, Ar), 7.33 – 7.53 (m, 26H, Ar), 7.17 – 7.25 (m, 2H, Ar), 7.15 (s, 1H, Ar), 6.83 (dd, *J* = 8.5, 1.6 Hz, 1H, Ar), 5.93 – 6.04 (m, 1H, -C<u>H</u>= Allyl), 5.88 (d, *J* = 3.7 Hz, 1H, Glc-1), 5.29 (dd, *J* = 17.2, 1.6 Hz, 1H, =C$\underline{H}_{2a}$ Allyl), 5.16 – 5.25 (m, 3H, =C$\underline{H}_{2b}$ Allyl, C$\underline{H}_2$ Nap), 4.98 – 5.14 (m, 6H, C$\underline{H}_2$ Nap), 4.93 (d, *J* = 11.6 Hz, 1H, C$\underline{H}_2$ Nap), 4.78 – 4.90 (m, 2H, C$\underline{H}_2$ Nap), 4.76 (d, *J* = 11.2 Hz, 1H, C$\underline{H}_2$ Nap), 4.63 (d, *J* = 12.3 Hz, 1H, C$\underline{H}_2$ Nap), 4.44 (t, *J* = 9.6 Hz, 1H, Ino-6), 4.27 – 4.40 (m,

2H, Ino-4, C$\underline{H}_2$ Nap), 4.15 – 4.23 (m, 2H, Ino-2, Glc-5), 4.04 – 4.11 (m, 3H, -C$\underline{H}_2$- Allyl, Glc-3), 3.77 – 3.86 (m, 1H, Glc-4), 3.53 – 3.62 (m, 2H, Ino-3, Ino-5), 3.41 – 3.50 (m, 2H, Ino-1, Glc-2), 3.34 (dd, *J* = 11.0, 2.0 Hz, 1H, Glc-6a), 3.14 (dd, *J* = 10.9, 2.2 Hz, 1H, Glc-6b).

**$^{13}$C NMR (101 MHz, $CDCl_3$) δ (ppm)** 136.4, 136.2, 136.0, 135.81, 135.79, 135.7, 135.3 ($C_q$ Ar), 134.4 (-$\underline{C}$H= Allyl), 133.4, 133.33, 133.30, 133.27, 133.2, 133.12, 133.08, 133.06, 132.98, 132.95, 132.8, 132.7 ($C_q$ Ar), 128.4, 128.32, 128.27, 128.2, 128.13, 128.09, 128.05, 128.03, 128.01, 127.9, 127.85, 127.82, 127.74, 127.72, 127.67, 127.0, 126.8, 126.7, 126.7, 126.5, 126.44, 126.39, 126.29, 126.26, 126.2, 126.12, 126.09, 126.03, 125.96, 125.94, 125.92, 125.91, 125.9, 125.81, 125.78, 125.7, 125.6, 125.5 (C Ar), 117.1 (=$\underline{C}H_2$ Allyl), 97.9 (Glc-1), 82.2 (Ino-1), 82.1 (Ino-4), 81.6 (Ino-5), 80.8 (Ino-3), 80.4 (Glc-3), 78.2 (Glc-4), 76.0 ($\underline{C}H_2$ Nap), 75.6 (Ino-6), 75.5 ($\underline{C}H_2$ Nap), 75.4 ($\underline{C}H_2$ Nap), 74.8 ($\underline{C}H_2$ Nap), 74.2 ($\underline{C}H_2$ Nap), 73.5 ($\underline{C}H_2$ Nap), 73.0 ($\underline{C}H_2$ Nap), 72.9 (Ino-2), 71.1 (-$\underline{C}H_2$- Allyl), 70.2 (Glc-5), 67.7 (Glc-6), 63.6 (Glc-2).

**HRMS:** $[M+Na]^+$ cald 1410.5820; found 1410.5811.

**ESI-MS:** 1410.3 $[M-Na]^+$, 1286.3 $[M-K]^+$.

**2-Azido-2-deoxy-3,4,6-tri-*O*-(2-naphthyl)methyl-α-D-glucopyranosyl-(1→6)-2,3,4,5-tetra-*O*-(2-naphthyl)methyl-D-*myo*-inositol (11)**

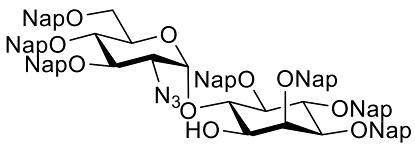


To a solution of the allylated *pseudo*-disaccharide **6** (43.4 mg, 0.0312 mmol) in a 1:1 DCM/MeOH anhydrous mixture (1 mL), $PdCl_2$ (2.8 mg, 0.0156 mmol) was added at room temperature. After 3 h, the mixture was filtered through a pad of Celite, and the volatiles were evaporated *in vacuo*. The resulting oil was purified by flash silica gel column chromatography to obtain **11** in 85% yield (35.8 mg, 0.0265 mmol) as a white solid; $R_f$ = 0.39 (hexane/EtOAc, 7:3).

$[\alpha]_D^{20}$**:** 35.8 (c = 1, $CHCl_3$).

**IR (ATR) v(cm$^{-1}$)** 3458 (O-H), 3056, 3019, 2925, 2864, 2108 (N=N=N), 1603, 1510, 1462, 1366, 1344, 1272, 1248, 1216, 1171, 1124, 1044, 953, 893, 855, 815, 750, 667.

**$^{1}$H NMR (400 MHz, $CDCl_3$) δ (ppm)** 7.63 – 7.89 (m, 23H, Ar), 7.35 – 7.69 (m, 26H, Ar), 7.21 (s, 1H, Ar), 7.14 (d, *J* = 8.4 Hz, 1H, Ar), 6.91 (d, *J* = 8.4 Hz, 1H, Ar), 5.56 (d, *J* = 3.6 Hz, 1H, Glc-1), 5.15 – 5.29 (m, 3H, C$\underline{H}_2$ Nap), 4.97 – 5.12 (m, 4H, C$\underline{H}_2$ Nap), 4.84 – 4.92 (m, 3H, C$\underline{H}_2$ Nap), 4.78 & 4.38 (ABq, *J* = 11.5 Hz, 2H, C$\underline{H}_2$ Nap), 4.50 & 3.97 (ABq, *J* = 12.2 Hz, 2H, C$\underline{H}_2$ Nap), 4.32 (t, *J* = 9.6 Hz, 1H, Ino-4), 4.06 – 4.21 (m, 3H, Ino-1, Glc-3, Ino-6), 4.03 (d, *J* = 10.2 Hz, 1H, Glc-5), 3.82 (t, *J* = 9.5 Hz, 1H, Glc-4), 3.72 (d, *J* = 9.4 Hz, 1H, Ino-2), 3.61 – 3.69 (m, 2H, Glc-2, Ino-3), 3.54 (t, *J* = 9.3 Hz, 1H, Ino-5), 3.20 (d, *J* = 10.8 Hz, 1H, Glc-6a), 3.05 (d, *J* = 10.8 Hz, 1H, Glc-6b).

**$^{13}$C NMR (101 MHz, $CDCl_3$) δ (ppm)** 136.2, 136.1, 136.0, 135.6, 135.5, 135.4, 135.1, 133.36, 133.35, 133.3, 133.2, 133.12, 133.09, 133.0, 132.9, 132.8 ($C_q$ Ar), 128.4, 128.3, 128.2, 128.14, 128.12, 128.09, 128.06, 128.04, 128.01, 127.98, 127.9, 127.83, 127.77, 127.73, 127.71, 127.68, 127.0, 126.82, 126.81, 126.6, 126.30, 126.26, 126.23, 126.21, 126.15,

126.12, 126.08, 126.05, 126.01, 125.93, 125.90, 125.88, 125.80, 125.75, 125.7, 125.5 (C Ar), 98.8 (Glc-1), 82.1 (Ino-4), 81.3 (Ino-5), 81.02 (Ino-1), 80.97 (Glc-3), 80.9 (Ino-3), 78.1 (Glc-4), 76.8 (Ino-6), 76.0 (C̲H$_2$ Nap), 75.6 (C̲H$_2$ Nap), 75.2 (C̲H$_2$ Nap), 75.0 (C̲H$_2$ Nap), 74.9 (C̲H$_2$ Nap), 73.8 (Ino-2), 73.4 (C̲H$_2$ Nap), 73.1 (C̲H$_2$ Nap), 71.0 (Glc-5), 67.4 (Glc-6), 64.3 (Glc-2).

**HRMS:** [M+Na]$^+$ cald 1370.5507; found 1370.5432.

**ESI-MS:** 1371.3 [M-Na]$^+$.

**2-Azido-2-deoxy-3,4,6-tri-*O*-(2-naphthyl)methyl-α-D-glucopyranosyl-(1→6)-2,3,4,5-tetra-*O*-(2-naphthyl)methyl-1-*O*-(2-oleoyl-1-stearoyl-sn-glycero-3-phosphonate)-D-*myo*-inositol (12)**

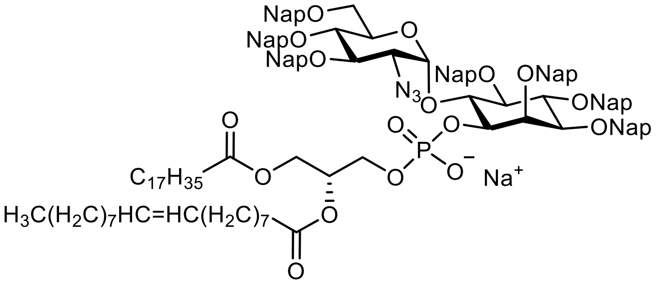


Alcohol **11** (79.7 mg, 0.0591 mmol) and H-phosphonate **9**[2] (88% purity, 85.7 mg, 0.0946 mmol) were co-evaporated with anhydrous pyridine (3 x 3 mL) and placed under high vacuum for 1 h. The residue was dissolved in anhydrous pyridine (4.5 mL) and PivCl (18.8 µL, 0.154 mmol) was added. The reaction mixture was stirred for 1.5 h at room temperature before water (254 µL) and iodine (45 mg, 0.177 mmol) were added. The reaction mixture was stirred for additional 50 min, and solid $Na_2S_2O_3$ was added until the color of the reaction changed from orange to yellow. Volatiles were removed under reduced pressure. The remaining was triturated with DCM to remove $Na_2S_2O_3$, and concentrated. Purification was performed by flash silica gel column chromatography (eluting with MeOH/DCM 1:90 and gradually increasing the polarity to 1:10). The obtained compound was dissolved in chloroform, and Amberlite IR 120 (Na$^+$) form resin was added. After stirring for 5 min, the solution was filtered and concentrated to afford the lipidated *pseudo*-disaccharide **12** in 98% yield (0.119 mg, 0.0581 mmol) as a white solid; $R_f$ = 0.54 (MeOH/DCM, 1:10).

$[\alpha]_D^{20}$**:** 37.7 (c = 1, $CHCl_3$).

**IR (ATR) ν(cm$^{-1}$)** 3057, 2926, 2855, 2110 (N=N=N), 1742 (C=O), 1604, 1510, 1466, 1366, 1246, 1125, 1047, 893, 855, 816, 752.

**$^1$H NMR (400 MHz, $CDCl_3$) δ (ppm)** 7.90 (s, 1H, Ar), 7.50 – 7.84 (m, 22H, Ar), 7.27 – 7.47 (m, 22H, Ar), 7.09 – 7.20 (m, 3H, Ar), 6.79 (d, *J* = 8.5 Hz, 1H, Ar), 5.69 (s, 1H, Glc-1), 5.09 – 5.42 (m, 8H, -CH̲=CH̲- Oleoyl, CH̲ sn-2, CH̲$_2$ Nap), 4.98 – 5.08 (m, 2H, CH̲$_2$ Nap), 4.77 – 4.96 (m, 4H, CH̲$_2$ Nap), 4.64 – 4.75 (m, 2H, CH̲$_2$ Nap, Ino-2), 4.44 – 4.62 (m, 3H, CH̲$_2$ Nap, Ino-1, Ino-6), 4.05 – 4.37 (m, 9H, CH̲$_2$ Nap, CH̲$_2$ sn-1, CH̲$_2$ sn-3, Glc-3, Ino-4, Glc-5), 3.69 – 3.83 (m, 2H, Ino-3, Glc-4), 3.57 (t, *J* = 8.7 Hz, 1H, Ino-5), 3.45 – 3.52 (m, 1H, Glc-2), 3.32 (d, *J* = 10.7 Hz, 1H, Glc-6a), 3.13 (d, *J* = 10.8 Hz, 1H, Glc-6b), 2.89 – 2.99 (m, 1H, $Et_3N$ residue), 2.23 (q, *J* = 8.4 Hz, 4H, 2 x O-COCH̲$_2$-CH$_2$- lipid), 1.91 – 2.05 (m, 4H, -CH̲$_2$-CH=CH-CH̲$_2$- Oleoyl), 1.52 (b s, 4H, 2 x O-COCH$_2$-CH̲$_2$- lipid), 1.09 – 1.39 (m, 57H, CH̲$_2$ lipid), 0.84 – 0.93 (m, 7H, 2 x CH̲$_3$ lipid).

**$^{13}$C NMR (101 MHz, $CDCl_3$) δ (ppm)** 173.4 ($\underline{C}$=O), 173.1 ($\underline{C}$=O), 136.5, 136.1, 135.8, 135.7, 135.5, 135.2, 133.4, 133.33, 133.29, 133.23, 133.15, 133.10, 133.07, 133.01, 132.97, 132.9, 132.85, 132.75 ($C_q$ Ar), 130.0 (-$\underline{C}$H=CH- Oleoyl), 129.8 (-CH=$\underline{C}$H- Oleoyl), 128.3, 128.21, 128.17, 128.14, 128.10, 128.05, 128.02, 128.00, 127.94, 127.91, 127.86, 127.79, 127.76, 127.73, 127.71, 127.6, 126.9, 126.7, 126.6, 126.5, 126.4, 126.3, 126.2, 126.4, 126.11, 126.09, 126.04, 126.01, 125.96, 125.92, 125.88, 125.85, 125.78, 125.7, 125.5 (C Ar), 97.7 (Glc-1), 81.9 (Ino-4), 81.0 (Ino-5), 80.7 (Ino-3), 80.16 (Glc-3), 79.6 (Ino-1), 78.2 (Glc-4), 76.7 (Ino-2), 76.0 ($\underline{C}H_2$ Nap), 75.7 ($\underline{C}H_2$ Nap), 75.6 ($\underline{C}H_2$ Nap), 75.3 ($\underline{C}H_2$ Nap), 74.8 (Ino-6), 73.4 ($\underline{C}H_2$ Nap), 72.8 ($\underline{C}H_2$ Nap), 70.5 (Glc-5), 69.74 (C$\underline{H}$ sn-2), 69.76 ($\underline{C}H_2$ Nap), 67.69 (Glc-6), 65.3 ($\underline{C}H_2$ sn-3), 63.5 (Glc-2), 62.0 ($\underline{C}H_2$ sn-1), 34.2 (O-CO$\underline{C}H_2$-$CH_2$ lipid), 34.1 (O-CO$\underline{C}H_2$-$CH_2$ lipid), 32.1, 32.0, 29.90, 29.87, 29.8, 29.71, 29.67, 29.51, 29.48, 29.46, 29.41, 29.38, 29.32, 29.28, 29.2 ($\underline{C}H_2$ lipid), 27.34 (-$\underline{C}H_2$-CH=CH-$CH_2$- Oleoyl), 27.33 (-$CH_2$-CH=CH-$\underline{C}H_2$- Oleoyl), 25.0 (O-CO$CH_2$-$\underline{C}H_2$- lipid), 24.9 (O-CO$CH_2$-$\underline{C}H_2$- lipid), 22.84 ($\underline{C}H_2$ lipid), 22.82 ($\underline{C}H_2$ lipid), 14.29 (2 x $\underline{C}H_3$ lipid).

**$^{31}$P NMR (162 MHz, $CDCl_3$) δ (ppm) -**1.02.

**HRMS:** $[M-H]^-$ cald 2031.0625; found 2031.0488.

**2-Acetamido-2-deoxy-α-D-glucopyranosyl-(1→6)-1-*O*-(2-oleoyl-1-stearoyl-sn-glycero-3-phosphonate)-D-*myo*-inositol (1)**

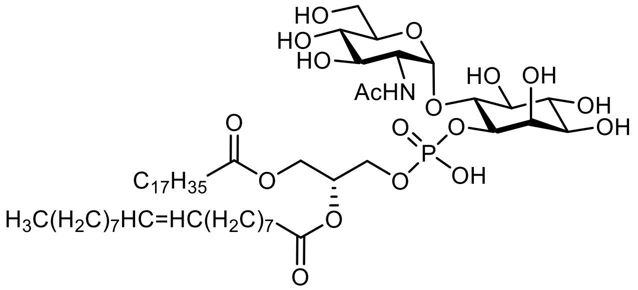


The glycolipid **12** (45 mg, 0.0219 mmol) was mixed with 0.83 mL of a 1M solution of $P(CH_3)_3$ in THF. Water (170 µL) was added, and the reaction was stirred at room temperature for 1.5 h. Volatiles were removed under reduced pressure and the residue was dissolved in a 2:1 pyridine/acetic anhydride mixture. The reaction was stirred for additional 2 h before it was concentrated *in vacuo*. Purification was performed by flash silica gel column chromatography (eluting with MeOH/DCM 1:90 and gradually increasing the polarity to 1:25). The obtained *N*-acetylated compound **13** (43.6 mg, 0.0213 mmol) was dissolved in a 3:1 DCM/MeOH mixture (1 mL), and DDQ (50.7 mg, 0.223 mmol) was added. The reaction mixture was stirred at room temperature for 3 h, and volatiles were removed under vacuum. The residue was purified by LH20 size exclusion chromatography eluting with MeOH/$CHCl_3$ 9:1 followed by trituration with MeOH to afford the final GPI-fragment **1** in 62% yield (14.5 mg, 0.0136 mmol) as a slightly yellow solid.

**$^{1}$H NMR (400 MHz, $CDCl_3$) δ (ppm)** 5.00 – 5.09 (m, 2H, -C$\underline{H}$=C$\underline{H}$- Oleoyl), 4.91 – 5.00 (m, 1H, C$\underline{H}$ sn-2), 4.69 (d, *J*= 3.4 Hz, 1H, Glc-1), 4.09 – 4.16 (m, 1H, C$\underline{H}_{2a}$ sn-1), 3.82 – 3.92 (m, 3H, C$\underline{H}_{2b}$ sn-1), 3.71 – 3.79 (m, 2H, C$\underline{H_2}$ sn-3), 3.64 – 3.70 (m, 2H, Glc-2), 3.53 – 3.59 (m, 1H, Glc-6a), 3.32 – 3.51 (m, 4H, Glc-6b), 3.12 – 3.19 (m, 1H), 3.04 – 3.12 (m, 2H), 1.98 – 2.09 (m, 4H, 2 x O-COC$\underline{H}_2$-$CH_2$- lipid), 1.79 (s, 3H, C$\underline{H}_3$ Ac), 1.69 – 1.75 (m, 4H, -C$\underline{H}_2$-CH=CH-C$\underline{H}_2$- Oleoyl), 1.26 – 1.34 (m, 4H, 2 x O-CO$CH_2$-C$\underline{H}_2$- lipid), 0.92 – 1.07 (m, 58H, C$\underline{H}_2$ lipid), 0.58 (t, *J* = 6.7 Hz, 8H, 2 x C$\underline{H}_3$ lipid).

**$^{13}C$ NMR (101 MHz, $CDCl_3$) δ (ppm)** 174.0 (C=O), 173.7 (C=O), 129.6 (-CH=CH- Oleoyl), 129.3 (-CH=CH- Oleoyl), 99.0 (Glc-1), 81.4, 73.7, 72.8, 71.9, 71.2, 70.6, 70.2 (CH sn-2), 63.4 ($CH_2$ sn-3),62.6 ($CH_2$ sn-1), 60.8 (Glc-6), 53.6 (Glc-2), 33.8 (O-CO$CH_2$-$CH_2$- lipid), 33.7 (O-CO$CH_2$-$CH_2$- lipid), 31.5, 29.4, 29.33, 29.27, 29.22, 29.15, 29.1, 28.93, 28.90, 28.87, 28.83, 28.77, 28.7, 26.78 (-$CH_2$-CH=CH-$CH_2$- Oleoyl), 26.77 (-$CH_2$-CH=CH-$CH_2$- Oleoyl), 24.6 (O-CO$CH_2$-$CH_2$- lipid), 24.5 (O-CO$CH_2$-$CH_2$- lipid), 22.2 ($CH_3$ Ac), 21.9 ($CH_2$ lipid), 13.5 (2 x $CH_3$ lipid).

**$^{31}P$ NMR (162 MHz, $CDCl_3$) δ (ppm)** -0.56.

**HRMS:** $[M-H]^-$ cald 1066.6443; found 1066.6547.

**2-Acetamido-2-deoxy-3,4,6-tri-O-acetyl-α-D-glucopyranosyl-(1→6)-1-*O*-(2-oleoyl-1-stearoyl-sn-glycero-3-phosphonate)-2,3,4,5-tetra-O-acetyl-D-*myo*-inositol (2)**

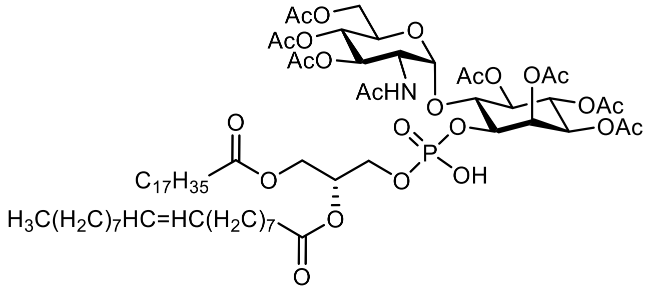


Peracetylation of compound **1** was performed using acetic anhydride and pyridine. The glycolipid **1** (4 mg, 3.75 mmol) was dissolved in a 1.5 mL mixture of pyridine/acetic anhydride (2:1) and stirred overnight at room temperature. After removing the solvent by evaporation, the residue was dissolved chloroform (10 mL) and extracted with aqueous sat. $NaHCO_3$, and water. The organic phase was separated and evaporated. The product was purified on a LH-20 column using $CHCl_3$/MeOH (2:1) as eluent to obtain 3.5 mg (69%) of the peracetylated glycolipid **2**.

**MALDI-MS:** $[M-H]^-$ cald 1361,7261; found 1361,847.

**2-Amino-2-deoxy-α-D-glucopyranosyl-(1→6)-1-*O*-(2-oleoyl-1-stearoyl-sn-glycero-3-phosphonate)-D-*myo*-inositol (3)**

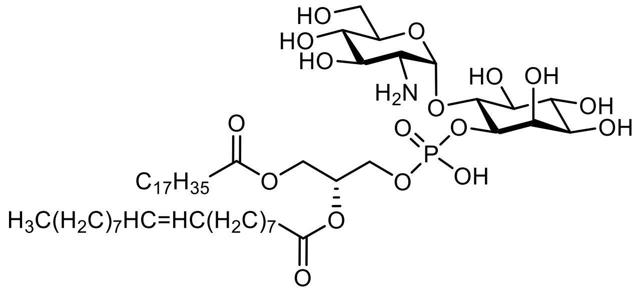


DDQ (79.0 mg, 0.348 mmol) was added to a solution of the protected glycolipid **12** (68.1 mg, 0.0331 mmol) in a 3:1 DCM/MeOH mixture (1.6 mL). The reaction mixture was stirred at room temperature for 5 h before volatiles were removed under reduced pressure. The residue was purified by LH20 size exclusion chromatography eluting with MeOH/$CHCl_3$ 9:1. To the obtained deprotected glycolipid (26.1 mg, 0.0243 mmol), 0.49 mL of a 1M solution of $P(CH_3)_3$ in THF were added followed by water (89 µL). After 2h, the reaction was concentrated under vacuum, and purification was performed by LH20 size exclusion chromatography eluting with

MeOH/$CHCl_3$/$H_2O$ 3:3:1 followed by trituration with MeOH to give the final GPI-fragment **3** in 47% yield (16.1 mg, 0.0157 mmol) as a slightly yellow solid.

**$^1$H NMR (400 MHz, $CDCl_3$) δ (ppm)** 5.24 (d, *J*= 4.0 Hz, 1H, Glc-1), 5.00 – 5.10 (m, 2H, -CH=CH- Oleoyl), 4.93 – 5.01 (m, 1H, CH sn-2), 4.09 – 4.16 (m, 1H, $CH_{2a}$ sn-1), 3.83 – 3.94 (m, 2H, $CH_{2b}$ sn-1), 3.61 – 3.83 (m, 5H, $CH_2$ sn-3, Glc-5), 3.49 – 3.59 (m, 2H, Glc-3, Glc-6a), 3.43 (dd, *J* = 12.5, 4.8 Hz, 1H, Glc-6b), 3.37 (t, *J* = 9.7 Hz, 1H), 3.10 – 3.19 (m, 2H, Glc-4), 3.03 – 3.05 (m, 1H),2.90 (dd, *J*= 10.5, 4.2 Hz, 1H, Glc-2), 1.96 – 2.12 (m, 4H, 2 x O-CO$CH_2$-$CH_2$- lipid), 1.65 – 1.79 (m, 4H, -$CH_2$-CH=CH-$CH_2$- Oleoyl), 1.25 – 1.37 (m, 4H, 2 x O-CO$CH_2$-$CH_2$- lipid), 0.99 (d, *J* = 16.1 Hz, 53H, $CH_2$ lipid), 0.59 (app t, *J* = 6.6 Hz, 6H, 2 x $CH_3$ lipid).

**$^{13}$C NMR (101 MHz, $CDCl_3$) δ (ppm)** 174.0 (C=O), 173.7 (C=O), 129.6 (-CH=CH- Oleoyl), 129.3 (-CH=CH- Oleoyl), 94.7 (Glc-1), 76.3, 72.7, 72.2, 71.9, 71.5 (Glc-5), 70.6, 70.04 (CH sn-2), 69.95, 69.8 (Glc-3), 69.4 (Glc-4), 63.3 ($CH_2$ sn-3), 62.5 ($CH_2$ sn-1), 60.2 (Glc-6), 53.8 (Glc-2), 33.8 (O-CO$CH_2$-$CH_2$- lipid), 33.7 (O-CO$CH_2$-$CH_2$- lipid), 31.5, 31.5, 29.40, 29.35, 29.31, 29.26, 29.2, 29.1, 29.0, 28.93, 28.90, 28.85, 28.80, 28.7 ($CH_2$ lipid), 26.81 (-$CH_2$-CH=CH-$CH_2$- Oleoyl), 26.80 (-$CH_2$-CH=CH-$CH_2$- Oleoyl), 24.6 (O-CO$CH_2$-$CH_2$- lipid), 24.5 (O-CO$CH_2$-$CH_2$- lipid), 22.3 ($CH_2$ lipid), 13.5 (2 x $CH_3$ lipid).

**$^{31}$P NMR (162 MHz, $CDCl_3$) δ (ppm) -**0.01.

**HRMS:** $[M-H]^-$ cald 1024.6338; found 1024.6212.

## Synthesis of compound 4

### 2-Azido-2-deoxy-3,4,6-tri-*O*-(2-naphthyl)methyl-α-D-glucopyranosyl-(1→6)-1-*O*-allyl-3,4,5-tri-*O*-(2-naphthyl)methyl-2-*O*-(p-methoxybenzyl)-D-*myo*-inositol (16)

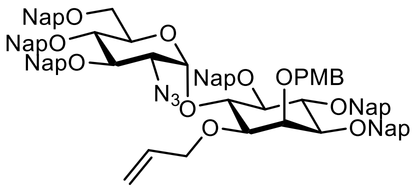


A solution of glycosyl acceptor **15**[3] (30.8 mg, 0.0405 mmol) and glycosyl donor **14**[1] (40 mg, 0.0606 mmol) in a 6:1 $Et_2O$/DCM anhydrous mixture (1 mL) was stirred at room temperature for 10 min. The mixture was cooled to 0 °C, and TMSOTf (2.1 µL, 0.0115 mmol) was added. After stirring for 2 h at room temperature, the reaction was quenched with $Et_3N$ and concentrated under reduced pressure. The obtained residue was purified by flash silica gel column chromatography to afford the corresponding *pseudo*-disaccharide as a 7:1 α/β mixture that could not be separated; $R_f$ = 0.34 (hexane/EtOAc, 7:3). The product was dissolved in an 11:1 MeOH/DCM anhydrous mixture and 61 µL (0.121 mmol) of a 5M NaOMe solution in MeOH was added. The reaction was stirred at 40 °C for 1 h before it was neutralized with Amberlite IR 120 ($H^+$) resin, filtered and concentrated *in vacuo*. The resulting residue was purified by flash silica gel column chromatography to give the desired triol in 77% yield (29.6 mg, 0.0312 mmol) as a white solid. The α and β anomers were separated during the column; $R_f$ α-anomer= 0.40, $R_f$ β-anomer= 0.27 (hexane/EtOAc, 3:7).

NaH (20.3 mg, 0.509 mmol, 60% in mineral oil) was added to a solution of the isolated α-anomer (80.4 g, 0.0848 mmol) and TBAI (141 mg, 0.382 mmol) in DMF (1.2 mL) at 0 °C. After 20 min, 2-(Bromomethyl)naphthalene (84.4 mg, 0.382 mmol) was added, and the reaction

mixture was stirred at room temperature for 2 days. The reaction was quenched with water, and volatiles were removed under reduced pressure. The remaining was dissolved with ethyl acetate, and the solution was extracted with water. The layers were separated, and the aqueous phase was extracted with ethyl acetate. Organic layers were combined, washed with brine, dried with $Na_2SO_4$, filtered, and evaporated. The resulting residue was purified by flash silica gel column chromatography to give **16** in 76% (88.2 mg, 0.0644 mmol) yield as a white solid; $R_f$ = 0.34 (hexane/EtOAc, 8:2).

$[\alpha]_D^{20}$: 41.4 (c = 1, $CHCl_3$).

**IR (ATR) ν(cm$^{-1}$)** 3056, 2865, 2106 (N=N=N), 1603, 1511, 1462, 1365, 1248, 1172, 1125, 1037, 854, 893, 856, 817, 753.

**$^1$H NMR (400 MHz, $CDCl_3$) δ (ppm)** 7.66 – 7.88 (m, 14H, Ar), 7.57 – 7.66 (m, 4H, Ar), 7.31 – 7.51 (m, 22H, Ar), 7.13 – 7.24 (m, 3H, Ar), 6.86 – 6.92 (m, 2H, Ar PMB), 6.83 (dd, *J* = 8.4, 1.7 Hz, 1H, Ar), 5.92 – 6.05 (m, 1H, -C<u>H</u>= Allyl), 5.84 (d, *J*= 3.7 Hz, 1H, Glc-1), 5.30 (dd, *J* = 17.2, 1.8 Hz, 1H, =C$\underline{H}_{2a}$ Allyl), 5.14 – 5.24 (m, 3H, =C$\underline{H}_{2b}$ Allyl, C$\underline{H}_2$ Nap), 5.08 (ABq, *J* = 11.0 Hz, 2H, C$\underline{H}_2$ Nap), 4.99 (d, *J* = 10.9 Hz, 1H, C$\underline{H}_2$ Nap), 4.92 (d, *J* = 11.6 Hz, 1H, C$\underline{H}_2$ Nap), 4.79 – 4.89 (m, 4H, C$\underline{H}_2$ PMB, C$\underline{H}_2$ Nap), 4.76 & 4.32 (ABq, *J* = 11.2 Hz, 2H, C$\underline{H}_2$ Nap), 4.63 & 4.20 (ABq, *J* = 12.2 Hz, 2H, C$\underline{H}_2$ Nap), 4.39 (t, *J* = 9.6 Hz, 1H, Ino-6), 4.27 – 4.35 (m, 1H, Ino-4), 4.15 – 4.22 (m, 1H, Glc-5), 4.12 – 4.15 (m, 1H, Ino-2), 4.03 – 4.11 (m, 3H, -C$\underline{H}_2$- Allyl, Glc-3), 3.81 (s, 3H, OC$\underline{H}_3$ PMB, Glc-4), 3.50 – 3.60 (m, 2H, Ino-3, Ino-5), 3.38 – 3.47 (m, 2H, Ino-1, Glc-2), 3.33 (dd, *J* = 11.0, 2.0 Hz, 1H, Glc-6a), 3.13 (dd, *J* = 11.0, 2.3 Hz, 1H, Glc-6b).

**$^{13}$C NMR (101 MHz, $CDCl_3$) δ (ppm)** 159.3, 136.2, 136.1, 135.89, 135.86, 135.7, 135.3 ($C_q$ Ar), 134.5 (-<u>C</u>H= Allyl), 133.40, 133.38, 133.35, 133.3, 133.21, 133.15, 133.11, 133.08, 133.01, 132.97, 132.87, 132.77, 131.0 ($C_q$ Ar), 129.7, 128.31, 128.26, 128.13, 128.10, 128.07, 128.03, 128.01, 127.93, 127.90, 127.85, 127.83, 127.80, 127.75, 127.73, 127.68, 127.0, 126.8, 126.7, 126.4, 126.31, 126.26, 126.2, 126.13, 126.09, 126.03, 125.95, 125.93, 125.91, 125.87, 125.80, 125.77, 125.7, 125.6, 125.5 (C Ar), 117.0 (=$\underline{C}H_2$ Allyl), 113.8 (C Ar, PMB), 97.9 (Glc-1), 82.2 (Ino-1), 82.1 (Ino-4), 81.6 (Ino-5), 80.9 (Ino-3), 80.4 (Glc-3), 78.2 (Glc-4), 76.0 ($\underline{C}H_2$ Nap), 75.6 (Ino-6), 75.5 ($\underline{C}H_2$ Nap), 75.4 ($\underline{C}H_2$ Nap), 74.8 ($\underline{C}H_2$ Nap), 73.9 ($\underline{C}H_2$ PMB), 73.5 ($\underline{C}H_2$ Nap), 72.9 ($\underline{C}H_2$ Nap), 72.6 (Ino-2), 71.0 (-$\underline{C}H_2$- Allyl), 70.3 (Glc-5), 67.8 (Glc-6), 63.6 (Glc-2), 55.4 (O$\underline{C}H_3$ PMB).

**HRMS:** [M+Na]$^+$ cald 1390.5769; found 1390.6027; [M+$NH_4$]$^+$ cald 1385.6215; found 1385.6481.

**2-Azido-2-deoxy-3,4,6-tri-*O*-(2-naphthyl)methyl-α-D-glucopyranosyl-(1→6)-1-*O*-allyl-3,4,5-tri-*O*-(2-naphthyl)methyl-D-*myo*-inositol (17)**

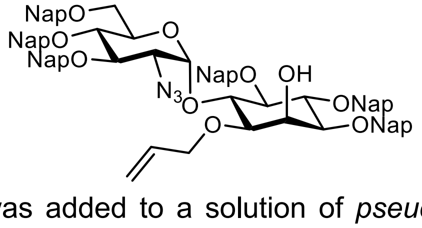


TFA (0.15 mL, 1.42 mmol) was added to a solution of *pseudo*-disaccharide **16** (88.3 mg, 0.0645 mmol) in anhydrous DCM (1 mL) at 0 °C, and it was stirred for 40 min. The reaction was quenched with $Et_3N$ and concentrated under reduced pressure. The crude product was

purified by flash silica gel column chromatography to obtain **17** in 97% (78.4 mg, 0.0628 mmol) yield as a white solid; $R_f$ = 0.15 (hexane/EtOAc, 7:3).

$[\alpha]_D^{20}$**:** 10.4 (c = 1, $CHCl_3$).

**IR (ATR) ν(cm$^{-1}$)** 3449 (O-H), 3055, 2927, 2106 (N=N=N), 1711, 1604, 1510, 1463, 1366, 1347, 1247, 1172, 1125, 1060, 953, 894, 856, 816, 752.

**$^1$H NMR (400 MHz, $CDCl_3$) δ (ppm)** 7.63 – 7.87 (m, 24H, Ar), 7.30 – 7.57 (m, 34H, Ar), 7.16 – 7.21 (m, 2H, Ar), 6.90 (d, *J* = 8.2 Hz, 1H, Ar), 6.85 (d, *J* = 8.4 Hz, 1H, Ar), 5.92 – 6.05 (m, 1H, -C<u>H</u>= Allyl), 5.76 (d, *J*= 3.8 Hz, 1H, Glc-1), 5.28 (d, *J* = 17.2 Hz, 1H, =C<u>H</u>$_{2a}$ Allyl), 5.11 – 5.22 (m, 4H, =C<u>H</u>$_{2b}$ Allyl, C<u>H</u>$_2$ Nap), 4.85 – 5.08 (m, 9H, C<u>H</u>$_2$ Nap), 4.76 & 4.36 (ABq, *J* = 11.2 Hz, 2H, C<u>H</u>$_2$ Nap), 4.59 – 4.65 (m, 2H, C<u>H</u>$_2$ Nap), 4.10 – 4.33 (m, 10H, C<u>H</u>$_2$ Nap, Ino-2, Ino-4, Glc-5, Ino-6, -C<u>H</u>$_2$- Allyl), 4.06 (t, *J* = 9.6 Hz, 1H, Glc-3), 3.76 – 3.80 (m, 1H, Glc-4), 3.45 – 3.59 (m, 1H, Ino-1, Ino-3, Ino-5), 3.41 (dd, *J*= 10.4, 3.7 Hz, 1H, Glc-2), 3.35 (d, *J* = 10.9 Hz, 1H, Glc-6a), 3.20 (d, *J* = 10.7 Hz, 1H, Glc-6b).

**$^{13}$C NMR (101 MHz, $CDCl_3$) δ (ppm)** 136.1, 135.83, 135.80, 135.6, 135.39, 135.36 ($C_q$ Ar), 134.2 (-<u>C</u>H= Allyl), 133.38, 133.36, 133.33, 133.27, 133.22, 133.19, 133.16, 133.1, 133.03, 132.99, 132.9, 132.8 ($C_q$ Ar), 128.8, 128.5, 128.3, 128.18, 128.15, 128.1, 128.0, 128.0, 127.94, 127.90, 127.83, 127.75, 127.71, 127.68, 126.9, 126.8, 126.6, 126.4, 126.24, 126.20, 126.15, 126.1, 126.05, 125.94, 125.91, 125.86, 125.8, 125.7, 125.7 (C Ar), 118.0 (=<u>C</u>H$_2$ Allyl), 97.7 (Glc-1), 81.6 (Ino-4), 81.3 (Ino-1), 81.2 (Ino-5), 80.2 (Glc-3), 79.6 (Ino-3), 78.3 (Glc-4), 77.5 (<u>C</u>H$_2$ Nap), 77.4 (<u>C</u>H$_2$ Nap), 77.2 (<u>C</u>H$_2$ Nap), 76.8 (<u>C</u>H$_2$ Nap), 76.1 (<u>C</u>H$_2$ Nap), 75.8 (<u>C</u>H$_2$ Nap), 75.0 (Ino-6), 74.8, 73.5, 73.0, 71.2 (-<u>C</u>H$_2$- Allyl), 70.41 (Glc-5), 67.8 (Glc-6), 66.7 (Ino-2), 63.5 (Glc-2).

**HRMS:** $[M+Na]^+$ cald 1270.5194; found 1270.2440; $[M+K]^+$ cald 1286.4933; found 1286.2083.

**2-Azido-2-deoxy-3,4,6-tri-*O*-(2-naphthyl)methyl-α-D-glucopyranosyl-(1→6)-1-*O*-allyl-3,4,5-tri-*O*-(2-naphthyl)methyl-2-*O*-(palmitoyl)-D-*myo*-inositol (7)**

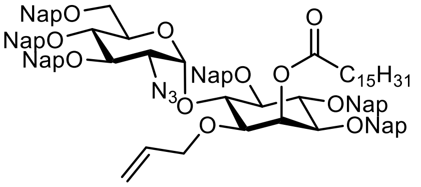


DMAP (3.6 mg, 0.0294 mmol) and DIC (4.6 µL, 0.0294 mmol) were added to a solution of alcohol **17** (22.9 mg, 0.0184 mmol) and palmitic acid (7.5 mg, 0.0294 mmol) in anhydrous DCM (1 mL). After 15 h, the reaction was quenched with water, and the layers were separated. The aqueous phase was extracted with DCM. Organic layers were washed with citric acid (1 M) followed by brine, dried over $Na_2SO_4$, filtered, and concentrated. Crude was purified by flash silica gel column chromatography to afford **7** (23.6 g, 0.0159 mmol) in 86% yield as a white solid; $R_f$ = 0.44 (hexane/EtOAc, 8:2).

$[\alpha]_D^{20}$**:** 47.50 (c = 1, $CHCl_3$).

**IR (ATR) ν(cm$^{-1}$)** 3047, 2926, 2855, 2106 (N=N=N), 1740 (C=O), 1603, 1510, 1467, 1366, 1247, 1126, 1088, 953, 855, 816, 753.

**$^{1}$H NMR (400 MHz, CDCl$_3$) δ (ppm)** 7.77 – 7.85 (m, 8H, Ar), 7.70 – 7.75 (m, 6H, Ar), 7.57 – 7.69 (m, 5H, Ar), 7.38 – 7.52 (m, 21H, Ar), 7.33 (d, *J* = 8.2 Hz, 1H, Ar), 7.16 – 7.25 (m, 3H, Ar), 6.92 (dd, *J* = 8.4, 1.6 Hz, 1H, Ar), 5.94 – 6.07 (m, 2H, -C<u>H</u>= Allyl, Ino-2), 5.81 (d, *J*= 3.7 Hz, 1H, Glc-1), 5.32 (dd, *J* = 17.2, 1.8 Hz, 1H, =C$\underline{H}_{2a}$ Allyl), 5.23 (d, *J* = 10.8 Hz, 2H, =C$\underline{H}_{2b}$ Allyl, C$\underline{H}_2$ Nap), 5.19 (d, *J* = 11.0 Hz, 1H, C$\underline{H}_2$ Nap), 5.12 (ABq, *J* = 11.0 Hz, 2H, C$\underline{H}_2$ Nap), 4.91 – 5.04 (m, 2H, C$\underline{H}_2$ Nap), 4.99 & 4.76 (ABq, *J* = 11.3 Hz, 2H, C$\underline{H}_2$ Nap) 4.83 & 4.38 (ABq, *J* = 11.2 Hz, 2H, C$\underline{H}_2$ Nap), 4.65 & 4.22 (d, *J* = 12.2 Hz, 2H, C$\underline{H}_2$ Nap), 4.28 (dd, *J* = 12.0, 5.6 Hz, 1H, -C$\underline{H}_2$- Allyl), 4.16 – 4.25 (m, 2H, Glc-5, Ino-6), 4.05 – 4.15 (m, 3H, -C$\underline{H}_2$- Allyl, Glc-3, Ino-4), 3.84 (t, *J* = 9.5 Hz, 1H, Glc-4), 3.68 (dd, *J* = 9.8, 2.8 Hz, 1H, Ino-3), 3.57 – 3.66 (m, 2H, Ino-1, Ino-5), 3.43 (dd, *J*= 10.4, 3.7 Hz, 1H, Glc-2), 3.33 (dd, *J* = 10.9, 2.1 Hz, 1H, Glc-6a), 3.22 (dd, *J* = 11.0, 2.4 Hz, 1H, Glc-6b), 2.50 (t, *J* = 7.3 Hz, 2H, O-COC$\underline{H}_2$-CH$_2$- palmitoyl), 1.68 – 1.78 (m, 2H, O-COCH$_2$-C$\underline{H}_2$- palmitoyl), 1.37 – 1.45 (m, 2H, C$\underline{H}_2$ palmitoyl), 1.23 – 1.33 (m, 26H, C$\underline{H}_2$ palmitoyl), 0.92 (t, *J* = 6.8 Hz, 3H, C$\underline{H}_3$ palmitoyl).

**$^{13}$C NMR (101 MHz, CDCl$_3$) δ (ppm)** 173.4 (C=O), 136.0, 135.8, 135.7, 135.6, 135.2, 135.1 (C$_q$ Ar), 134.2 (-<u>C</u>H= Allyl), 133.37, 133.35, 133.3, 133.25, 133.18, 133.15, 133.1, 133.01, 132.99, 132.9, 132.8 (C$_q$ Ar), 128.33, 128.29, 128.18, 128.16, 128.1, 128.03, 128.00, 127.9, 127.78, 127.75, 127.72, 127.68, 127.6, 127.2, 127.0, 126.8, 126.6, 126.4, 126.3, 126.2, 126.13, 126.10, 126.05, 126.03, 125.98, 125.96, 125.9, 125.83, 125.78, 125.75 (C Ar), 117.7 (=<u>C</u>H$_2$ Allyl), 98.0 (Glc-1), 81.8 (Ino-4), 81.0 (Ino-1), 80.3 (Glc-3), 79.2 (Ino-5), 78.4 (Ino-3), 78.2 (Glc-4), 76.1 (<u>C</u>H$_2$ Nap), 76.0 (<u>C</u>H$_2$ Nap), 75.8 (Ino-6), 75.4 (<u>C</u>H$_2$ Nap), 74.9 (<u>C</u>H$_2$ Nap), 73.5 (<u>C</u>H$_2$ Nap), 72.3 (<u>C</u>H$_2$ Nap), 70.9 (-<u>C</u>H$_2$- Allyl), 70.4 (Glc-5), 67.6 (Glc-6), 65.6 (Ino-2), 63.5 (Glc-2), 34.5 (O-CO<u>C</u>H$_2$-CH$_2$- palmitoyl), 32.0, 29.85, 29.80, 29.7, 29.6, 29.5, 29.2 (<u>C</u>H$_2$ palmitoyl), 25.4 (O-COCH$_2$-<u>C</u>H$_2$- palmitoyl), 22.8 (<u>C</u>H$_2$ palmitoyl), 14.2 (<u>C</u>H$_3$ palmitoyl).

**HRMS:** [M+Na]$^+$ cald 1508.749032; found 1508.7793.

**2-Azido-2-deoxy-3,4,6-tri-*O*-(2-naphthyl)methyl-α-D-glucopyranosyl-(1→6)-3,4,5-tri-*O*-(2-naphthyl)methyl-2-*O*-(palmitoyl)-D-*myo*-inositol (18)**

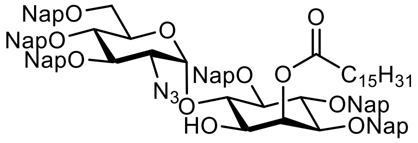


To a solution of *pseudo*-disaccharide **7** (23.6 mg, 0.0159 mmol) in a 1:1 DCM/MeOH anhydrous mixture (0.5 mL), PdCl$_2$ (2.6 mg, 0.0147 mmol) was added at room temperature. After 2.5 h, the mixture was filtered through a pad of Celite, and the volatiles were evaporated under reduced pressure. The resulting oil was purified by flash silica gel column chromatography afford compound **18** in 72% yield (0.0166 mg, 0.0114 mmol) as a white solid; R$_f$ = 0.30 (hexane/EtOAc, 7:3).

$[\alpha]_D^{20}$**:** 34.8 (c = 1, CHCl$_3$).

**IR (ATR) ν(cm$^{-1}$)** 3460 (H-O), 3056, 2926, 2855, 2109 (N=N=N), 1738 (C=O), 1603, 1510, 1467, 1367, 1250, 1125, 1054, 953, 894, 855, 817, 754.

**$^{1}$H NMR (400 MHz, CDCl$_3$) δ (ppm)** 7.85 – 7.63 (m, 19H, Ar), 7.60 – 7.55 (m, 4H, Ar), 7.40 – 7.50 (m, 15H, Ar), 7.34 – 7.39 (m, 2H, Ar), 7.28 (s, 1H, Ar), 7.22 (s, 1H, Ar), 7.10 (dd, *J* = 8.4, 1.7 Hz, 1H, Ar), 6.95 (dd, *J* = 8.4, 1.7 Hz, 1H, Ar), 5.88 (app t, *J* = 2.8 Hz, 1H, Ino-2), 5.36 (d, *J*= 3.6 Hz, 1H, Glc-1), 5.30 & 4.85 (ABq, *J* = 11.4 Hz, 2H, C$\underline{H}_2$ Nap), 5.16 & 4.95 (ABq, *J* =

10.8 Hz, 2H, C$\underline{H}_2$ Nap), 5.09 (ABq, *J* = 11.0 Hz, 2H, C$\underline{H}_2$ Nap), 4.98 & 4.72 (ABq, *J* = 11.3 Hz, 2H, C$\underline{H}_2$ Nap), 4.78 & 4.37 (ABq, *J* = 11.0 Hz, 2H, C$\underline{H}_2$ Nap), 4.45 & 3.89 (ABq, *J* = 12.2 Hz, 2H, C$\underline{H}_2$ Nap), 4.07 (td, *J* = 9.5, 3.0 Hz, 2H, Glc-3, Ino-4), 3.93 – 4.01 (m, 3H, Glc-5, Ino-6), 3.82 (t, *J* = 9.5 Hz, 2H, Ino-1, Glc-4), 3.67 – 3.72 (m, 2H, Glc-2, Ino-3), 3.53 (t, *J* = 9.4 Hz, 1H, Ino-5), 3.14 (dd, *J* = 11.0, 2.2 Hz, 1H, Glc-6a), 2.90 (dd, *J* = 11.1, 2.1 Hz, 1H, Glc-6b), 2.51 (t, *J* = 7.4 Hz, 2H, O-COC$\underline{H}_2$-CH$_2$- palmitoyl), 1.69 – 1.78 (m, 2H, O-COCH$_2$-C$\underline{H}_2$- palmitoyl), 1.35 – 1.45 (m, 2H, C$\underline{H}_2$ palmitoyl), 1.19 – 1.32 (m, 26H, C$\underline{H}_2$ palmitoyl), 0.90 (t, *J* = 6.7 Hz, 3H, C$\underline{H}_3$ palmitoyl).

**$^{13}$C NMR (101 MHz, CDCl$_3$) δ (ppm)** 173.6 (C=O), 136.1, 135.9, 135.4, 135.3, 135.1, 135.0, 133.39, 133.35, 133.3, 133.2, 133.1, 133.03, 132.99, 132.9 (C$_q$ Ar), 128.3, 128.22, 128.15, 128.1, 128.04, 128.02, 127.98, 127.9, 127.8, 127.73, 127.70, 127.3, 127.0, 126.8, 126.5, 126.3, 126.21, 126.18, 126.12, 126.07, 125.97, 125.95, 125.9, 125.6, 125.5 (C Ar), 99.6 (Glc-1), 82.2 (Ino-4, Ino-6), 81.2 (Glc-3), 80.8 (Ino-5), 78.5 (Ino-3), 78.1 (Glc-4), 76.1 ($\underline{C}$H$_2$ Nap), 75.7 ($\underline{C}$H$_2$ Nap), 75.5 ($\underline{C}$H$_2$ Nap), 74.9 ($\underline{C}$H$_2$ Nap), 73.4 ($\underline{C}$H$_2$ Nap), 72.2 ($\underline{C}$H$_2$ Nap), 71.43 (Ino-1), 71.40 (Glc-5), 68.9 (Ino-2), 67.2 (Glc-6), 64.6 (Glc-2), 34.7 (O-CO$\underline{C}$H$_2$-CH$_2$- palmitoyl), 32.1, 29.85, 29.80, 29.8, 29.7, 29.6, 29.5, 29.2 ($\underline{C}$H$_2$ palmitoyl), 25.5 (O-COCH$_2$-$\underline{C}$H$_2$-palmitoyl), 22.8 ($\underline{C}$H$_2$ palmitoyl), 14.3 ($\underline{C}$H$_3$ palmitoyl).

**HRMS:** [M+Na]$^+$ cald 1468.7177; found 1469.7498.

**2-Azido-2-deoxy-3,4,6-tri-*O*-(2-naphthyl)methyl-α-D-glucopyranosyl-(1→6)-3,4,5-tri-*O*-(2-naphthyl)methyl-1-*O*-(2-oleoyl-1-stearoyl-sn-glycero-3-phosphonate)-2-*O*-(palmitoyl)-D-*myo*-inositol (19)**

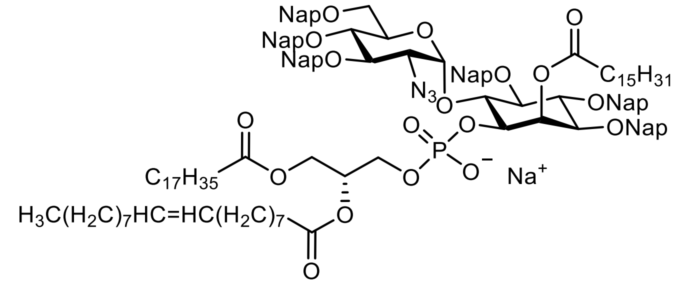


Alcohol **18** (70.4 mg, 0.0487 mmol) and H-phosphonate **9**[2] (87% purity, 70.5 mg, 0.0778 mmol) were co-evaporated with anhydrous pyridine (3 x 2 mL) and placed under high vacuum for 1 h. The residue was dissolved in anhydrous pyridine (2.4 mL) and PivCl (15.5 µL, 0.126 mmol) was added. The reaction mixture was stirred for 1.5 h at room temperature before water (217 µL) and iodine (37.0 mg, 0.146 mmol) were added. The reaction mixture was stirred for additional 1.5 h, and solid $Na_2S_2O_3$ was added until the color of the reaction changed from orange to yellow. Volatiles were removed under reduced pressure. The remaining was triturated with DCM to remove $Na_2S_2O_3$. Purification was performed by flash silica gel column chromatography. The obtained compound was dissolved in chloroform, and Amberlite IR 120 (Na$^+$) form resin was added; after stirring for 8 min, the solution was filtered and concentrated. The compound was further purified by LH20 size exclusion chromatography eluting with MeOH/CHCl$_3$ 1:2 to afford the protected glycolipid **19** in 82% yield (86.5 mg, 0.0402 mmol) as a white solid; R$_f$ = 0.50 (MeOH/DCM, 1:10).

**$^1$H NMR (400 MHz, CDCl$_3$) δ (ppm)** 7.57 – 7.77 (m, 18H, Ar), 7.29 – 7.50 (m, 23H, Ar), 7.13 – 7.22 (m, 3H, Ar), 6.84 (dd, *J* = 8.4, 1.6 Hz, 1H, Ar), 6.03 (s, 1H, Ino-2), 5.50 (s, 1H, Glc-1), 5.29 – 5.39 (m, 3H, -C$\underline{H}$=C$\underline{H}$- Oleoyl, C$\underline{H}$ sn-2), 5.20 (d, *J* = 11.1 Hz, 1H, C$\underline{H}_2$ Nap), 5.10 (d,

J = 10.9 Hz, 1H, C<u>H</u>$_2$ Nap), 5.04 (s, 2H, C<u>H</u>$_2$ Nap), 4.94 & 4.66 (ABq, J = 11.0 Hz, 2H, C<u>H</u>$_2$ Nap), 4.87 – 4.97 (m, 2H, C<u>H</u>$_2$ Nap), 4.73 & 4.30 (d, J = 11.3 Hz, 2H, C<u>H</u>$_2$ Nap), 4.58 & 4.16 (ABq, J = 12.0 Hz, 2H, C<u>H</u>$_2$ Nap), 4.56 (s, 1H, Ino-1), 4.39 (dd, J = 11.9, 3.9 Hz, 1H, C<u>H</u>$_{2a}$ sn-1), 4.15 – 4.34 (m, 5H, C<u>H</u>$_{2b}$ sn-1, C<u>H</u>$_2$ sn-3, Glc-5, Ino-6), 4.08 (t, J = 9.6 Hz 1H, Glc-3), 3.99 (t, J = 9.5 Hz, 1H, Ino-4), 3.75 – 3.83 (m, 2H, Ino-3, Glc-4), 3.52 – 3.63 (m, 2H, Glc-2, Ino-5), 3.32 (d, J = 10.9 Hz, 1H, Glc-6a), 3.15 (d, J = 11.1 Hz, 1H, Glc-6b), 2.46 (t, J = 7.4 Hz, 2H, O-COC<u>H</u>$_2$-CH$_2$- palmitoyl), 2.26 – 2.39 (m, 5H, 2 x O-COC<u>H</u>$_2$-CH$_2$- lipid), 1.94 – 2.02 (m, 4H, -C<u>H</u>$_2$-CH=CH-C<u>H</u>$_2$- Oleoyl), 1.54 – 1.69 (m, 6H, 3 x O-COCH$_2$-C<u>H</u>$_2$-), 1.24 (d, J = 11.9 Hz, 97H, C<u>H</u>$_2$ lipid), 0.83 – 0.91 (m, 14H, 3 x C<u>H</u>$_3$ lipid).

**$^{13}$C NMR (101 MHz, CDCl$_3$) δ (ppm)** 173.4 (2 x <u>C</u>=O), 173.1 (<u>C</u>=O), 135.8, 135.6, 135.5, 135.3, 135.2, 135.0, 133.34, 133.31, 133.25, 133.18, 133.14, 133.10, 133.02, 133.00, 132.90, 132.87 (C$_q$ Ar), 130.1 (-<u>C</u>H=CH- Oleoyl), 129.9 (-CH=<u>C</u>H- Oleoyl), 128.3, 128.24, 128.20, 128.16, 128.12, 128.08, 128.03, 128.00, 127.96, 127.9, 127.8, 127.72, 127.70, 127.4, 126.9, 126.8, 126.6, 126.5, 126.3, 126.18, 126.15, 126.1, 126.05, 126.02, 125.99, 125.9, 125.8, 125.7 (C Ar), 97.7 (Glc-1), 81.8 (Ino-4), 80.4 (Ino-5), 80.2 (Glc-3), 78.3 (Ino-3), 78.2 (Glc-4), 77.2 (Ino-1), 76.0 (2 x <u>C</u>H$_2$ Nap), 75.4 (Ino-6), 74.9 (<u>C</u>H$_2$ Nap), 73.5 (2 x <u>C</u>H$_2$ Nap), 72.5 (<u>C</u>H$_2$ Nap), 70.9 (Glc-5), 69.5 (<u>C</u>H sn-2), 68.5 (Ino-2), 67.6 (Glc-6), 65.8 (<u>C</u>H$_2$ sn-3), 63.6 (Glc-2), 61.9 (<u>C</u>H$_2$ sn-1), 34.6 (O-CO<u>C</u>H$_2$-CH$_2$- palmitoyl), 34.3 (O-CO<u>C</u>H$_2$-CH$_2$- lipid), 34.2 (O-CO<u>C</u>H$_2$-CH$_2$- lipid), 32.1, 32.0, 29.9, 29.9, 29.82, 29.77, 29.70, 29.68, 29.6, 29.52, 29.50, 29.46, 29.44, 29.40, 29.35, 29.33, 29.27, 29.2 (<u>C</u>H$_2$ lipid), 27.4 (-<u>C</u>H$_2$-CH=CH-<u>C</u>H$_2$- Oleoyl), 25.5 (O-COCH$_2$-<u>C</u>H$_2$- palmitoyl), 25.0 (2 x O-COCH$_2$-<u>C</u>H$_2$- lipid), 22.84, 22.83 (<u>C</u>H$_2$ lipid), 14.3 (3 x <u>C</u>H$_3$ lipid).

**$^{31}$P NMR (162 MHz, CDCl$_3$) δ (ppm) -**0.82.

**HRMS:** [M-H]$^-$ cald 2129.2295; found 2129.2083.

**2-Amino-2-deoxy-α-D-glucopyranosyl-(1→6)-1-*O*-(2-oleoyl-1-stearoyl-sn-glycero-3-phosphonate)- 2-*O*-(palmitoyl)-D-*myo*-inositol (4)**

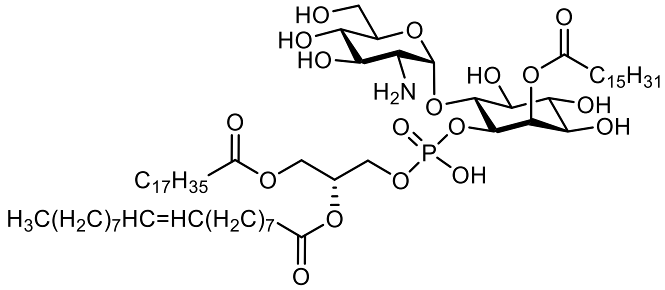


DDQ (60.7 mg, 0.267 mmol) was added to a solution of the protected glycolipid **19** (64 mg, 0.0297 mmol) in a 3:1 DCM/MeOH mixture (1.5 mL). The reaction mixture was stirred at room temperature for 5 h before volatiles were removed under reduced pressure. The residue was purified by LH20 size exclusion chromatography eluting with MeOH/CHCl$_3$ 9:1. To the obtained deprotected glycolipid (29 mg, 0.0225 mmol), 0.45 mL of a 1 M solution of P(CH$_3$)$_3$ in THF were added followed by water (82 µL). After 2h, the reaction was concentrated under vacuum, and the crude was purified by LH20 size exclusion chromatography eluting with MeOH/CHCl$_3$/H$_2$O 3:3:1 followed by trituration with MeOH to give the final GPI-fragment **4** in 48% yield (18.2 mg, 0.0144 mmol) as a slightly yellow solid.

**$^1$H NMR (400 MHz, CDCl$_3$) δ (ppm)** 5.13 – 5.25 (m, 2H, Glc-1, Ino-2), 4.96 – 5.06 (m, 2H, C<u>H</u>=C<u>H</u>- Oleoyl), 4.84 – 4.94 (m, 1H, C<u>H</u> sn-2), 3.96 – 4.11 (m, 2H, C<u>H</u>$_{2a}$ sn-1, Ino-1), 3.75 –

3.88 (m, 2H, C<u>H</u>$_{2b}$ sn-1, Glc-5), 3.45 – 3.68 (m, 5H, C<u>H</u>$_2$ sn-3, Glc-3, Glc-6a, Ino-6), 3.37 – 3.45 (m, 1H, Glc-6b), 3.20 – 3.35 (m, 2H, Ino-3), 2.99 – 3.15 (m, 2H, Glc-4), 2.78 – 2.88 (m, 1H, Glc-2), 2.04 – 2.12 (m, 2H, O-COC<u>H</u>$_2$-CH$_2$- palmitoyl), 1.94 – 2.03 (m, 4H, 2 x O-COC<u>H</u>$_2$-CH$_2$- lipid), 1.62 – 1.74 (m, 4H, -C<u>H</u>$_2$-CH=CH-C<u>H</u>$_2$- Oleoyl), 1.21 – 1.33 (m, 7H, 3 x O-COCH$_2$-C<u>H</u>$_2$-), 0.84 – 1.04 (m, 82H, C<u>H</u>$_2$ lipid), 0.54 (t, *J* = 6.6 Hz, 10H, 3 x C<u>H</u>$_3$ lipid).

**$^{13}$C NMR (101 MHz, CDCl$_3$) δ (ppm)** 173.7 (<u>C</u>=O), 173.4 (2 x <u>C</u>=O), 129.6 (-<u>C</u>H=CH- Oleoyl), 129.3 (-<u>C</u>H=CH- Oleoyl), 94.8 (Glc-1), 76.7 (Ino-6), 74.3 (Ino-1), 72.68 (Ino-2), 72.67, 72.6, 71.9 (Glc-5), 69.9 (<u>C</u>H sn-2, Glc-3), 69.4 (Glc-4), 69.1, 63.6 (<u>C</u>H$_2$ sn-3), 62.4 (<u>C</u>H$_2$ sn-1), 60.4 (Glc-6), 53.7 (Glc-2), 33.8 (O-CO<u>C</u>H$_2$-CH$_2$- palmitoyl), 33.7 (2 x O-CO<u>C</u>H$_2$-CH$_2$- lipid), 31.6, 29.41, 29.37, 29.32, 29.26, 29.1, 29.00, 28.97, 28.94, 28.91, 28.86 (<u>C</u>H$_2$ lipid), 26.8 (-<u>C</u>H$_2$-CH=CH-<u>C</u>H$_2$- Oleoyl), 24.6 (O-COCH$_2$-<u>C</u>H$_2$- palmitoyl), 24.5 (2 x O-COCH$_2$-<u>C</u>H$_2$- lipid), 22.3 (<u>C</u>H$_2$ lipid), 13.6 (3 x <u>C</u>H$_3$ lipid).

**$^{31}$P NMR (162 MHz, CDCl$_3$) δ (ppm) -**1.52.

**HRMS:** [M-H]$^-$ cald 1262.8634; found 1262.8473.

## Synthesis of Compound 5

**2-Azido-2-deoxy-4,6-*O*-((2-naphthyl)methylidene)-α-D-glucopyranosyl-(1→6)-1-*O*-allyl-2,3,4,5-tetra-*O*-(2-naphthyl)methyl-D-*myo*-inositol (20)**

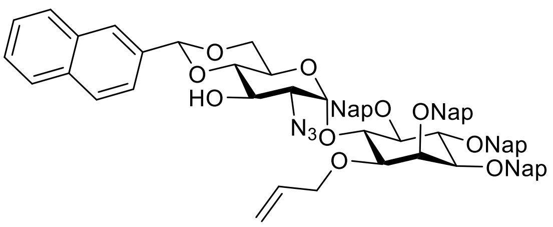


A solution of a 11/1 α:β mixture of *pseudo*-disaccharide **10**[1] (0.0629 mmol) and NaOMe (10 mg, 0.189 mmol) in anhydrous MeOH (2 mL) was stirred at 40 °C for 3 h. The reaction was neutralized with Amberlite IR 120 (H$^+$) resin, filtered and concentrated *in vacuo*. The resulting residue was purified by flash silica gel column chromatography to give the desired product in 94% yield over two steps (57.5 mg, 0.0594 mmol) as a white solid. The α and β anomers were separated during the column; R$_f$ α-anomer= 0.31, R$_f$ β-anomer= 0.2 (hexane/EtOAc, 3:7).

CSA (13.5 mg, 0.0580) was added to a mixture of the isolated α-anomer (225 mg, 0.232 mmol) and 2-(dimethoxymethyl)naphthalene[4] (80% purity, 176 mg, 0.696 mmol) in anhydrous ACN (6.4 mL). After stirring for 3 h, the reaction was quenched with aq. NaHCO$_3$, diluted with ethyl acetate and washed with aq. NaHCO$_3$. The aqueous phase was extracted with EtOAc. Organic layers were combined, washed with brine, dried over Na$_2$SO$_4$, filtered and concentrated under reduced pressure. The crude was purified by silica gel column chromatography to afford **20** in 91% yield (233 mg, 0.211 mmol) as a white solid; R$_f$ = 0.46 (hexane/EtOAc, 7:3).

$[\alpha]_D^{20}$**:** 51.48 (c = 1, CHCl$_3$).

**IR (ATR) ν(cm$^{-1}$)** 3500 (O-H), 3057, 2867, 2109 (N=N=N), 1603, 1510, 1366, 1271, 1127, 1088, 856, 818, 745.

**$^1$H NMR (400 MHz, CDCl$_3$) δ (ppm)** 7.76 – 7.91 (m, 11H, Ar), 7.59 – 7.71 (m, 9H, Ar), 7.43 – 7.54 (m, 12H, Ar), 7.27 – 7.34 (m, 2H, Ar), 7.03 (dd, *J* = 8.5, 1.7 Hz, 1H, Ar), 5.95 – 6.05 (m, 1H, -C<u>H</u>= Allyl), 5.86 (d, *J* = 3.8 Hz, 1H, Glc-1), 5.53 (s, 1H, C<u>H</u> Naphtylidene acetal), 5.28 (dd, *J* = 17.2, *J* = 1.7 Hz, 1H, =C$\underline{H}_{2a}$ Allyl), 5.16 – 5.24 (m, 4H, C$\underline{H}_2$ Nap, =C$\underline{H}_{2b}$ Allyl), 5.12 & 5.09 (ABq, *J* = 12.3 Hz, 2H, C$\underline{H}_2$ Nap), 4.98 (d, *J* = 10.8 Hz, 1H, C$\underline{H}_2$ Nap), 4.90 & 4.84 (ABq, *J* = 11.9 Hz, 2H, C$\underline{H}_2$ Nap), 4.46 (t, *J* = 9.6 Hz, 1H, Ino-6), 4.30 – 4.39 (m, 2H, Ino-4, Glc-5), 4.22 – 4.30 (m, 2H, Glc-3, Glc-6a), 4.18 (app t, *J* = 2.3 Hz, 1H, Ino-2), 4.01 – 4.12 (m, 2H, -C$\underline{H}_2$- Allyl), 3.60 – 3.67 (m, 2H, Glc-6b, Ino-5), 3.57 (dd, *J* = 9.8, 2.3 Hz, 1H, Ino-3), 3.46 – 3.53 (m, 2H, Ino-1, Glc-4), 3.26 (dd, *J*= 10.1, 3.8 Hz, 1H, Glc-2), 2.70 (br s, 1H, HO-Glc-3).

**$^{13}$C NMR (101 MHz, CDCl$_3$) δ (ppm)** 136.3, 136.1, 136.0, 135.8 (C$_q$ Ar), 134.3 (-<u>C</u>H= Allyl), 133.7, 133.5, 133.33, 133.31, 133.29, 133.12, 133.07, 132.94, 132.92, 132.8 (C$_q$ Ar), 128.4, 128.3, 128.19, 128.17, 128.14, 128.1, 128.03, 128.01, 127.84, 127.82, 127.71, 127.70, 127.7, 126.73, 126.71, 126.5, 126.43, 126.39, 126.3, 126.18, 126.16, 126.10, 126.08, 126.0, 125.93, 125.88, 125.86, 125.84, 125.80, 125.6, 125.3, 123.8 (C Ar). 117.5 (=<u>C</u>H$_2$ Allyl), 102.0 (<u>C</u>H Naphtylidene acetal), 98.2 (Glc-1), 82.1 (Ino-1), 82.06 (Ino-4), 82.02 (Glc-4), 81.6 (Ino-5), 80.8 (Ino-3), 76.0 (<u>C</u>H$_2$ Nap), 75.6 (<u>C</u>H$_2$ Nap), 75.4 (Ino-6), 74.3 (<u>C</u>H$_2$ Nap), 73.2 (<u>C</u>H$_2$ Nap), 73.0 (Ino-2), 71.1 (-<u>C</u>H$_2$- Allyl), 68.9 (Glc-6), 68.8 (Glc-3), 63.3 (Glc-2), 62.4 (Glc-5).

**HRMS:** [M+Na]$^+$ cald 1128.4411; found 1128.4623. [M+K]$^+$ cald 1144.4150; found 1144.4374.

**ESI-MS:** 1129.3 [M-Na]$^+$.

**2-Azido-2-deoxy-3-*O*-(2-naphthyl)methyl-4,6-*O*-((2-naphthyl)methylidene)-α-D-glucopyranosyl-(1→6)-1-*O*-allyl-2,3,4,5-tetra-*O*-(2-naphthyl)methyl-D-*myo*-inositol (21)**

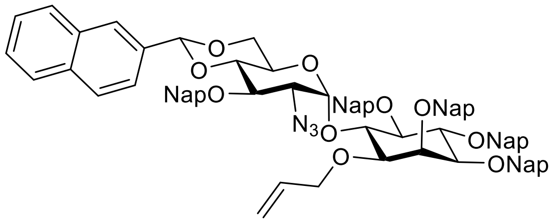


To a mixture of alcohol **20** (207 mg, 0.187 mmol) and TBAI (104 mg, 0.281 mmol) in DMF (2.6 mL), NaH (15.0 mg, 0.0374 mmol, 60% in mineral oil) was added at 0 °C. After 40 min, NapBr (62 mg, 0.281 mmol) was added and the mixture was stirred for additional 2h. The reaction was quenched with water, and volatiles were removed under reduced pressure. The remaining was dissolved with ethyl acetate, and the solution was extracted with water. The layers were separated, and the aqueous phase was extracted with ethyl acetate. Organic layers were washed with brine, dried with $Na_2SO_4$, filtered, and evaporated. The crude was purified by flash silica gel column chromatography to afford the fully protected *pseudo*-disaccharide **21** in 98% yield (229.8 mg, 0.184 mmol) as a white solid; $R_f$ = 0.28 (hexane/EtOAc, 5:1).

$[\alpha]_D^{20}$**:** 40.91 (c = 1, CHCl$_3$).

**IR (ATR) ν(cm$^{-1}$)** 3057, 2863, 2107 (N=N=N), 1603, 1510, 1465, 1366, 1272, 1172, 1125, 1089, 1036, 893, 856, 817, 750.

**$^1$H NMR (400 MHz, CDCl$_3$) δ (ppm)** 7.75 – 7.91 (m, 15H, Ar), 7.60 – 7.75 (m, 9H, Ar), 7.41 – 7.60 (m, 18H, Ar), 5.93 – 6.07 (m, 1H, -C<u>H</u>= Allyl), 5.85 (d, *J* = 3.8 Hz, 1H, Glc-1), 5.65 (s, 1H,

CH Naphtylidene acetal), 5.28 (dd, *J* = 17.2, 1.6 Hz, 1H, =C$H_{2a}$ Allyl), 5.16 – 5.25 (m, 2H, =C$H_{2b}$ Allyl, C$H_2$ Nap), 5.07 – 5.15 (m, 5H, C$H_2$ Nap), 4.95 – 5.01 (m, 2H, C$H_2$ Nap), 4.89 & 4.83 (ABq, *J* = 11.9 Hz, 2H, C$H_2$ Nap), 4.30 – 4.48 (m, 3H, Ino-4, Ino-6, Glc-5), 4.28 (dd, *J* = 10.2, 4.8 Hz, 1H, Glc-6a), 4.17 (s, 1H, Ino-2), 4.10 – 4.15 (m, 1H, Glc-3), 4.07 (app t, *J* = 6.5 Hz, 2H, -C$H_2$- Allyl), 3.77 (t, *J* = 9.4 Hz, 1H, Glc-4), 3.70 (t, *J* = 10.2 Hz, 1H, Glc-6b), 3.62 (t, *J* = 9.3 Hz, 1H, Ino-5), 3.56 (d, *J* = 7.8 Hz, 1H, Ino-3), 3.47 (d, *J* = 9.6 Hz, 1H, Ino-1), 3.43 (dd, *J* = 10.0, 3.8 Hz, 1H, Glc-2).

**$^{13}$C NMR (101 MHz, $CDCl_3$) δ (ppm)** 136.3, 136.2, 135.9, 135.8, 135.5, 134.9 ($C_q$ Ar), 134.4 (-CH= Allyl), 133.6, 133.4, 133.3, 133.3, 133.11, 133.07, 133.0, 132.91, 132.90 ($C_q$ Ar), 128.5, 128.33, 128.27, 128.19, 128.18, 128.15, 128.1, 128.0, 127.9, 127.84, 127.81, 127.76, 127.73, 127.69, 127.6, 127.1, 126.7, 126.6, 126.5, 126.4, 126.3, 126.23, 126.15, 126.10, 126.07, 126.05, 125.96, 125.95, 125.9, 125.8, 125.8, 125.7, 125.6, 125.6, 124.0 (C Ar), 117.4 (=$CH_2$ Allyl), 101.5 (CH Naphtylidene acetal), 98.2 (Glc-1), 83.1 (Glc-4), 82.1 (Ino-4), 82.0 (Ino-1), 81.4 (Ino-5), 80.9 (Ino-3), 75.91 ($CH_2$ Nap), 75.90 (Glc-3), 75.6 ($CH_2$ Nap), 75.4 (Ino-6), 74.9 ($CH_2$ Nap), 74.3 ($CH_2$ Nap), 73.2 ($CH_2$ Nap), 73.0 (Ino-2), 71.2 (-$CH_2$- Allyl), 69.0 (Glc-6), 63.3 (Glc-2), 62.7 (Glc-5).

**HRMS:** $[M+Na]^+$ cald 1268.5037; found 1268.5302.

**ESI-MS:** 1269.3 $[M-Na]^+$.

**2-Azido-2-deoxy-3,6-di-*O*-(2-naphthyl)methyl-α-D-glucopyranosyl-(1→6)-1-*O*-allyl-2,3,4,5-tetra-*O*-(2-naphthyl)methyl-D-*myo*-inositol (22)**

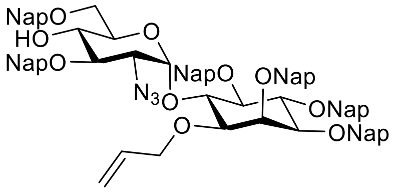


To a solution of the fully protected *pseudo*-disaccharide **21** (45.2 mg, 0.0363 mmol) in a 4:1 ACN/DCM anhydrous mixture (1.2 mL), $Me_2EtSiH$ (47.8 µL, 0.363 mmol) and $Cu(OTf)_2$ (3 mg, 8.29 µmol) were added at 0 °C. After 30 min, the reaction was quenched with aq. $NaHCO_3$, diluted with ethyl acetate and extracted with aq. $NaHCO_3$. The aqueous phase was extracted with EtOAc. Organic layers were combined, washed with brine, dried over $Na_2SO_4$, filtered and concentrated by rotatory evaporation. The resulting colorless oil was purified by silica gel column chromatography to give the desired *pseudo*-disaccharide **22** in 75% yield (33.8 mg, 0.0271 mmol) as a white solid; $R_f$ = 0.56 (hexane/EtOAc, 7:3). The corresponding diol was also obtained as a result of complete acetal opening with 23% yield (9.3 mg, 0.00839 mmol); $R_f$ = 0.17 (hexane/EtOAc, 7:3).

$[\alpha]_D^{20}$**:** 4.59 (c = 1, $CHCl_3$).

**IR (ATR) ν(cm$^{-1}$)** 3472 (O-H), 3055, 2926, 2108 (N=N=N), 1603, 1510, 1460, 1365, 1272, 1216, 1171.6, 1125, 1054, 953, 894, 856, 817, 751, 667.

**$^1$H NMR (400 MHz, $CDCl_3$) δ (ppm)** 7.77 – 7.91 (m, 16H, Ar), 7.58 – 7.72 (m, 10H, Ar), 7.45 – 7.51 (m, 10H, Ar), 7.31 – 7.40 (m, 5H, Ar), 7.17 (dd, *J* = 8.4, 1.7 Hz, 1H, Ar), 5.93 – 6.04 (m, 1H, -CH= Allyl), 5.82 (d, *J* = 3.7 Hz, 1H, Glc-1), 5.28 (dd, *J* = 17.2, 1.7 Hz, 1H, =C$H_{2a}$ Allyl), 5.16 – 5.26 (m, 3H, =C$H_{2b}$ Allyl, C$H_2$ Nap), 4.98 – 5.14 (m, 5H, C$H_2$ Nap), 4.91 (d, *J* = 11.5

Hz, 1H, C$\underline{H}_2$ Nap), 4.87 & 4.82 (ABq, $J$ = 11.9 Hz, 2H, C$\underline{H}_2$ Nap), 4.31 – 4.45 (m, 3H, Ino-4, Ino-6, C$\underline{H}_2$ Nap), 4.15 – 4.20 (m, 2H, Ino-2, C$\underline{H}_2$ Nap), 4.10 – 4.15 (m, 1H, Glc-5), 4.02 – 4.10 (m, 2H, -C$\underline{H}_2$- Allyl), 3.93 (dd, $J$ = 10.3, 8.7 Hz, 1H, Glc-3), 3.78 (td, $J$ = 9.3, 3.3 Hz, 1H, Glc-4), 3.54 – 3.63 (m, 2H, Ino-3, Ino-5), 3.47 (dd, $J$ = 9.7, 2.1 Hz, 1H, Ino-1), 3.30 – 3.39 (m, 2H, Glc-2, Glc-6a), 3.19 (dd, $J$ = 10.4, 3.5 Hz, 1H, Glc-6b), 2.19 (d, $J$ = 3.3 Hz, 1H, HO-Glc-4).

**$^{13}$C NMR (101 MHz, CDCl$_3$) δ (ppm)** 136.4, 136.2, 136.0, 135.79, 135.78, 135.3 (C$_q$ Ar), 134.4 (-$\underline{C}$H= Allyl), 133.4, 133.33, 133.29, 133.21, 133.17, 133.11, 133.06, 132.97, 132.95, 132.8 (C$_q$ Ar), 128.5, 128.3, 128.2, 128.13, 128.09, 128.06, 128.0, 127.9, 127.82, 127.79, 127.75, 128.0, 127.7, 127.0, 126.72, 126.66, 126.5, 126.4, 126.3, 126.21, 126.19, 126.14, 126.12, 126.09, 126.07, 126.06, 126.02, 125.97, 125.93, 125.88, 125.82, 125.77, 125.6, 125.5 (C Ar), 117.2 (=$\underline{C}$H$_2$ Allyl), 97.8 (Glc-1), 82.12 (Ino-4), 82.09 (Ino-1), 81.7 (Ino-5), 80.9 (Ino-3), 79.5 (Glc-3), 76.0 ($\underline{C}$H$_2$ Nap), 75.6 ($\underline{C}$H$_2$ Nap), 75.3 (Ino-6), 75.0 ($\underline{C}$H$_2$ Nap), 74.2 ($\underline{C}$H$_2$ Nap), 73.4 ($\underline{C}$H$_2$ Nap), 73.0 ($\underline{C}$H$_2$ Nap), 72.9 (Ino-2), 72.4 (Glc-4), 71.2 (-$\underline{C}$H$_2$- Allyl), 69.44 (Glc-5), 69.37 (Glc-6), 63.0 (Glc-2).

**HRMS:** [M+Na]$^+$ cald 1270.5194; found 1270.5620. [M+K]$^+$ cald 1286.4933; found 1286.5371.

**ESI-MS:** 1271.3 [M-Na]$^+$, 1286.3 [M-K]$^+$.

**2,3,4,6-Tetra-*O*-(2-naphthyl)methyl-α-D-mannopyranosyl-(1→4)-2-azido-2-deoxy-3,6-di-*O*-(2-naphthyl)methyl-α-D-glucopyranosyl-(1→6)-1-*O*-allyl-2,3,4,5-tetra-*O*-(2-naphthyl)methyl-D-*myo*-inositol (8)**

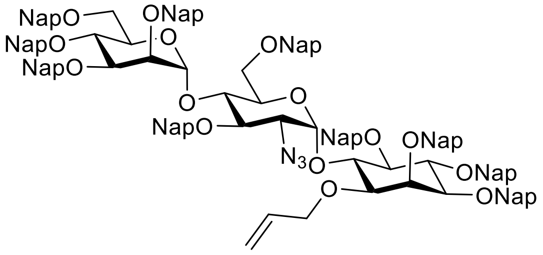


A solution of *pseudo*-disaccharide acceptor **22** (100 mg, 0.0801 mmol) and the trimannose glycosyl donor **23**[5] (59.1 mg, 0.120 mmol) in a 6:1 Et$_2$O/DCM anhydrous mixture (2 mL) was stirred at room temperature for 10 min. The mixture was cooled to 0 °C, and TMSOTf (4.3 µL, 24 µmol) was added. After stirring for 45 min, the reaction was quenched with Et$_3$N, and volatiles were removed under reduced pressure. The resulting crude was purified by flash silica gel column chromatography to give exclusively the α-anomer of the corresponding *pseudo*-trissacharide in 84% yield (0.106 mg, 0.0671 mmol) as a white solid; R$_f$ = 0.54 (hexane/EtOAc, 1:1). This compound was mixed with NaOMe (17.3 mg, 0.320 mmol) and 5 mL of a 4:1 MeOH/DCM anhydrous mixture. The reaction was stirred at room temperature for 2 h before it was neutralized with Amberlite IR 120 (H$^+$) resin, filtered and concentrated. The obtained triol was dissolved in anhydrous DMF (2 mL), and NaH (33.4 mg, 0.835 mmol, 60% in mineral oil) was added at 0 °C. After 40 min, NapBr (146 mg, 0.660 mmol) was added, and the mixture was stirred for two days before it was quenched with water. Volatiles were removed under reduced pressure. The remaining was dissolved with ethyl acetate, and the solution was extracted with water. The layers were separated, and the aqueous phase was extracted with ethyl acetate. Organic layers were washed with brine, dried with Na$_2$SO$_4$, filtered, and evaporated. The resulting oil was purified by flash silica gel column

chromatography to obtain **8** in 75% (98.7 mg, 0.0501 mmol) yield over two steps as a white solid; $R_f$ = 0.31 (hexane/EtOAc, 1:3).

$[\boldsymbol{\alpha}]_D^{20}$**:** 11.50 (c = 1, $CHCl_3$).

**IR (ATR) ν(cm$^{-1}$)** 3021, 3056, 2926, 2106 (N=N=N), 1699, 1635, 1603, 1510, 1462, 1366, 1346, 1266, 1216, 1125, 1096, 1053, 953, 894, 856, 816, 752, 668.

**$^1$H NMR (400 MHz, $CDCl_3$) δ (ppm)** 7.13 – 7.80 (m, 86H, Ar), 7.02 – 7.13 (m, 5H, Ar), 6.84 – 6.92 (m, 2H, Ar), 5.82 – 5.91 (m, 1H, -CH= Allyl), 5.80 (d, *J* = 3.6 Hz, 1H, Glc-1), 5.13 – 5.22 (m, 2H, =$CH_{2a}$ Allyl, Man-1), 5.08 (d, *J* = 10.5 Hz, 1H, =$CH_{2b}$ Allyl), 4.94 – 5.05 (m, 5H, $CH_2$ Nap), 4.91 (d, *J* = 10.8 Hz, 1H, $CH_2$ Nap), 4.69 – 4.82 (m, 4H, $CH_2$ Nap), 4.49 – 4.61 (m, 3H, $CH_2$ Nap), 4.31 – 4.46 (m, 3H, Ino-6, $CH_2$ Nap), 4.14 – 4.25 (m, 3H, Ino-4, $CH_2$ Nap), 4.05 – 4.15 (m, 4H, Ino-2, Man-4, Glc-5, $CH_2$ Nap), 3.88 – 4.00 (m, 3H, -$CH_2$- Allyl, $CH_2$ Nap), 3.81 – 3.88 (m, 2H, Glc-3, Glc-4), 3.73 (dd, *J* = 9.4, 2.7 Hz, 1H, Man-3), 3.60 (app t, *J* = 2.5 Hz, 1H, Man-2), 3.42 – 3.50 (m, 3H, Ino-3, Ino-5, Glc-6a), 3.31 – 3.41 (m, 2H, Ino-1, Man-6a), 3.16 – 3.26 (m, 3H, Glc-2, Glc-6b, Man-5), 3.01 (d, *J* = 10.8 Hz, 1H, Man-6b).

**$^{13}$C NMR (101 MHz, $CDCl_3$) δ (ppm)** 136.5, 136.3, 136.1, 136.11, 136.06, 136.03, 135.97, 135.8, 135.6 ($C_q$ Ar), 134.3 (-CH= Allyl), 133.38, 133.35, 133.32, 133.30, 133.28, 133.2, 133.13, 133.08, 133.06, 133.0, 132.9, 132.73, 132.69 ($C_q$ Ar), 128.6, 128.4, 128.21, 128.16, 128.12, 128.10, 128.07, 128.04, 128.01, 127.97, 127.9, 127.84, 127.79, 127.76, 127.74, 127.72, 127.67, 127.63, 127.60, 127.0, 126.8, 126.73, 126.71, 126.5, 126.43, 126.40, 126.36, 126.32, 126.29, 126.2, 126.1, 126.11, 126.07, 126.03, 125.99, 125.95, 125.91, 125.85, 125.81, 125.73, 125.68, 125.49, 125.46, 125.43, 125.40, 125.33, 125.29, 124.9, 124.7 (C Ar), 117.2 (=$CH_2$ Allyl), 101.2 (Man-1), 97.8 (Glc-1), 82.2 (Ino-4), 82.1 (Ino-1), 81.8 (Ino-5), 80.8 (Ino-3), 80.2 (Glc-3), 79.5 (Man-3), 78.2 (Glc-4), 76.8 (Man-2), 76.0 ($CH_2$ Nap), 75.5 (Ino-6), 75.4 ($CH_2$ Nap), 75.3 ($CH_2$ Nap), 74.6 (Man-4), 74.2 ($CH_2$ Nap), 74.0 ($CH_2$ Nap), 73.5 ($CH_2$ Nap), 73.4 ($CH_2$ Nap), 73.2 (Man-5), 73.0 ($CH_2$ Nap), 72.6 (Ino-2), 72.30 ($CH_2$ Nap), 72.26 ($CH_2$ Nap), 71.0 (-$CH_2$- Allyl), 70.0 (Glc-5), 69.0 (Glc-6), 68.6 (Man-6), 63.3 (Glc-2).

**HRMS:** $[M+Na]^+$ cald 1992.8226; found 1992.819.

**ESI-MS:** 1992.6 $[M-Na]^+$.

**2,3,4,6-Tetra-*O*-(2-naphthyl)methyl-α-D-mannopyranosyl-(1→4)-2-azido-2-deoxy-3,6-di-*O*-(2-naphthyl)methyl-α-D-glucopyranosyl-(1→6)-2,3,4,5-tetra-*O*-(2-naphthyl)methyl-D-*myo*-inositol (24)**

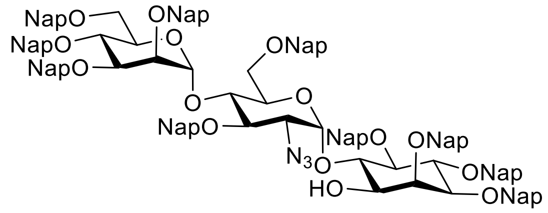


The fully protected *pseudo*-trisaccharide **8** (94 mg, 0.0477 mmol) was dissolved in a 47:53 MeOH/DCM anhydrous mixture, and $PdCl_2$ (4.7 mg, 0.0265 mmol) was added at room temperature. After 5.5 h, the mixture was filtered through a pad of Celite, and the volatiles were evaporated under reduced pressure. The resulting oil was purified by flash silica gel

column chromatography to obtain **24** in 83% yield (76.7 mg, 0.0397 mmol) as a white solid; $R_f$ = 0.60 (hexane/EtOAc, 13:7).

$[\alpha]_D^{20}$**:** 18.22 (c = 1, $CHCl_3$).

**IR (ATR) v(cm$^{-1}$)** 3471 (O-H), 3056, 2926, 2108 (N=N=N), 1699, 1603, 1510, 1462, 1367, 1344, 1272, 1171, 1125, 1097, 1049, 954, 893, 856, 816, 752.

**$^1$H NMR (400 MHz, $CDCl_3$) δ (ppm)** 7.75 – 7.85 (m, 13H, Ar), 7.61 – 7.70 (m, 16H, Ar), 7.53 – 7.58 (m, 10H, Ar), 7.45 – 7.51 (m, 11H, Ar), 7.38 – 7.44 (m, 12H, Ar), 7.28 – 7.36 (m, 12H, Ar), 7.17 – 7.26 (m, 3H, Ar), 7.16 (s, 1H, Ar), 7.10 (t, *J* = 7.5 Hz, 1H, Ar), 6.98 (dd, *J* = 8.5, 1.7 Hz, 1H, Ar), 5.60 (d, *J* = 3.5 Hz, 1H, Glc-1), 5.33 (d, *J* = 2.1 Hz, 1H, Man-1), 5.20 (d, *J* = 11.8 Hz, 1H, C$\underline{H}_2$ Nap), 5.12 (app t, *J* = 11.1 Hz, 2H, C$\underline{H}_2$ Nap), 4.96 – 5.04 (m, 3H, C$\underline{H}_2$ Nap), 4.86 – 4.95 (m, 4H, C$\underline{H}_2$ Nap), 4.52 – 4.72 (m, 4H, C$\underline{H}_2$ Nap), 4.19 – 4.31 (m, 5H, C$\underline{H}_2$ Nap, Ino-4), 4.05 – 4.19 (m, 6H, C$\underline{H}_2$ Nap, Ino-2, Man-4, Glc-5, Ino-6), 3.87 – 3.97 (m, 3H, Glc-3, Man-3, Glc-4), 3.67 – 3.76 (m, 2H, Ino-1, Man-2), 3.63 (dd, *J* = 9.9, 2.3 Hz, 1H, Ino-3), 3.47 – 3.58 (m, 4H, Glc-2, Ino-5, Man-5, Man-6a), 3.40 (br s, 2H, Glc-6), 3.22 – 3.31 (m, 1H, Man-6b), 3.17 (d, *J* = 6.8 Hz, 1H, HO-Ino-1).

**$^{13}$C NMR (101 MHz, $CDCl_3$) δ (ppm)** 136.3, 136.11, 136.07, 136.0, 135.9, 135.6, 135.33, 133.34, 133.30, 133.27, 133.24, 133.17, 133.1, 133.04, 132.99, 132.95, 132.92, 132.81, 132.77 ($C_q$ Ar), 128.5, 128.45, 128.37, 128.2, 128.14, 128.10, 128.08, 128.05, 128.02, 127.99, 127.96, 127.88, 127.86, 127.80, 127.77, 127.7, 127.6, 127.1, 126.9, 126.7, 126.6, 126.5, 126.43, 126.37, 126.35, 126.3, 126.24, 126.16, 126.11, 126.06, 126.03, 126.01, 125.96, 125.9, 125.8, 125.5, 125.4, 125.3, 125.1, 124.7 (C Ar), 100.9 (Man-1), 98.2 (Glc-1), 81.9 (Ino-4), 81.6 (Ino-5), 80.9 (Ino-3), 80.8 (Man-3), 80.3 (Glc-3), 77.8 (Glc-4), 77.4 (C-Ino), 77.1 (C-Ino), 76.7 (Man-2), 76.0 ($\underline{C}H_2$ Nap), 75.4 ($\underline{C}H_2$ Nap), 75.3 ($\underline{C}H_2$ Nap), 75.1 ($\underline{C}H_2$ Nap), 74.7 (Man-4), 74.4 ($\underline{C}H_2$ Nap), 73.6 (Ino-1), 73.5 ($\underline{C}H_2$ Nap), 73.3 ($\underline{C}H_2$ Nap), 73.2 ($\underline{C}H_2$ Nap, Man-5), 72.4 ($\underline{C}H_2$ Nap x2), 70.8 (Glc-5), 68.9 (Man-6), 68.8 (Glc-6), 64.2 (Glc-2).

**HRMS:** $[M+Na]^+$ cald 1952.7913; found 1952.7926.

**ESI-MS:** 1952.6 $[M-Na]^+$.

**2,3,4,6-Tetra-*O*-(2-naphthyl)methyl-α-D-mannopyranosyl-(1→4)-2-azido-2-deoxy-3,6-di-*O*-(2-naphthyl)methyl-α-D-glucopyranosyl-(1→6)-2,3,4,5-tetra-*O*-(2-naphthyl)methyl-1-*O*-(2-oleoyl-1-stearoyl-sn-glycero-3-phosphonate)-D-*myo*-inositol (25)**

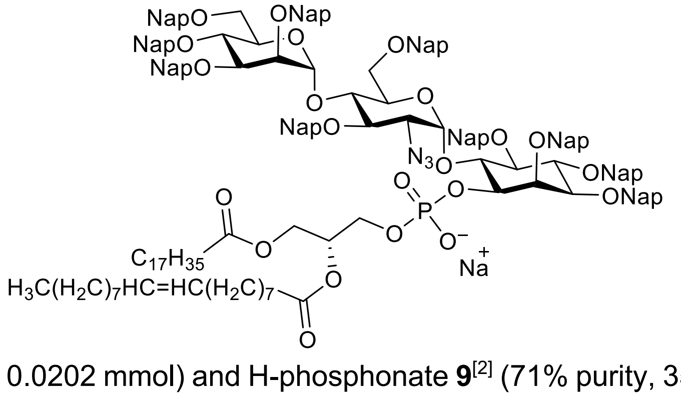

Alcohol **24** (39.1 mg, 0.0202 mmol) and H-phosphonate **9**[2] (71% purity, 35 mg, 0.0315 mmol) were co-evaporated with anhydrous pyridine (3 x 2 mL) and placed under high vacuum for 1.5

h. The residue was dissolved in anhydrous pyridine (2 mL) and PivCl (6.4 µL, 0.0525 mmol) was added. The reaction mixture was stirred for 1.5 h at room temperature before water (90 µL) and iodine (15.2 mg, 0.0600 mmol) were added. The reaction mixture was stirred for 2 h, and solid $Na_2S_2O_3$ was added until the orange color of the reaction disappeared. Volatiles were removed under reduced pressure. The remaining was triturated with DCM to remove $Na_2S_2O_3$. Purification was performed by flash silica gel column chromatography (eluting with MeOH/DCM 1:90 and gradually increasing the polarity to 1:10) to afford the lipidated *pseudo*-trisaccharide **25** in 82% yield (43.9 mg, 0.0162 mmol) as a white solid; $R_f$ = 0.68 (MeOH/DCM, 1:10).

$[\alpha]_D^{20}$**:** 30.1 (c = 1, $CHCl_3$).

**IR (ATR) ν(cm$^{-1}$)** 2626, 2855, 2110 (N=N=N), 1740 (C=O), 1603, 1510, 1466, 1366, 1259, 1125, 1050, 892, 856, 815, 753, 668.

**$^1$H NMR (400 MHz, $CDCl_3$) δ (ppm)** 7.51 – 7.86 (m, 36H, Ar), 7.27 – 7.48 (m, 29H, Ar), 7.08 – 7.21 (m, 4H, Ar), 6.95 (d, *J* = 8.0 Hz, 1H, Ar), 5.78 (d, *J*= 3.6 Hz, 1H, Glc-1), 5.23 – 5.37 (m, 4H, -C<u>H</u>=C<u>H</u>- Oleoyl, C<u>H</u> sn-2, Man-1), 4.89 – 5.20 (m, 9H, C$\underline{H}_2$ Nap), 4.72 – 4.88 (m, 3H, C$\underline{H}_2$ Nap, H-Ino), 4.50 – 4.66 (m, 6H, C$\underline{H}_2$ Nap), 4.39 – 4.47 (m, 3H), 4.09 – 4.36 (m, 10H, C$\underline{H}_2$ sn-1, C$\underline{H}_2$ sn-3, Ino-4), 3.89 – 4.06 (m, 3H, Glc-3, Glc-4), 3.71 – 3.83 (m, 2H, 2 x H-Ino), 3.55 – 3.68 (m, 3H, Man-2, Glc-6), 3.30 – 3.46 (m, 3H, Man-5, Glc-2, Man-6a), 3.17 (d, *J* = 10.6 Hz, 1H, Man-6b), 2.98 (d, *J* = 11.9 Hz, 1H, $Et_3N$ residue), 2.21 (q, *J* = 7.3 Hz, 4H, 2 x O-COC$\underline{H}_2$-$CH_2$- lipid), 1.91 – 2.03 (m, 4H, -C$\underline{H}_2$-CH=CH-C$\underline{H}_2$- Oleoyl), 1.45 – 1.56 (m, 4H, 2 x O-COC$H_2$-C$\underline{H}_2$- lipid), 1.14 – 1.34 (m, 63H, C$\underline{H}_2$ lipid), 0.85 – 0.92 (m, 6H, 2 x C$\underline{H}_3$ lipid).

**$^{13}$C NMR (101 MHz, $CDCl_3$) δ (ppm)** 173.6 (<u>C</u>=O), 173.2 (<u>C</u>=O), 137.5, 136.4, 136.2, 136.14, 136.08, 136.01, 135.7, 133.38, 133.36, 133.3, 133.2, 133.13, 133.07, 133.0, 132.9, 132.8, 132.7 ($C_q$ Ar), 130.1 (-<u>C</u>H=CH- Oleoyl), 129.9 (-CH=<u>C</u>H- Oleoyl), 128.8, 128.2, 128.11, 128.08, 128.04, 128.01, 127.97, 127.9, 127.8, 127.73, 127.69, 127.64, 127.59, 127.1, 126.8, 126.5, 126.4, 126.3, 126.2, 126.1, 126.05, 126.03, 125.99, 125.9, 125.8, 125.73, 125.68, 125.4, 125.35, 125.31, 125.2, 125.0 (C Ar), 101.4 (Man-1), 96.7 (Glc-1), 82.3 (Ino-4), 82.0, 80.9, 80.0 (Glc-3), 78.6 (Glc-4), 77.4, 77.0 (Man-2), 76.2, 76.0, 75.7, 75.4, 75.2, 75.0, 74.7, 74.2, 73.8, 73.5, 73.2 (Man-5), 72.5, 72.3, 70.8, 70.7, 69.9 (<u>C</u>H sn-2), 69.5, 69.1 (Glc-6, Man-6), 63.8 (Glc-2), 62.9 ($\underline{C}H_2$ sn-1), 34.4, 34.2, 32.1, 32.0, 29.90, 29.86, 29.8, 29.7, 29.5, 29.5, 29.4, 29.29, 29.27, 29.22, 28.18, 27.4, 27.3, 26.6, 25.0, 22.8, 14.3.

**$^{31}$P NMR (162 MHz, $CDCl_3$) δ (ppm)** -0.80.

**HRMS:** [M-H]$^-$ cald 2613.3031; found 2613.4924.

**α-D-Mannopyranosyl-(1→4)-2-amino-2-deoxy-α-D-glucopyranosyl-(1→6)-1-*O*-(2-oleoyl-1-stearoyl-sn-glycero-3-phosphonate)-D-*myo*-inositol (5)**

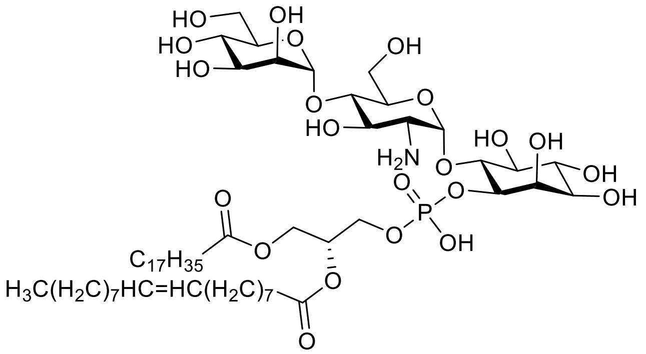

DDQ (70 mg, 0.308 mmol) was added to a solution of the protected glycolipid **25** (54.6 mg, 0.0207 mmol) in a 3:1 DCM/MeOH mixture (4 mL). The reaction mixture was stirred at room temperature for 6 h before volatiles were removed under reduced pressure. The residue was purified by LH20 size exclusion chromatography eluting with MeOH/$CHCl_3$ 9:1. To the obtained deprotected glycolipid, 0.79 mL of a 1M solution of $P(CH_3)_3$ in THF were added followed by water (160 µL). After 2h, the reaction was concentrated under vacuum, and the crude was purified by LH20 size exclusion chromatography eluting with THF followed by trituration with MeOH to give the final GPI-fragment **5** in 56% yield (13.8 mg, 0.0116 mmol) as a slightly yellow solid.

**$^1$H NMR (400 MHz, $CDCl_3$) δ (ppm)** 5.22 (d, *J*= 3.9 Hz, 1H, Glc-1), 5.02 – 5.10 (m, 2H, -C<u>H</u>=C<u>H</u>- Oleoyl), 4.90 – 5.01 (m, 2H, Man-1, C<u>H</u> sn-2), 4.09 – 4.16 (m, 1H, $C\underline{H}_{2a}$ sn-1), 3.82 – 3.95 (m, 3H, $C\underline{H}_{2b}$ sn-1), 3.67 – 3.79 (m, 4H, $C\underline{H_2}$ sn-3, Glc-3), 3.63 (t, *J* = 9.4 Hz, 1H), 3.46 – 3.58 (m, 3H), 3.28 – 3.45 (m, 7H), 3.15 (dd, *J* = 10.0, 2.7 Hz, 1H), 2.98 – 3.06 (m, 1H), 2.93 (dd, *J* = 10.6, 3.9 Hz, 1H, Glc-2), 1.98 – 2.09 (m, 4H, 2 x O-$COC\underline{H}_2$-$CH_2$- lipid), 1.69 – 1.76 (m, 4H, -$C\underline{H}_2$-CH=CH-$C\underline{H}_2$- Oleoyl), 1.24 – 1.35 (m, 4H, 2 x O-$COCH_2$-$C\underline{H}_2$- lipid), 0.91 – 1.07 (m, 53H, $C\underline{H}_2$ lipid), 0.59 (t, *J* = 6.7 Hz, 6H, 2 x $C\underline{H}_3$ lipid).

**$^{13}$C NMR (101 MHz, $CDCl_3$) δ (ppm)** 174.0 (<u>C</u>=O), 173.7 (<u>C</u>=O), 129.6 (-<u>C</u>H=CH- Oleoyl), 129.3 (-CH=<u>C</u>H- Oleoyl), 101.0 (Man-1), 95.0 (Glc-1), 76.3, 73.6, 73.0, 72.3, 71.4, 70.2 (<u>C</u>H sn-2, Glc-3), 66.5, 63.4 ($\underline{C}H_2$ sn-3), 62.5 ($\underline{C}H_2$ sn-1), 60.9, 53.9 (Glc-2), 33.8 (O-$CO\underline{C}H_2$-$CH_2$-lipid), 33.7 (O-$CO\underline{C}H_2$-$CH_2$- lipid), 31.5, 29.3, 29.23, 29.17, 29.1, 28.94, 28.91, 28.88, 28.7 ($\underline{C}H_2$ lipid), 26.8 (-$\underline{C}H_2$-CH=CH-$\underline{C}H_2$- Oleoyl), 24.6 (O-$COCH_2$-$\underline{C}H_2$- lipid), 24.5 (O-$COCH_2$-$\underline{C}H_2$- lipid), 22.2 ($\underline{C}H_2$ lipid), 13.5 (2 x $\underline{C}H_3$ lipid).

**$^{31}$P NMR (162 MHz, $CDCl_3$) δ (ppm)** 0.0023.

**HRMS:** $[M-H]^-$ cald 1186.6866; found 1186.6705.

## Cell Assays

**Generation of PIGA-, PIGL-, PIGW-, PIGM-KO HEK293 cells.** Knockout cells were generated by the CRISPR/Cas9 system. HEK293 cells were transfected with pX330-U6-Chimeric_BB-CBh-hSpCas9 containing each gRNA (AGCATTCTGCATGGCGATCG for PIGA, GACGGCTGGGAGCCGAAAGC and GCTTGGCCCGCCTAAGGCAC for PIGL, TGTTGTATCAAATATACCGA and AGAGATTATTCCTTCGCGGT for PIGW, TGTCCGTATACCTCACGTGC for PIGM). Two weeks later, cells were stained for CD59 expression and negative cells were sorted by FACS. Several clones of each gene knockout were selected by limiting dilution and sanger sequenced to confirm mutations.

**Compound treatment of cells.** Dried compounds were reconstituted with DMSO at a concentration of 10 mM (stock solution). Cells (1x$10^5$/well) were incubated in 12 well plates overnight. The medium was removed and 500 μL of serum free medium (D-MEM/Ham's F-12) containing the described concentrations of compounds was added. After 24 hours incubation, cells were analyzed by FACS. For further PIPLC treatment, compound treated cells were harvested and incubated in 50 μL of serum free medium containing 0.05 U/mL of PIPLC (Thermo Fisher Scientific) for 1.5 h at 37°C and were analyzed by FACS.

**Pulse-chase analysis of treated cells.** PIGA-KO and PIGL-KO cells were incubated in 24 well plates with 5x$10^4$ cells per well for overnight and pulsed with 250 μL of serum free medium containing 10 μM of compounds for 1h at 4°C. After washing, the cells were chased and analyzed by FACS. Percent restorations of CD59 expression to wild type cells were plotted in various time points.

**Flow cytometry analysis.** Cells were harvested and stained with anti-CD59 (5H8) or anti-DAF (IA10) monoclonal antibodies followed by second antibody, anti-mouse IgG-PE. Some cells were also stained with Alexa488 labeled inactive toxin aerolysin (FLAER) to detect GPI-APs. Stained cells were analyzed using a flow cytometer (MACSQuant VYB, Milteny Biotec).

**Rescue experiments using Streptolysin O.** Cells were incubated in HBSS with 130 μg/mL of streptolysin O (Bioacademia) on ice for 5 min and were washed with PBS. Then, cells were incubated with compounds or streptoavidin-alexa488 for a positive control in 250 μL of HBSS (20 μM final concentration for **4** and **5**, 2 μM for GlcNAcPI **1**, or 0.5 μg SA-Alexa488), for 15 min, at 37 °C. Following, 750 μL of serum free medium containing $CaCl_2$ were added to seal the holes made by streptolysin O. The cells were incubated for 24 h and were analyzed to determine CD59 expression by FACS.

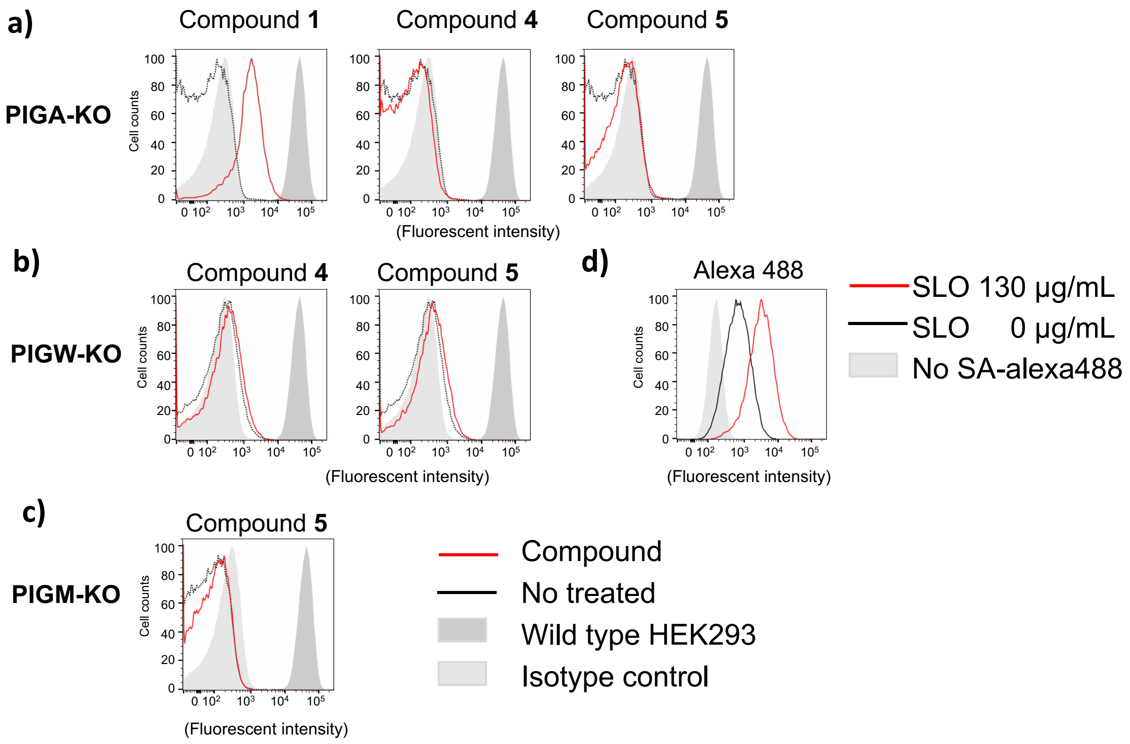


**Figure S1**. Determination of CD59 expression by flow cytometry after treatment of PIGA-KO, PIGW-KO and PIGM-KO HEK293 cells with Streptolysin O and compounds **1**, **4** and **5**. a) Restoration of CD59 on PIGA-KO cells after 24 h incubation with compounds **1, 4** and **5**. b) Restoration of CD59 on PIGW-KO cells after 24 h incubation with compounds **4** and **5**. c) Restoration of CD59 expression on

PIGM-KO cells after 24 h incubation with compound **5,** d) incorporation of SA-alexa488. These are the representative data of two independent experiments.

**Rescue experiments using Liposomes.** Egg PC (2.5 mg/mL) and compounds **1, 4**, or **5** were dissolved in a 200 µL of a chloroform: methanol (2:1) mixture. The samples were dried under a nitrogen stream to yield a thin film at the bottom of the glass tube. The lipid film was hydrated with 200 µL of PBS, vortexed and sonicated during 5 min to obtain small unilamellar vesicles (SUV). These liposomes containing (20 µM final concentration for **4** and **5**, 2 µM for GlcNAcPI **1**) were incubated with PIGA-KO, PIGW-KO and PIGM-KO cells in 500 µL of serum free medium for 24 h. The cells were analyzed by FACS.

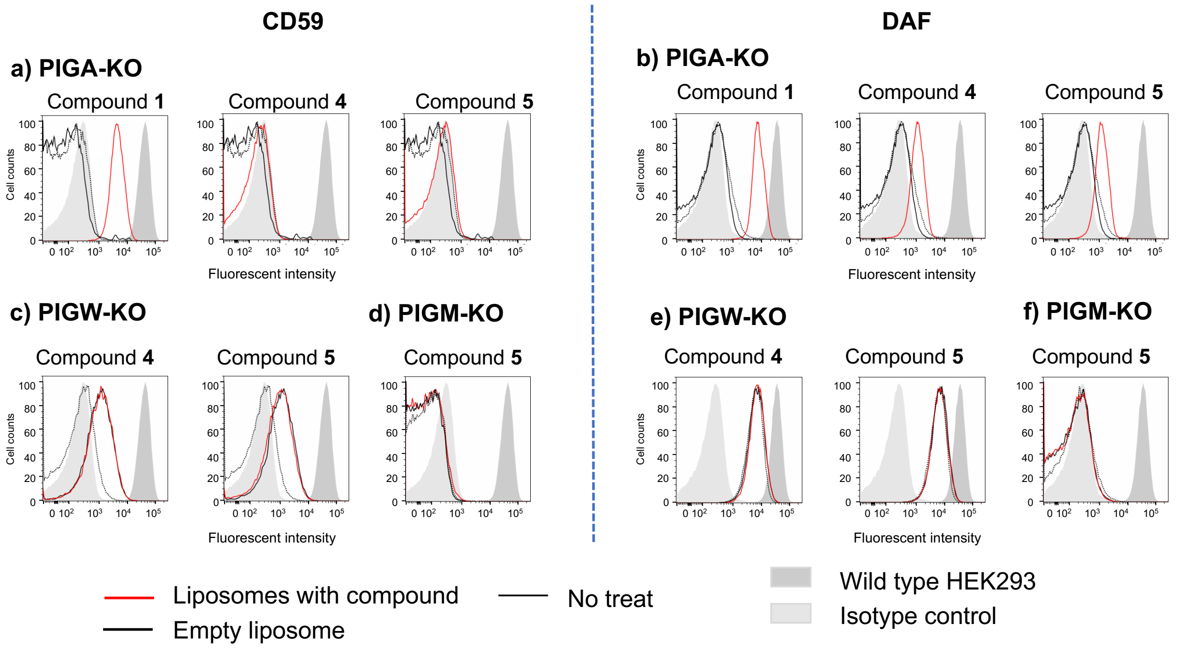


**Figure S2**. Determination of CD59 and DAF expression by flow cytometry after treatment of PIGA-KO, PIGW-KO and PIGM-KO HEK293 cells with liposomes containing compounds **1**, **4** and **5**. a) Restoration of CD59 expression on PIGA-KO cells after 24 h incubation with compounds **1, 4** and **5,** b) Restoration of DAF on PIGA-KO cells after 24 h incubation with compounds **1, 4** and **5,** c) Restoration of CD59 on PIGW-KO cells after 24 h incubation with compounds **4** and **5,** d) Restoration of CD59 expression on PIGM-KO cells after 24 h incubation with compound **5,** e) Restoration of DAF expression on PIGW-KO cells after 24 h incubation with compounds **4** and **5,** f) Restoration of DAF expression on PIGM-KO cells after 24 h incubation with compounds **5**. These are the representative data of two independent experiments.

## References


**[1]** Lee B. Y., Seeberger P. H., Varon Silva D., *Chem. Commun*. **2016**, 52, 1586-1589.

**[2]** Malik A., Seeberger P. H., Brezesinski G., Varon Silva D., *ChemPhysChem*. **2021**, 22, 757-763.

**[3]** Ding N., Li X., Chinoy Z., Boons G., *Org. Lett.* **2017**, 19, 14, 3827–3830.

**[4]** Ito S., Hayashi A., Komai H., Yamaguchi H., Kubota Y., Asami M., *Tetrahedron.* **2011**, 67, 2081-2089.

**[5]** Patel M., Vijayakrishnan B., Koeppe J., Chalker J., Doores K., Davis B., *Chem. Commun.*, **2010**, 46, 9119-9121.

## NMR Spectra

### $^{1}$H NMR spectrum of *pseudo*-disaccharide 6

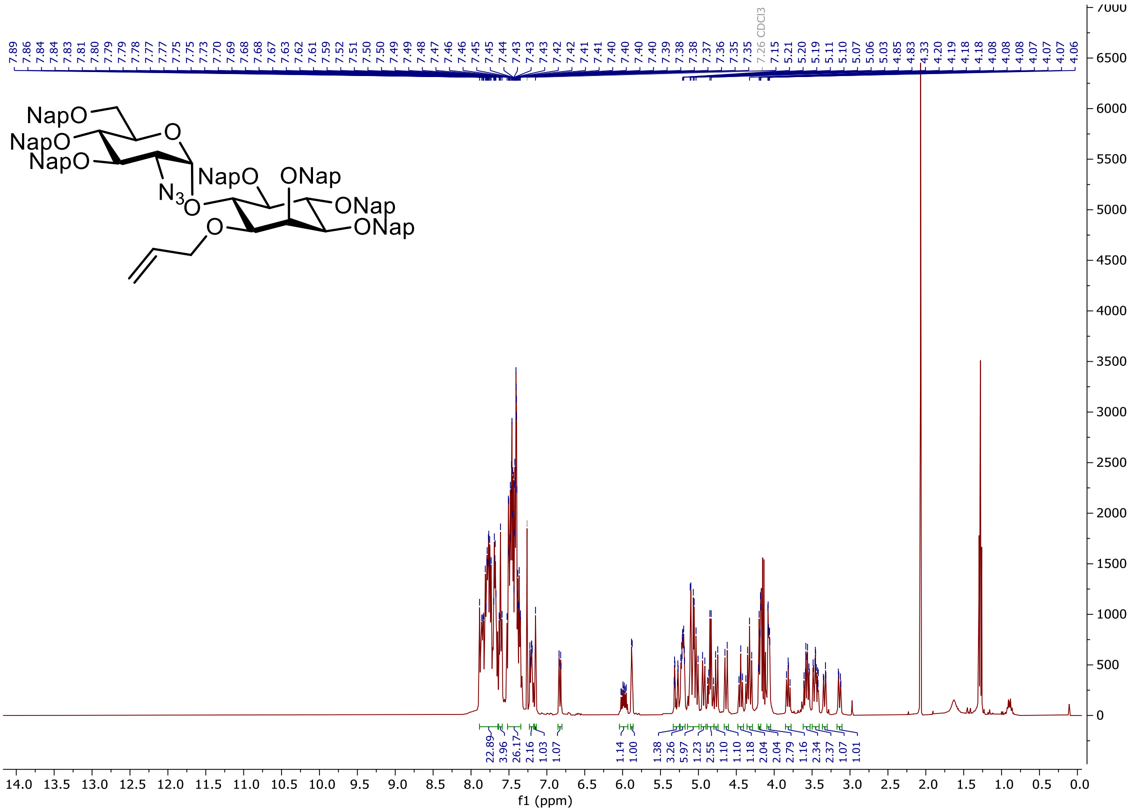

### $^{13}$C NMR spectrum of *pseudo*-disaccharide 6

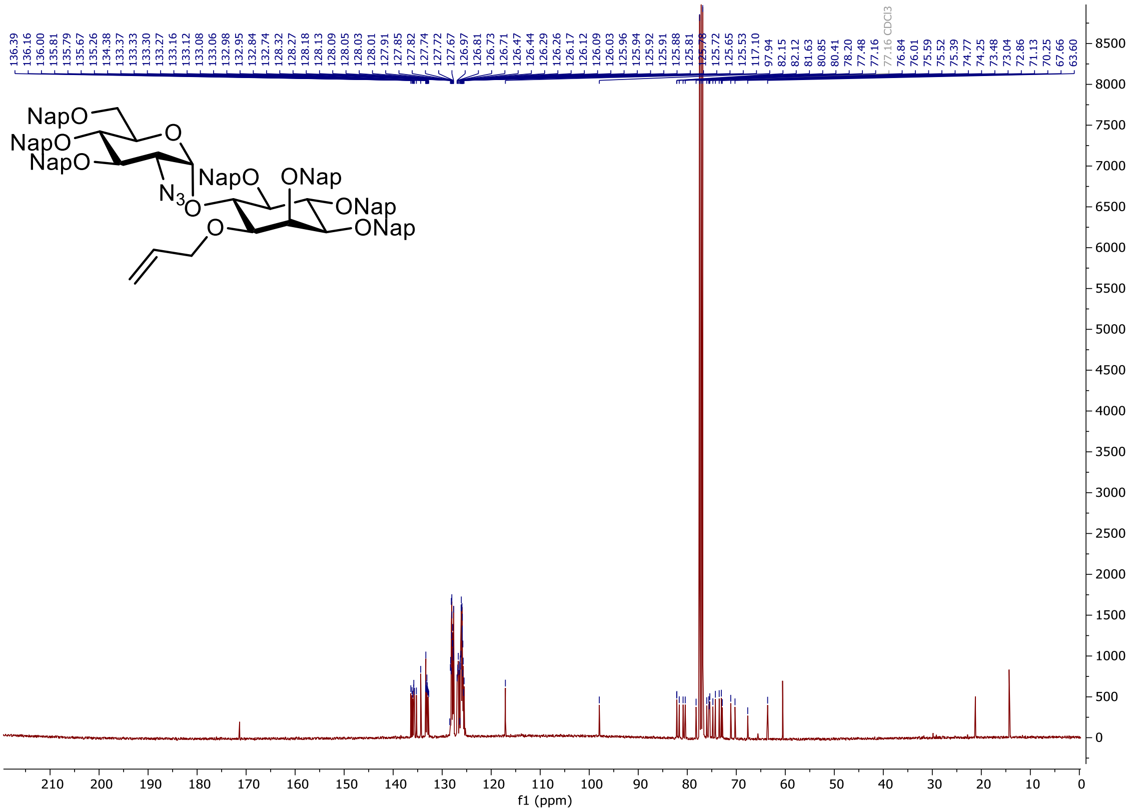

### $^{1}$H NMR spectrum of *pseudo*-disaccharide 11

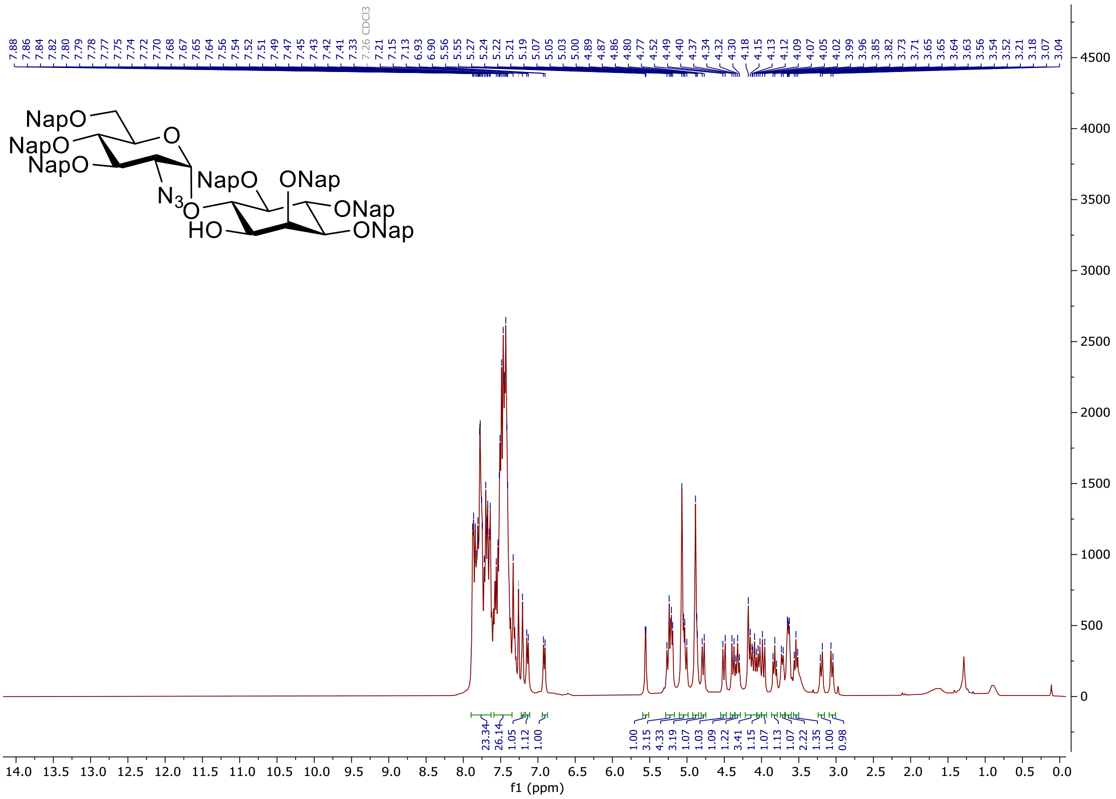

### $^{13}$C NMR spectrum of *pseudo*-disaccharide 11

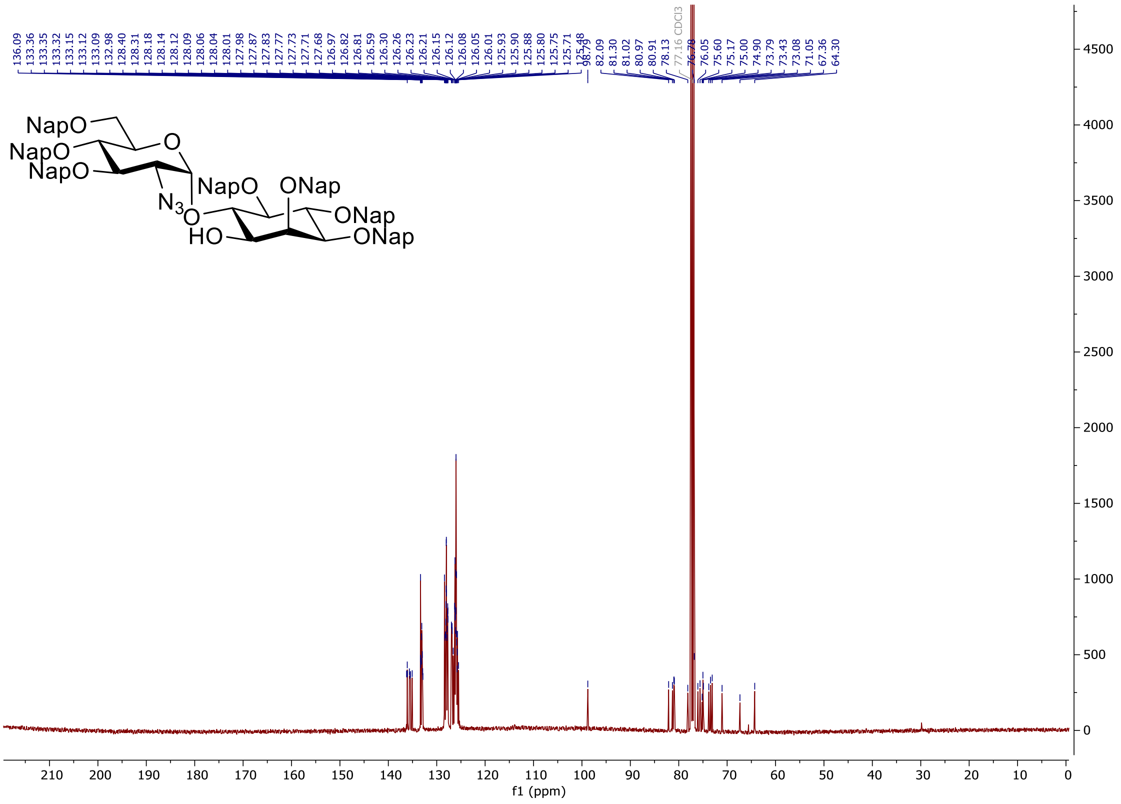

## $^{1}H$ NMR spectrum of protected glycolipid 12

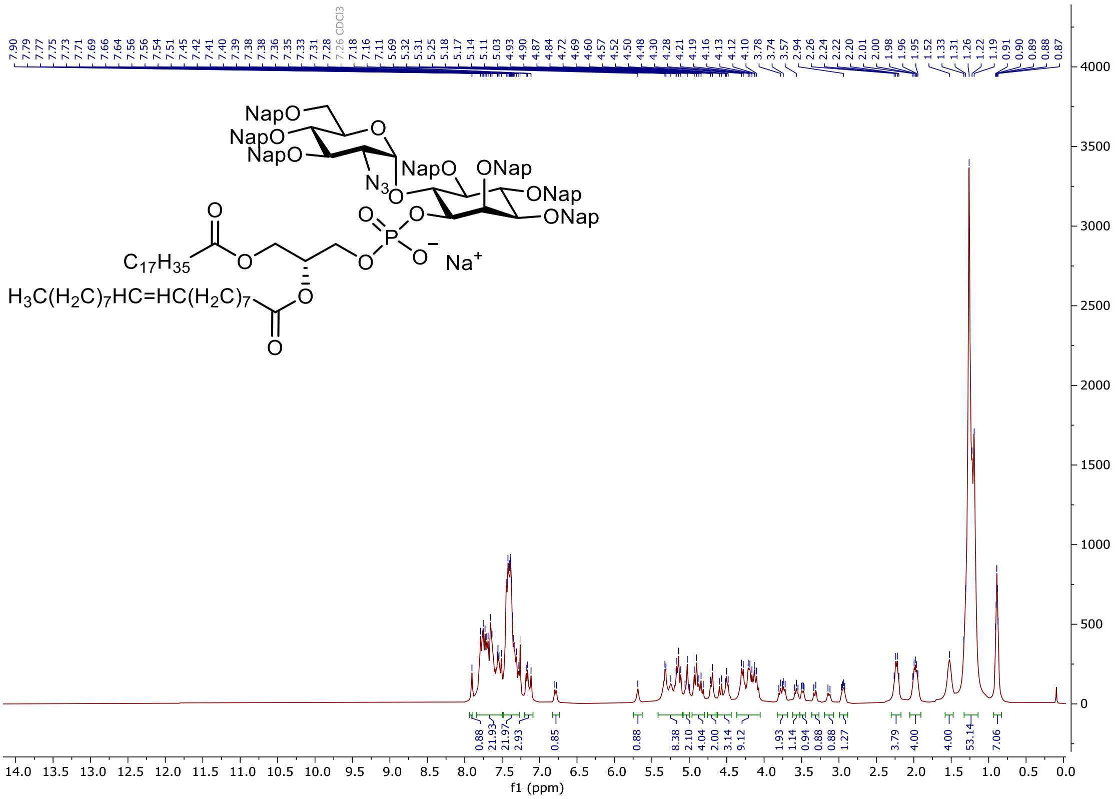


## $^{13}C$ NMR spectrum of protected glycolipid 12

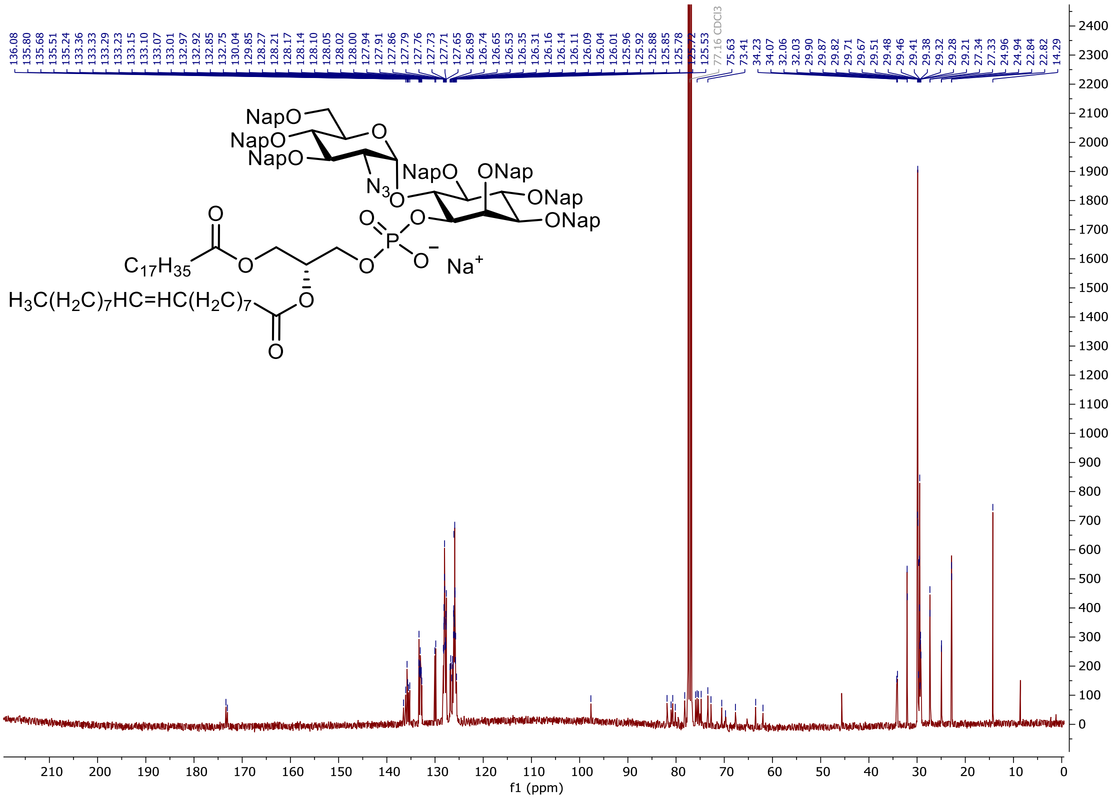

## COSY-NMR spectrum of protected glycolipid 12

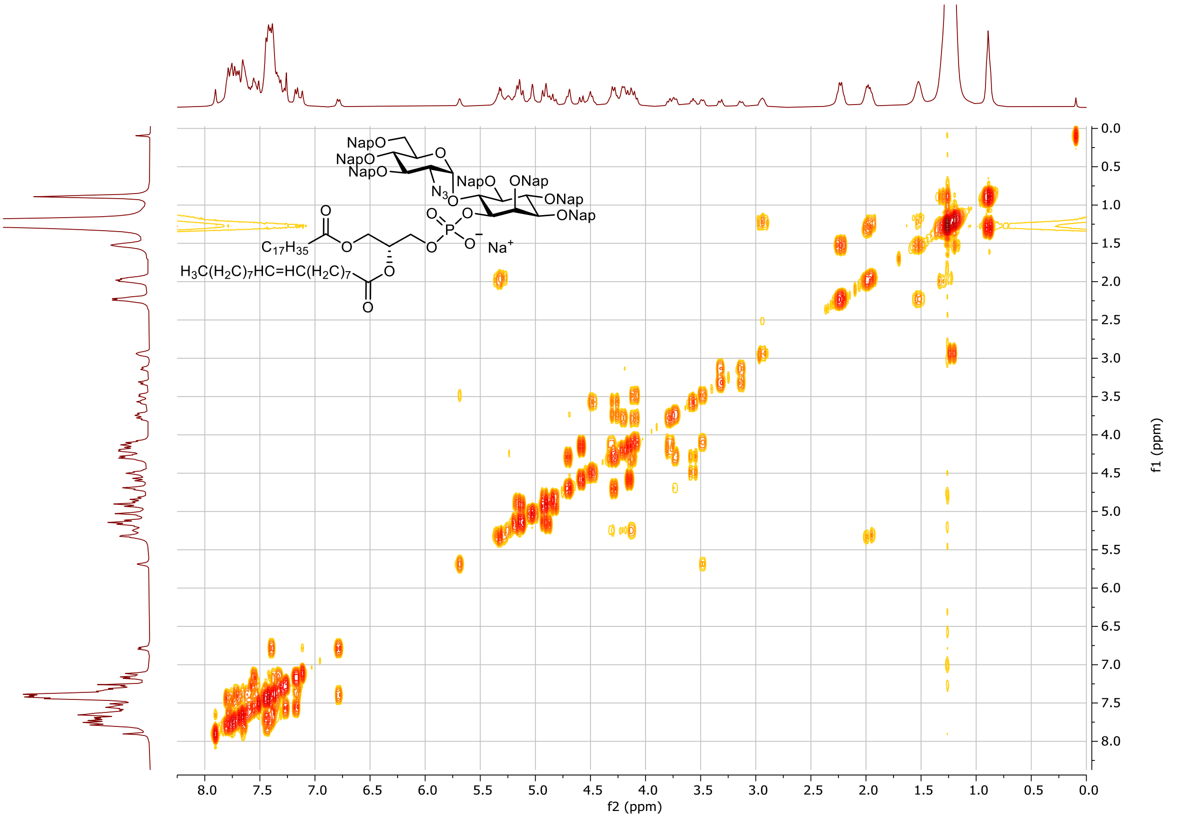


## HSQC-NMR spectrum of protected glycolipid 12

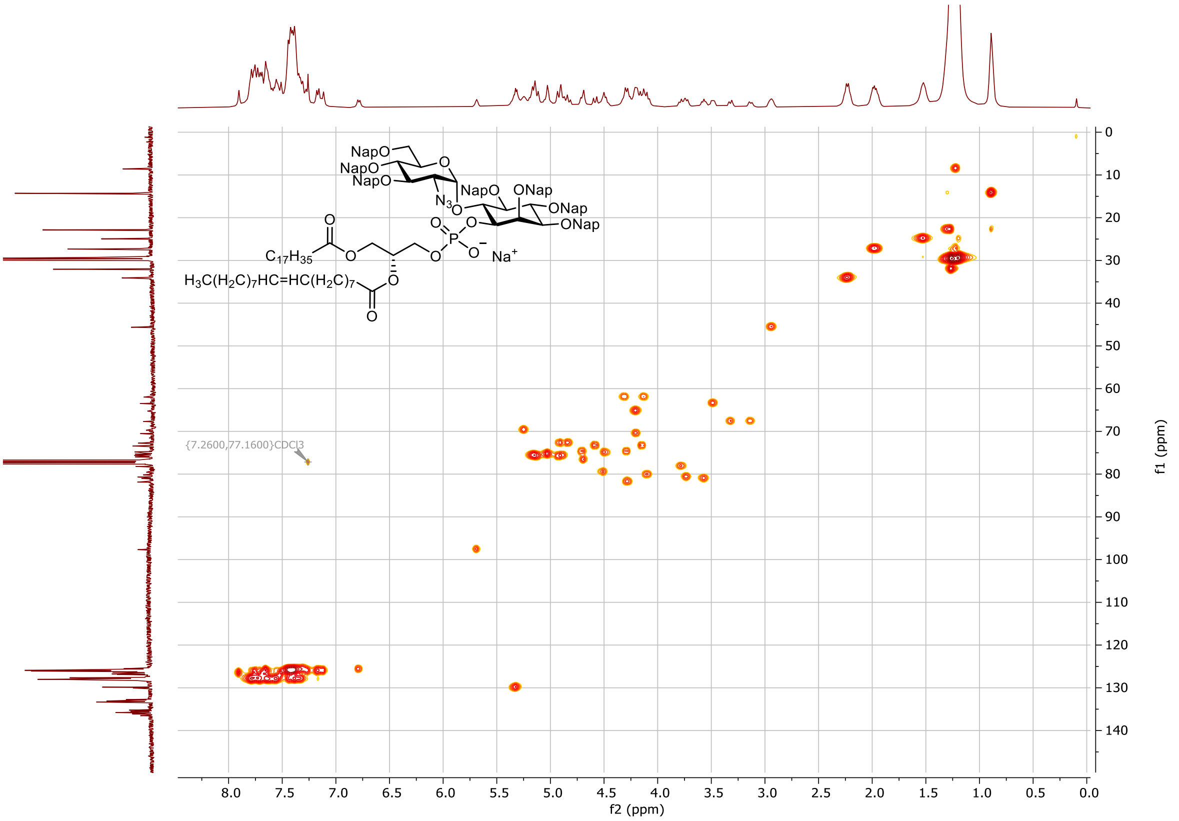

## $^{31}$P NMR spectrum of protected glycolipid 12

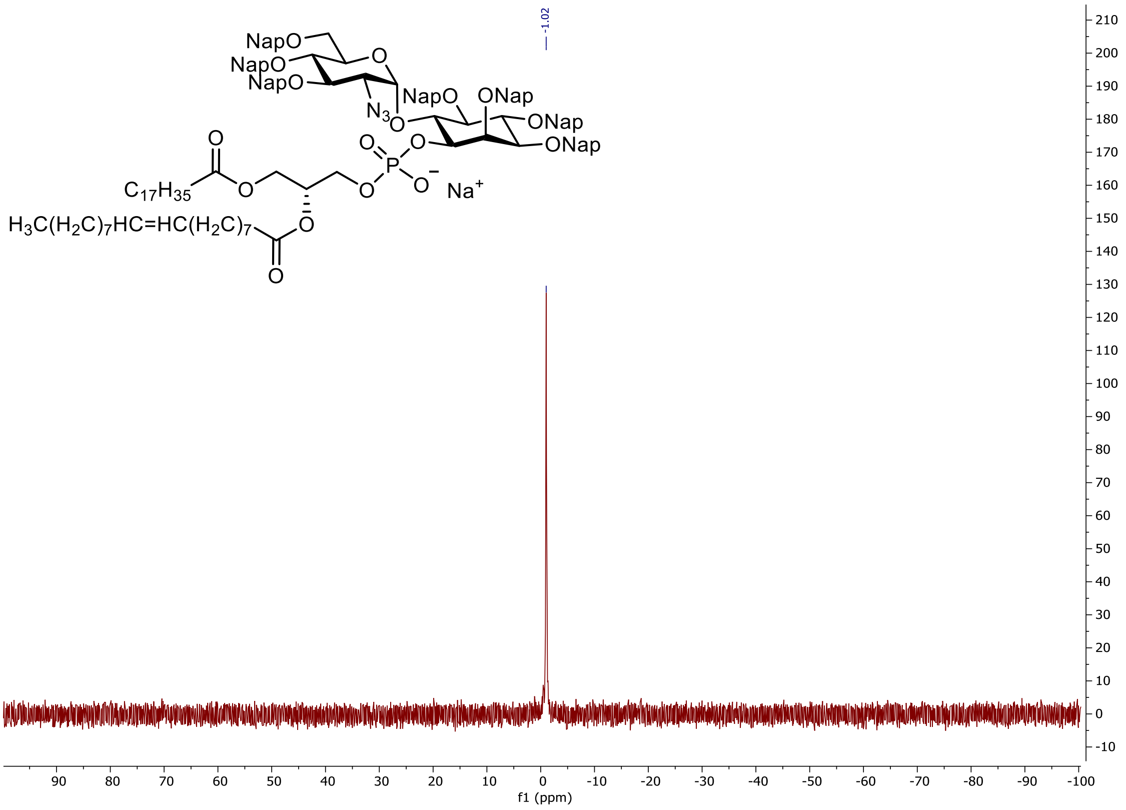


## $^{1}$H NMR spectrum of GPI-Fragment 1

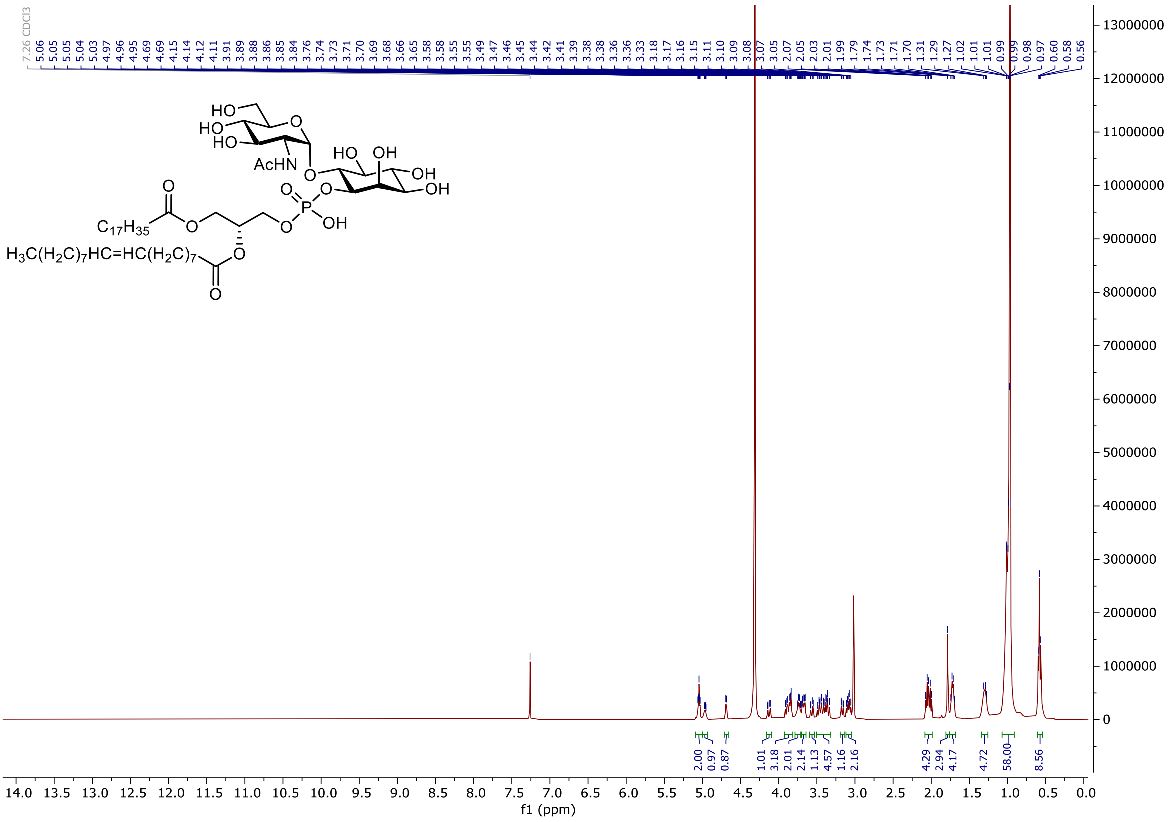

## $^{13}$C NMR spectrum of GPI-Fragment 1

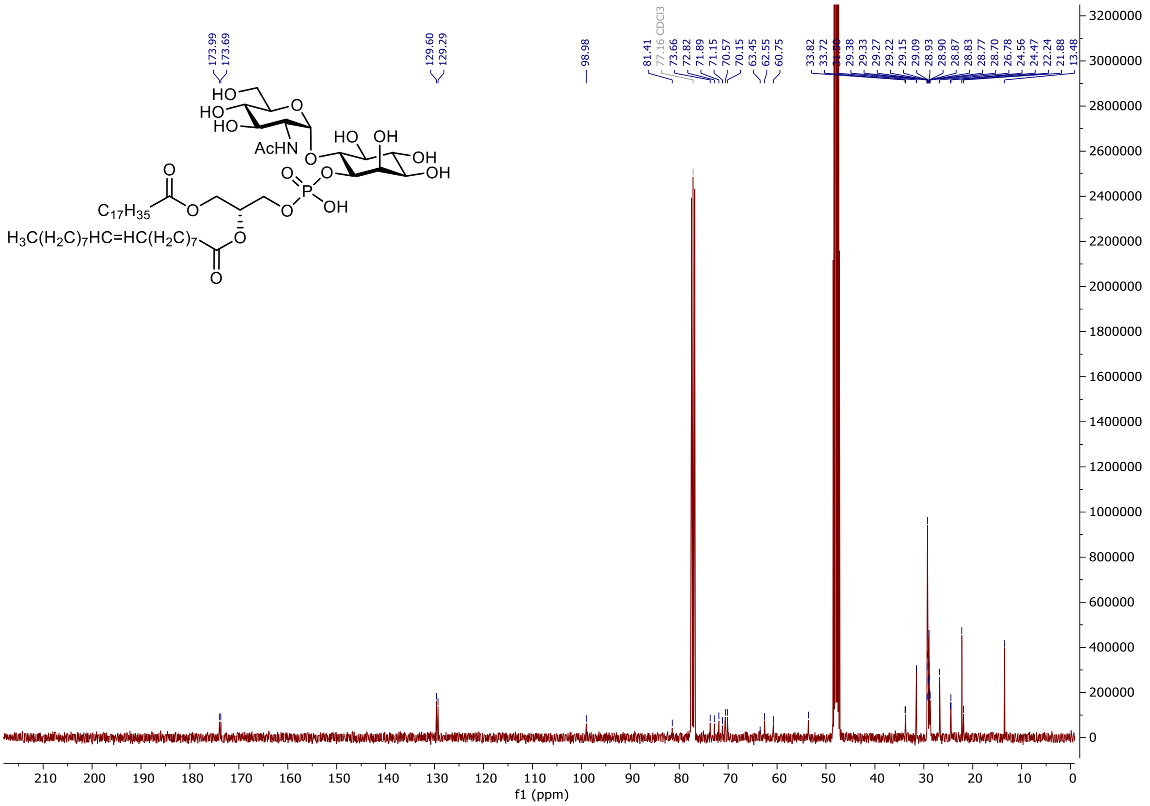

## COSY-NMR spectrum of GPI-Fragment 1

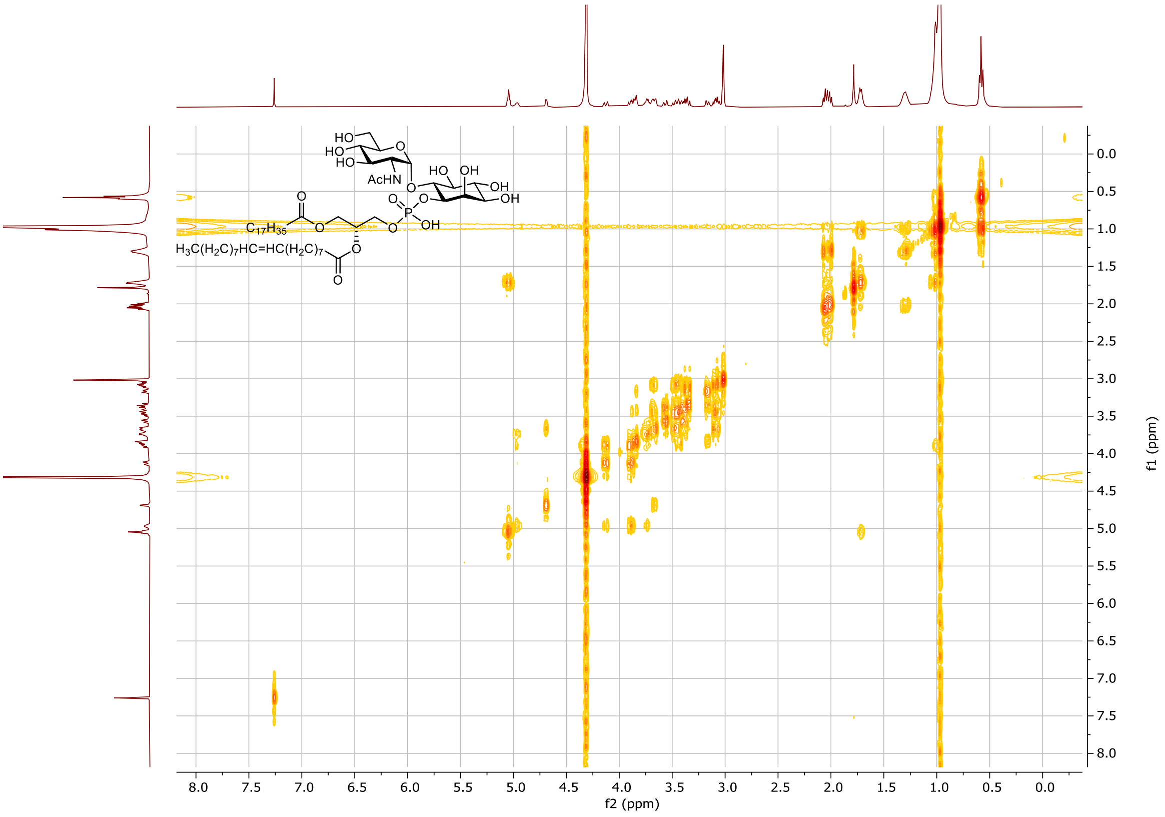

## HSQC-NMR spectrum of GPI-Fragment 1

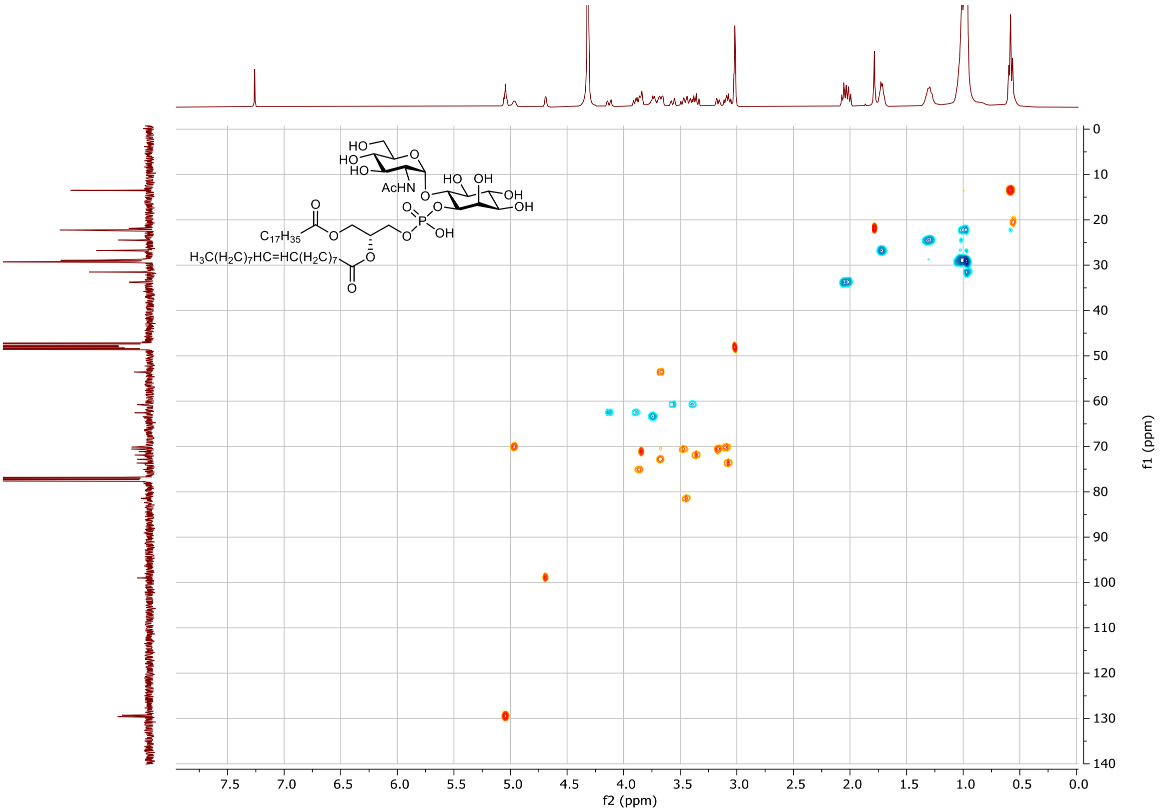


## $^{31}$P-NMR spectrum of GPI-Fragment 1

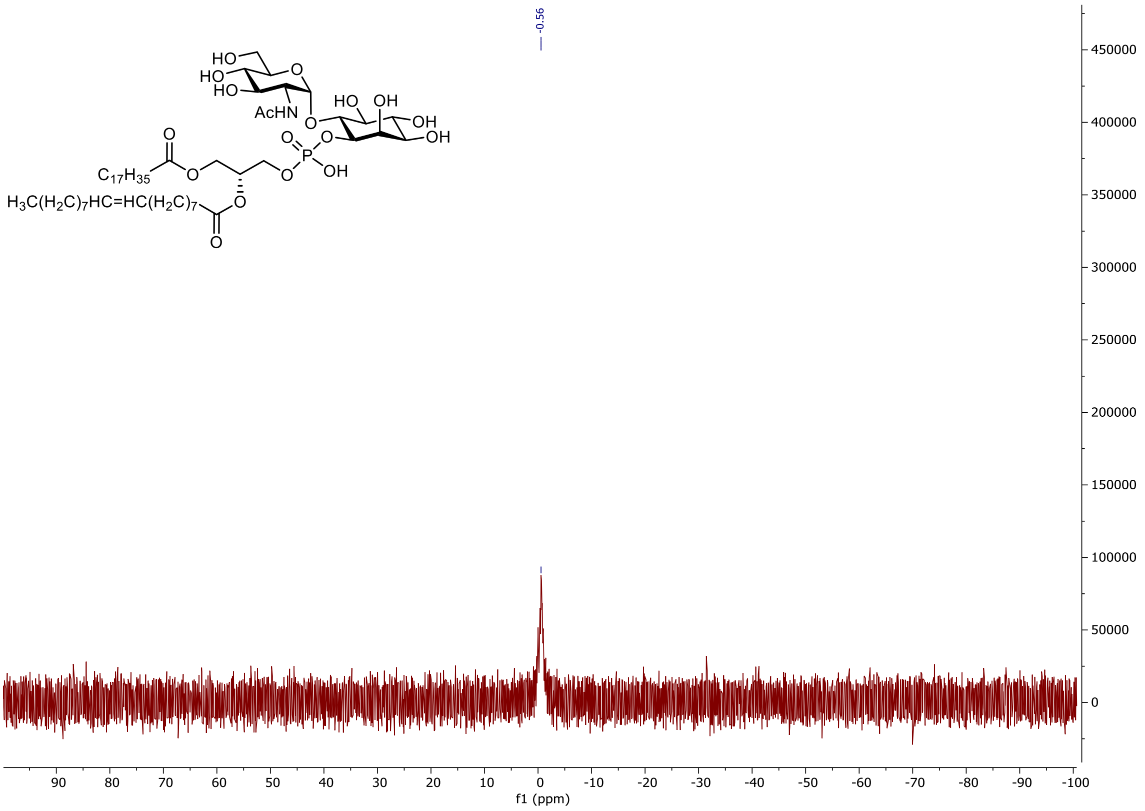

## $^{1}$H NMR spectrum of GPI-Fragment 3

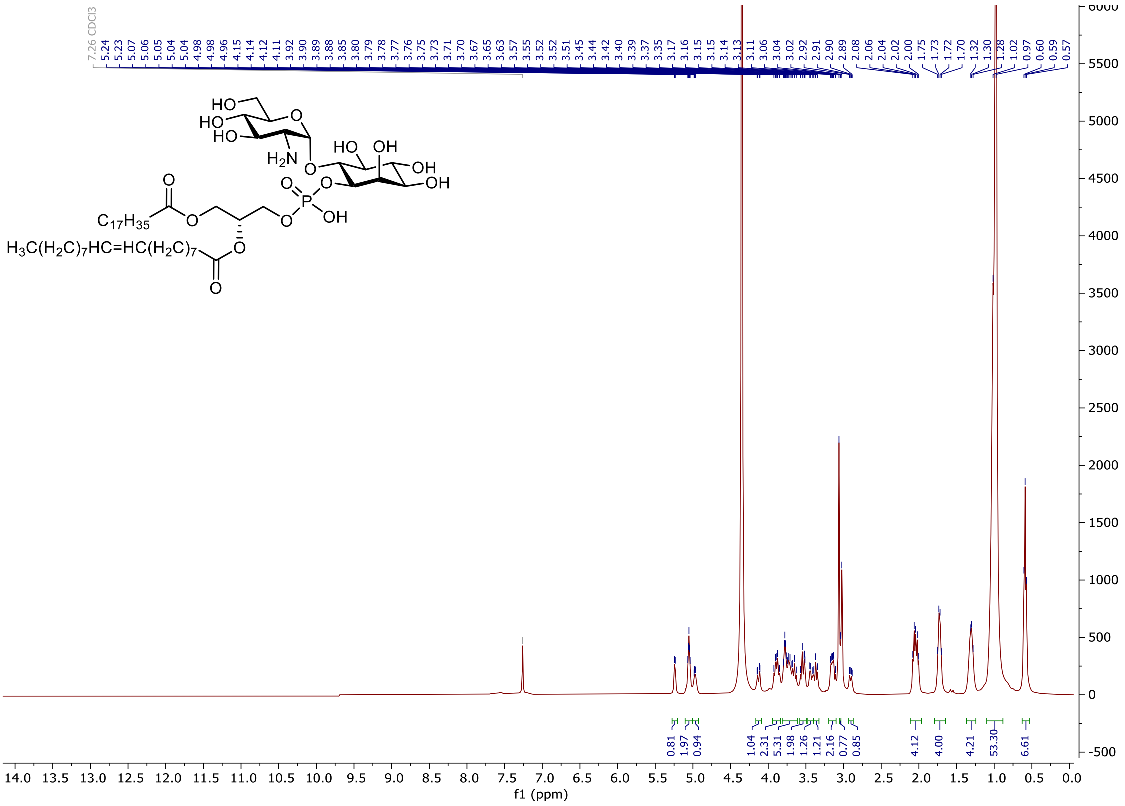

## $^{13}$C-NMR spectrum of GPI-Fragment 3

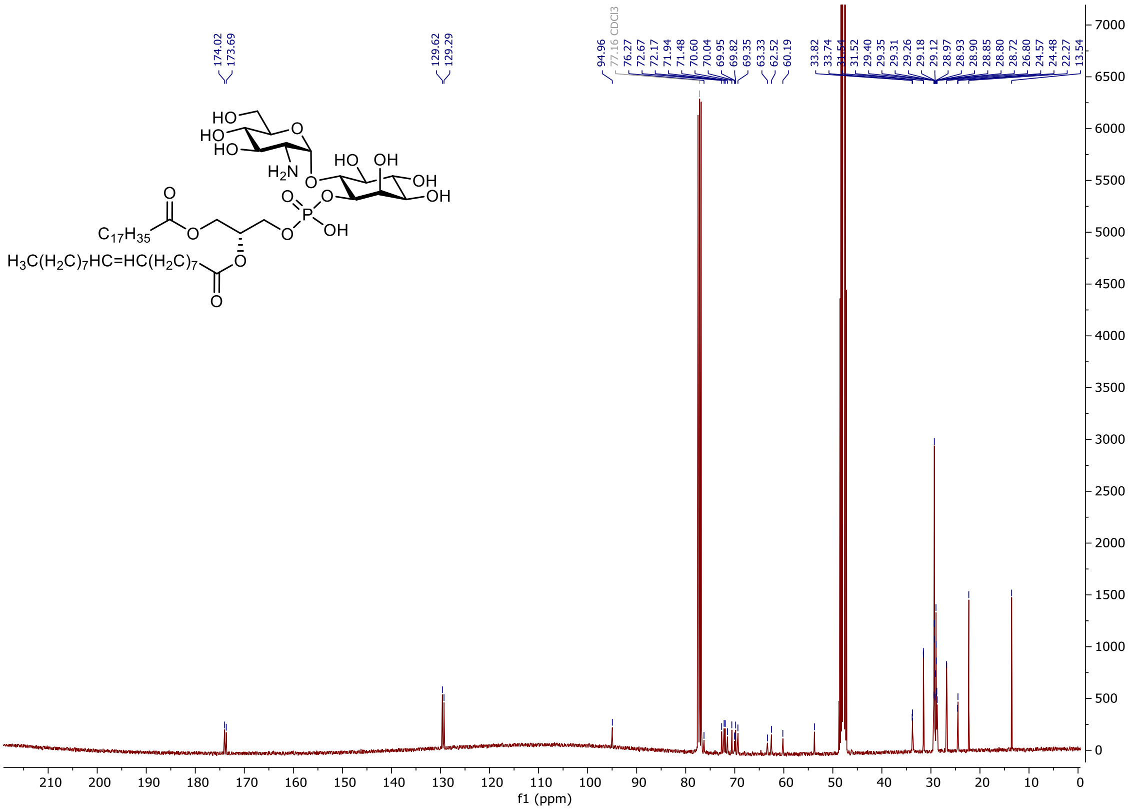

## COSY-NMR spectrum of GPI-Fragment 3

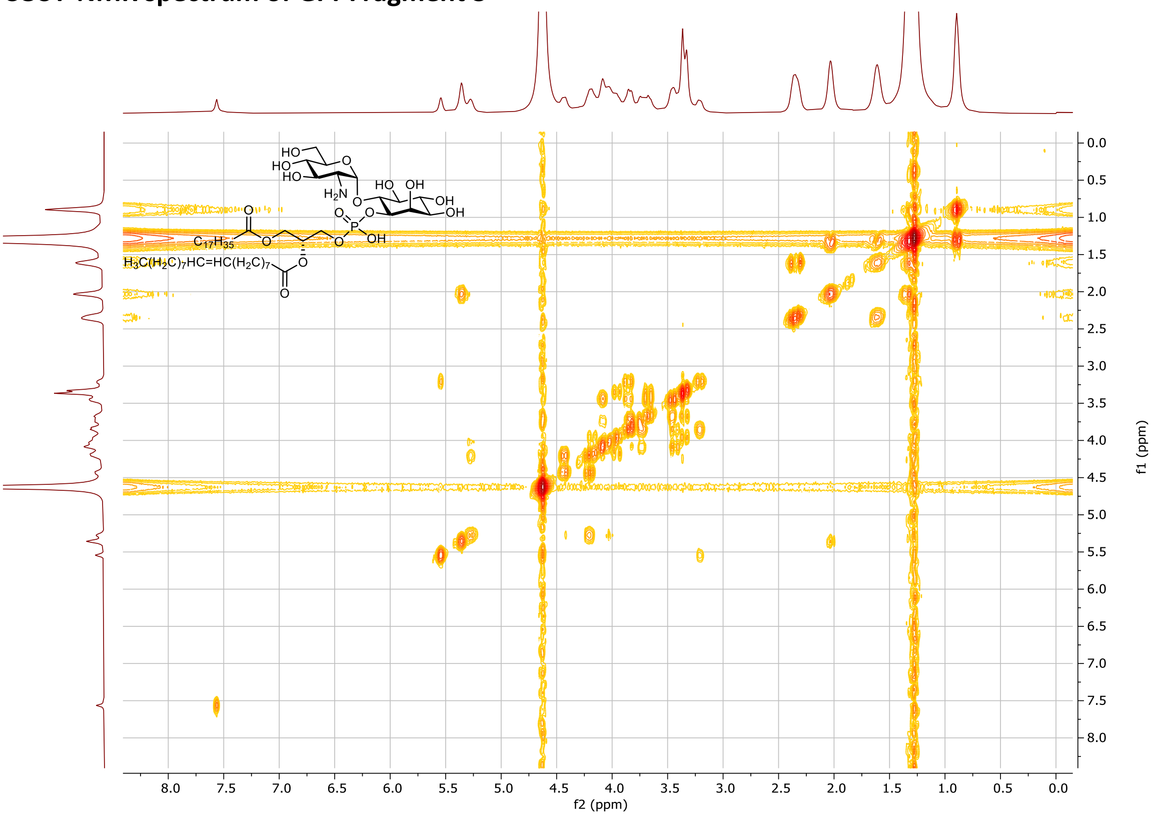

## HSQC-NMR spectrum of GPI-Fragment 3

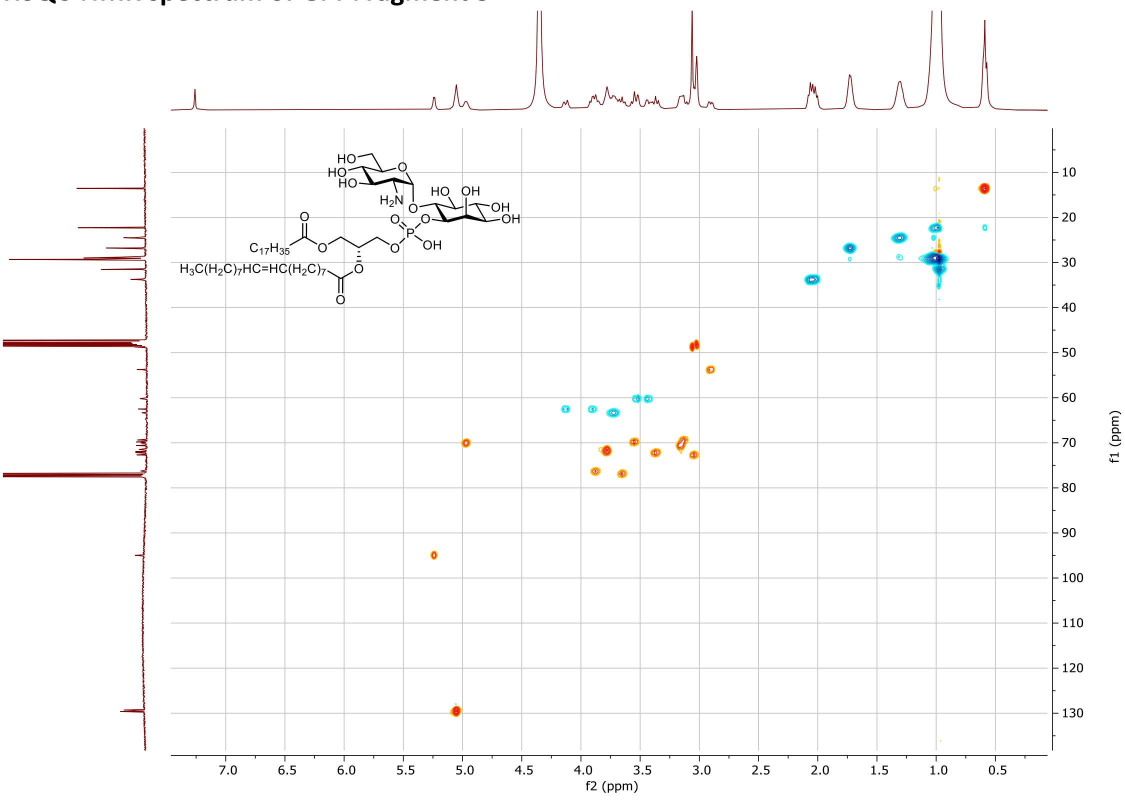

**$^{31}$P-NMR spectrum of GPI-Fragment 3**

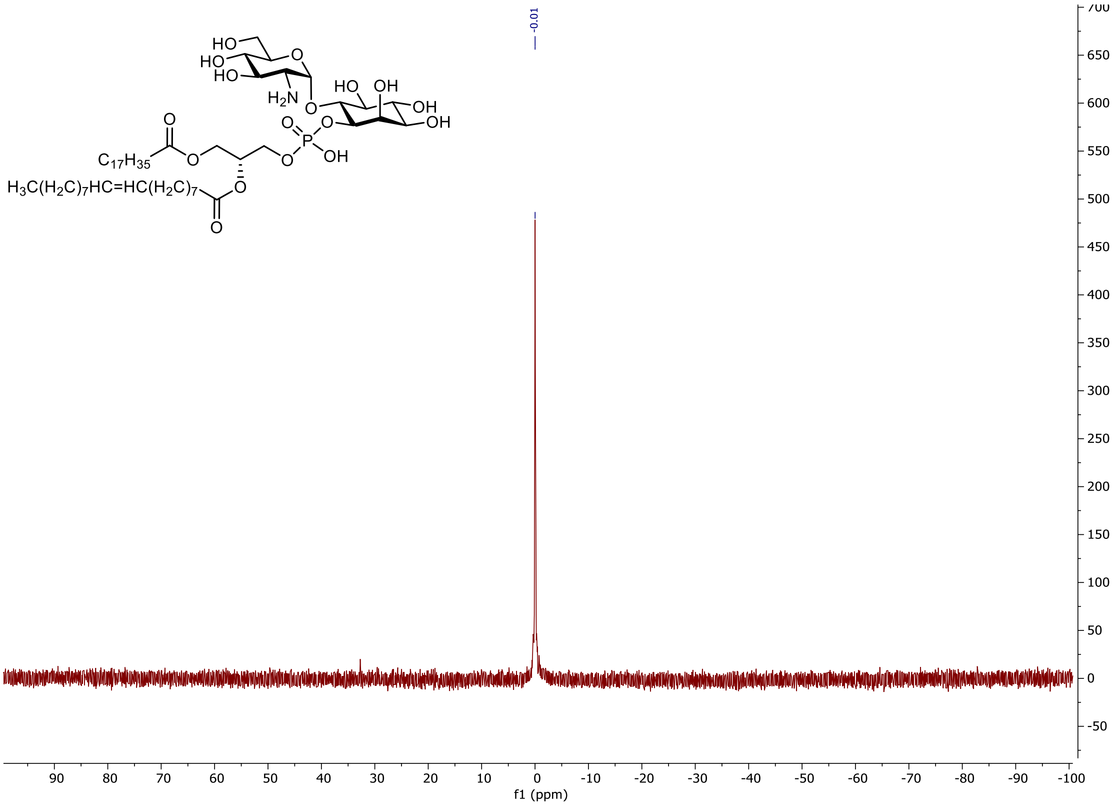

**$^{1}$H NMR spectrum of *pseudo*-disaccharide 16**

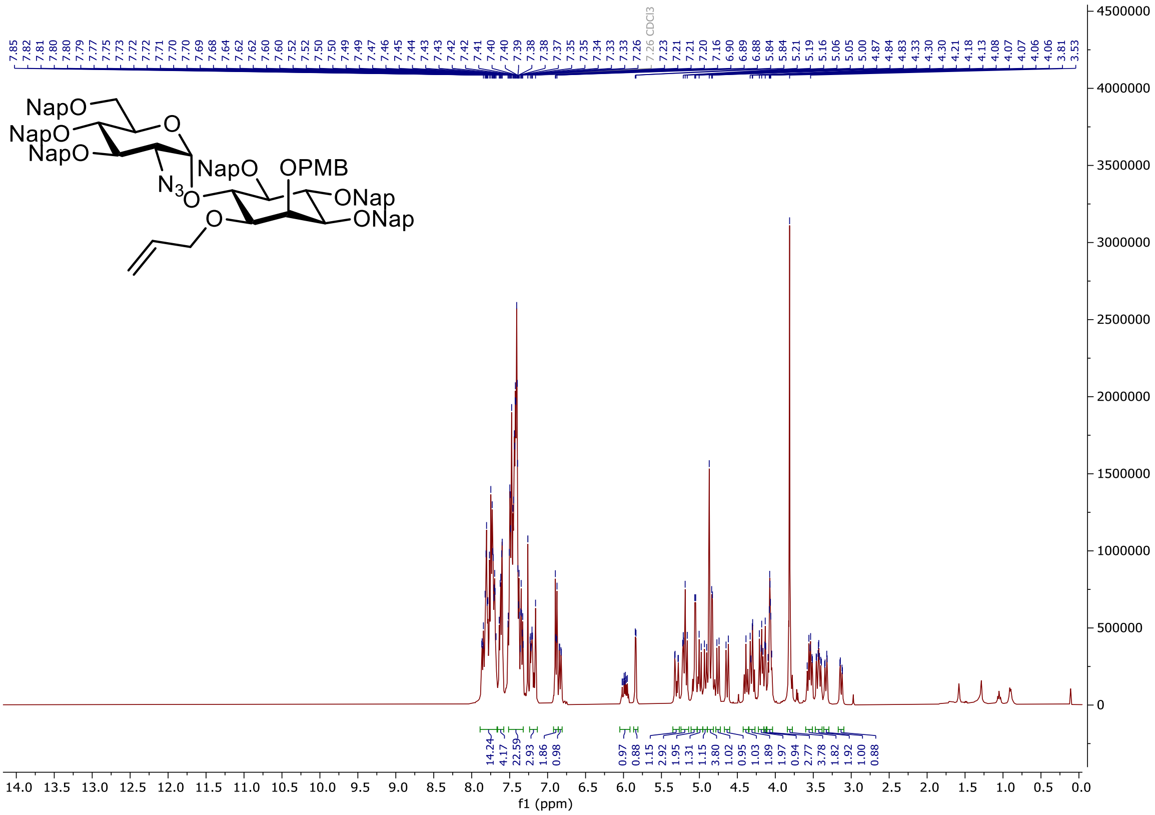

## $^{13}$C NMR spectrum of *pseudo*-disaccharide 16

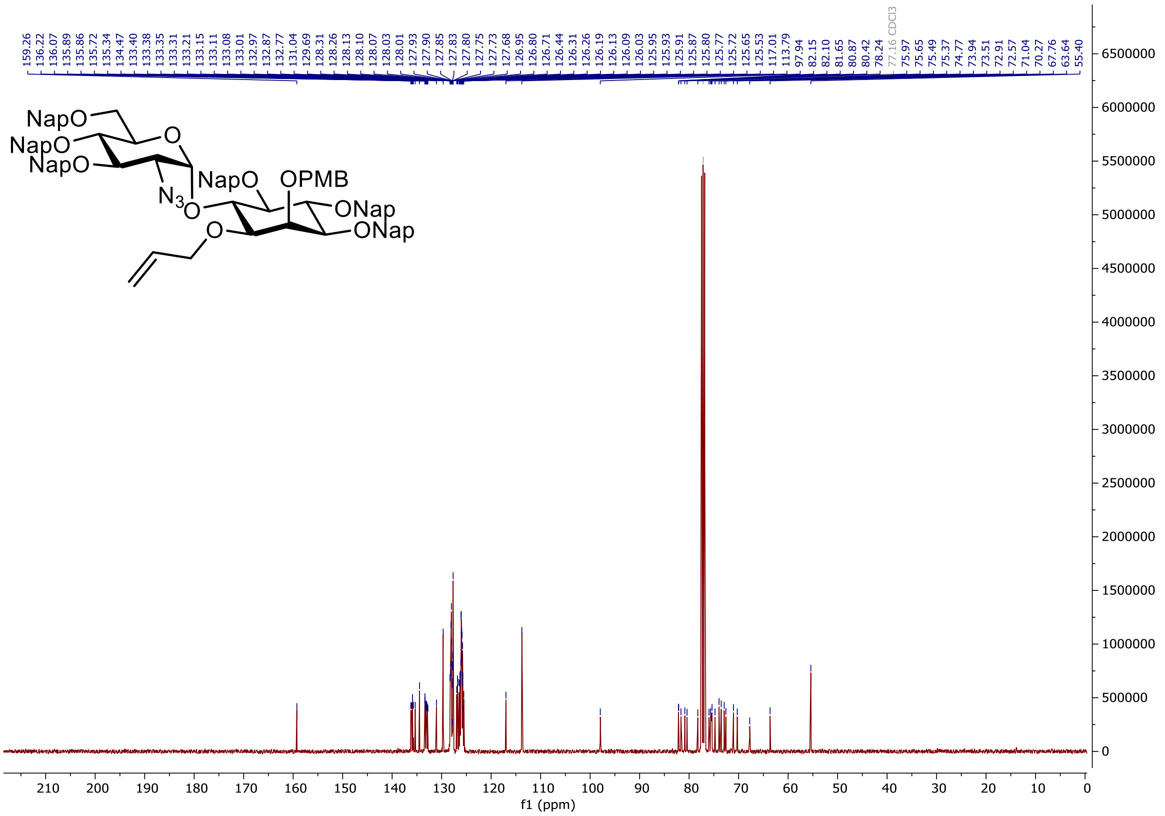

## COSY-NMR spectrum of *pseudo*-disaccharide 16

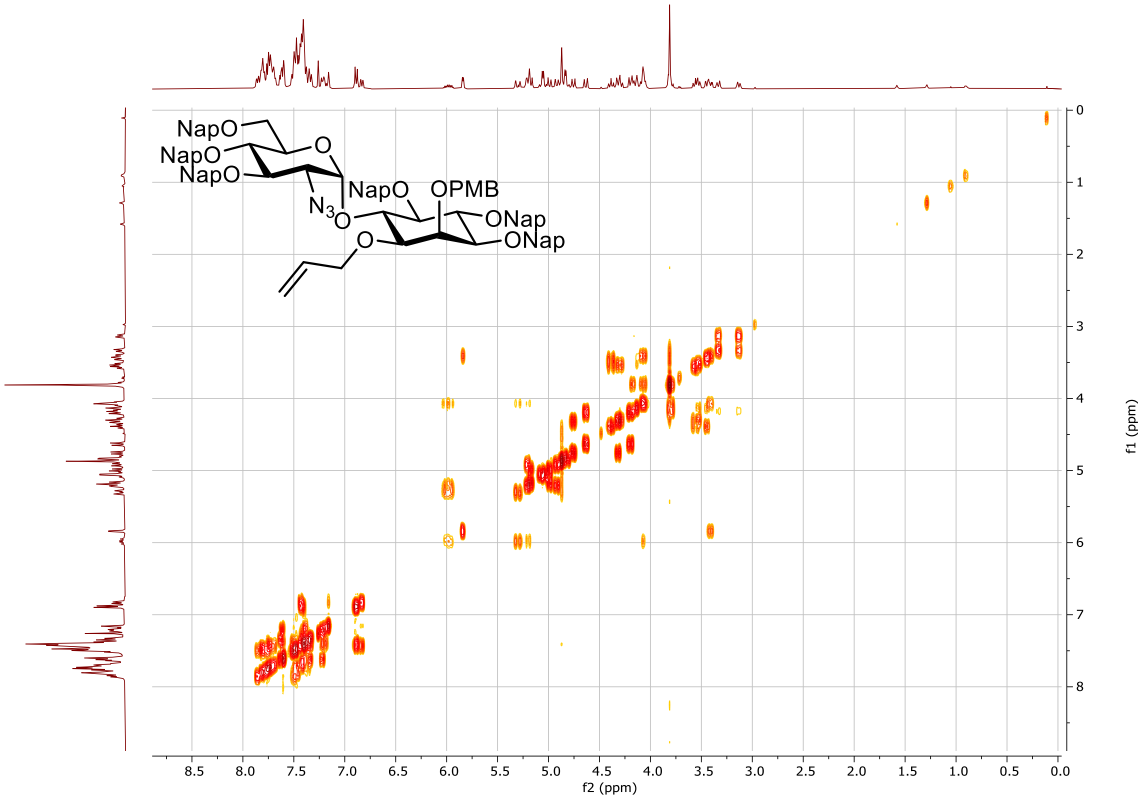

## HSQC-NMR spectrum of *pseudo*-disaccharide 16

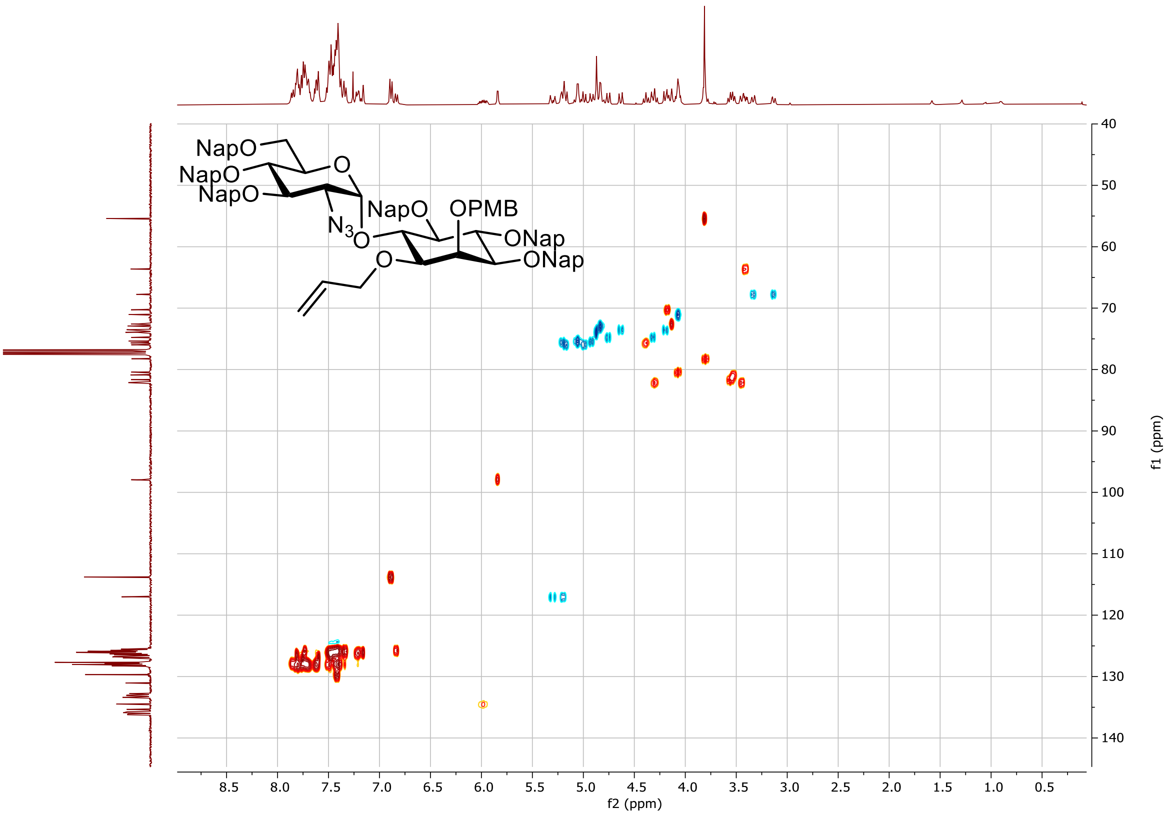


## $^{1}$H NMR spectrum of *pseudo*-disaccharide 17

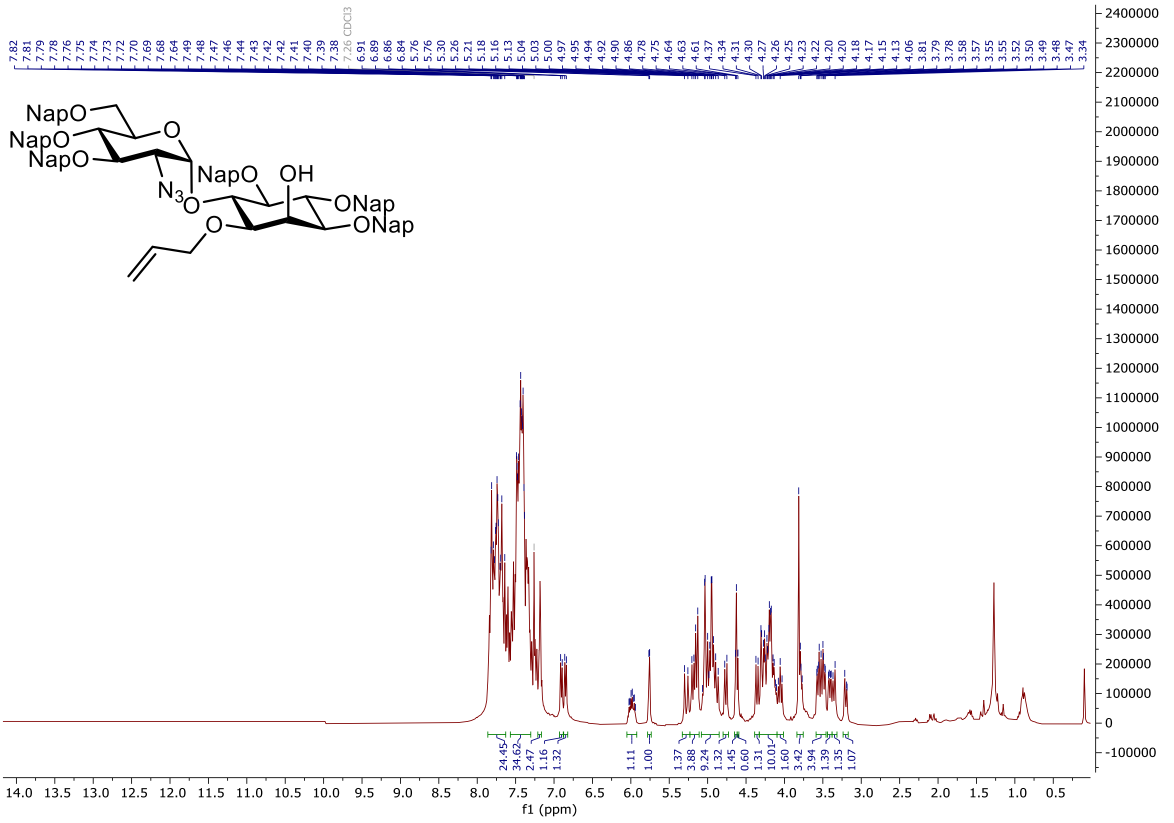

## $^{13}$C NMR spectrum of *pseudo*-disaccharide 17

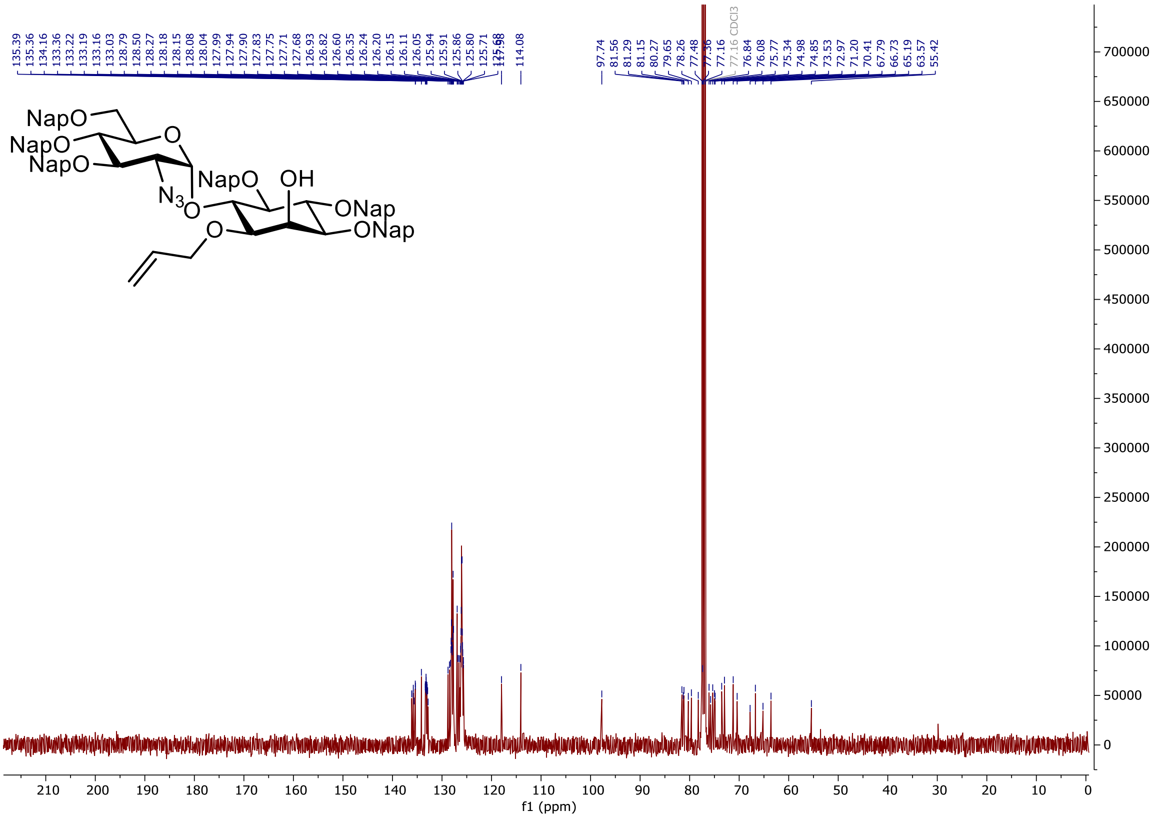

## $^{1}$H NMR spectrum of *pseudo*-disaccharide 7

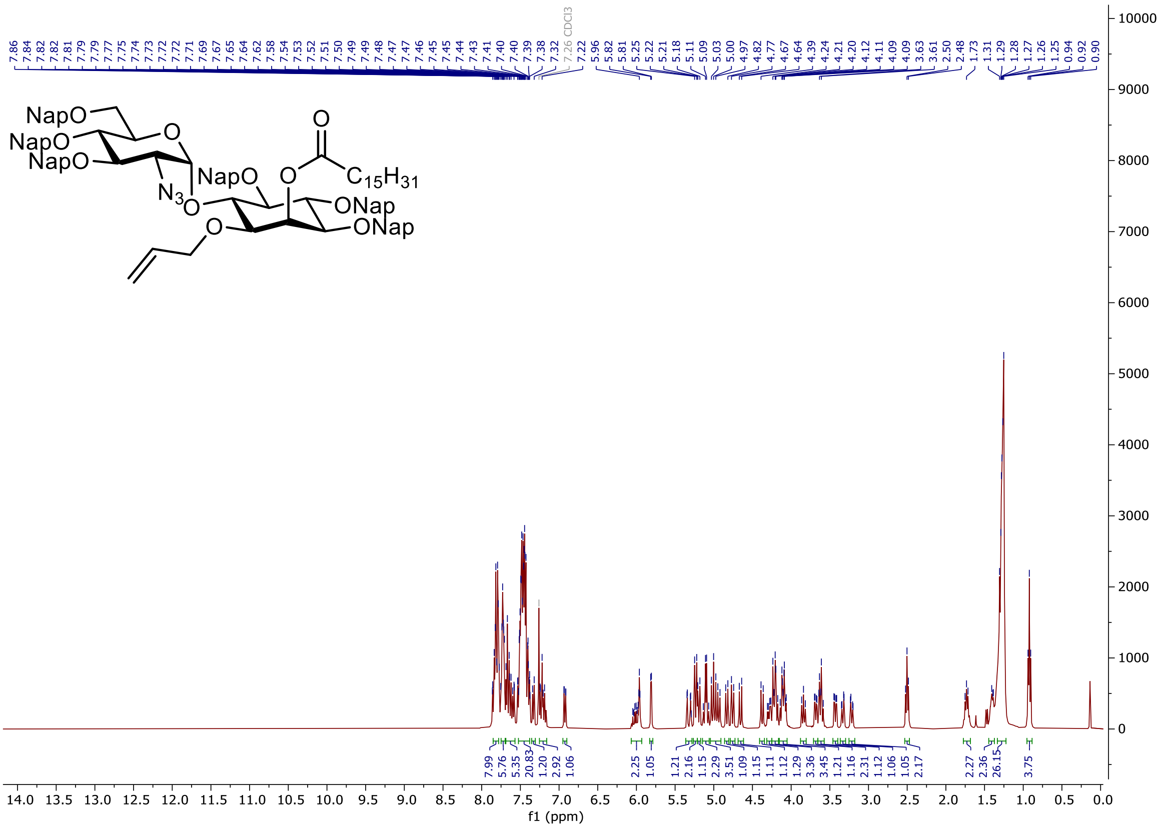

## $^{13}$C NMR spectrum of *pseudo*-disaccharide 7

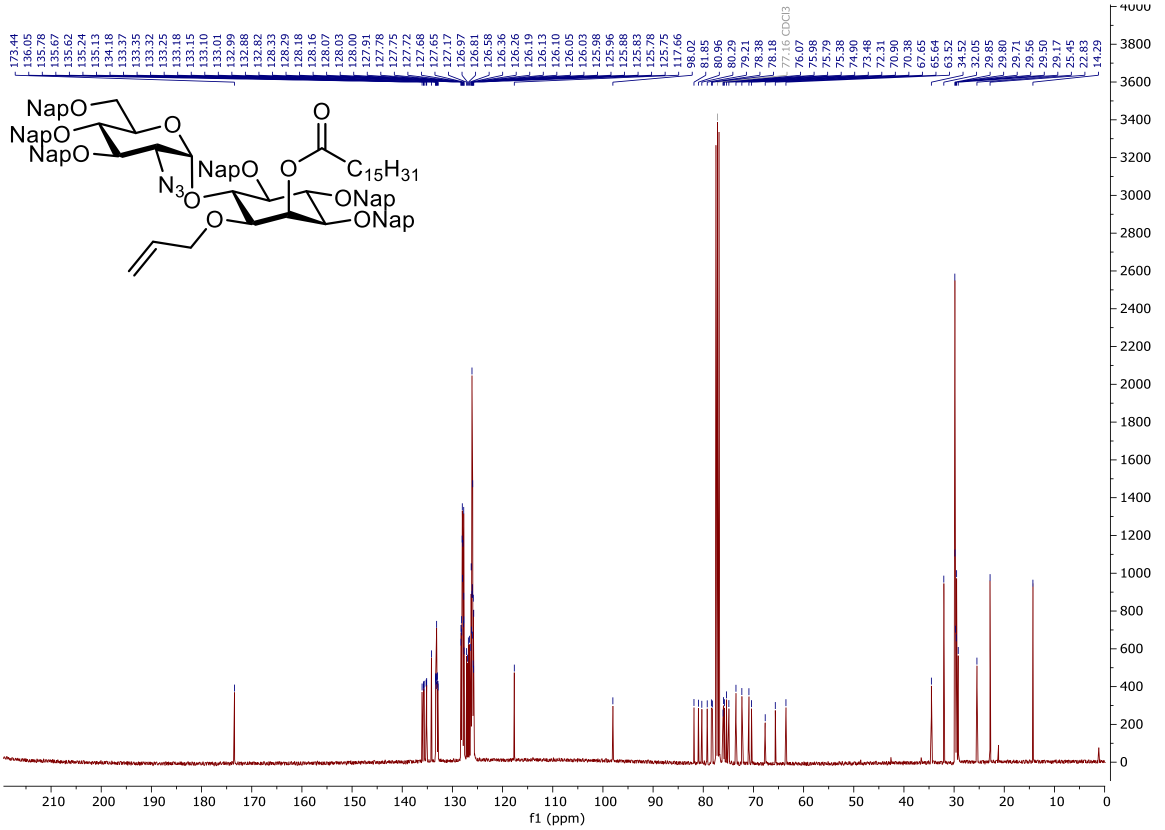

## COSY-NMR spectrum of *pseudo*-disaccharide 7

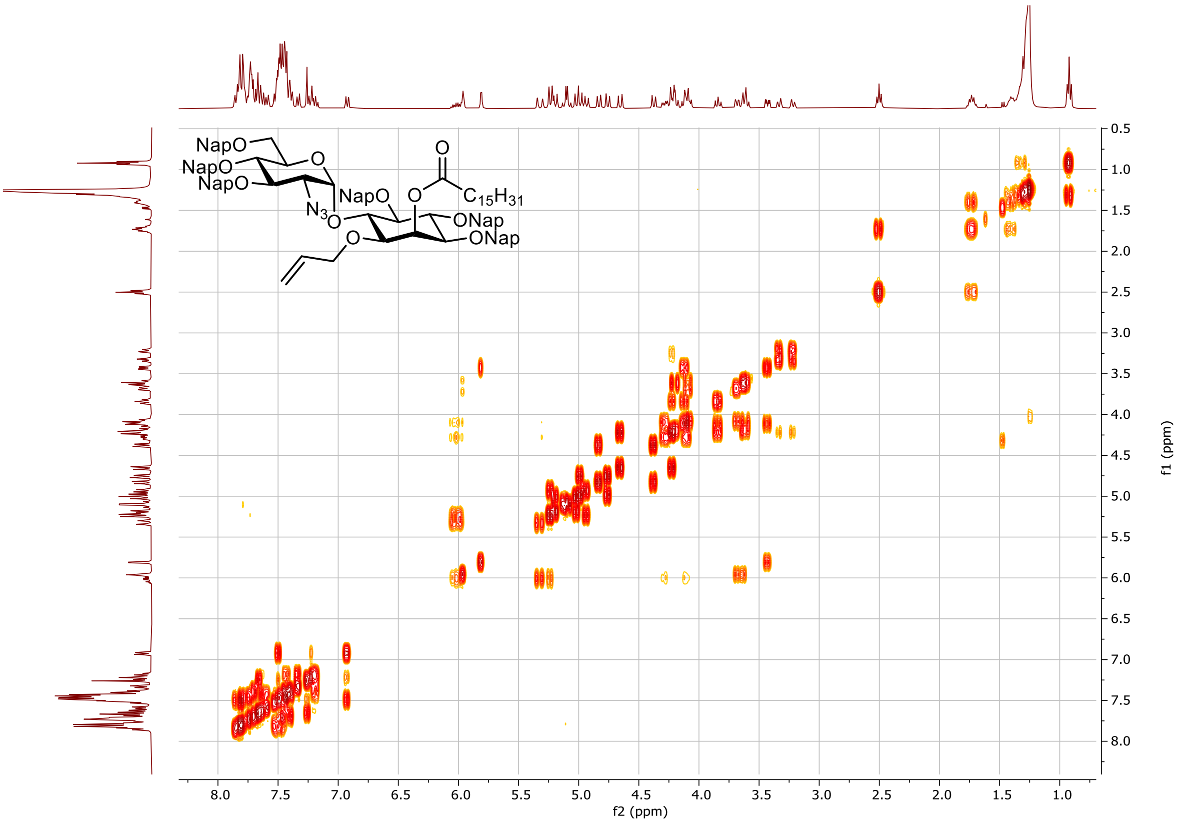

## HSQC-NMR spectrum of *pseudo*-disaccharide 7

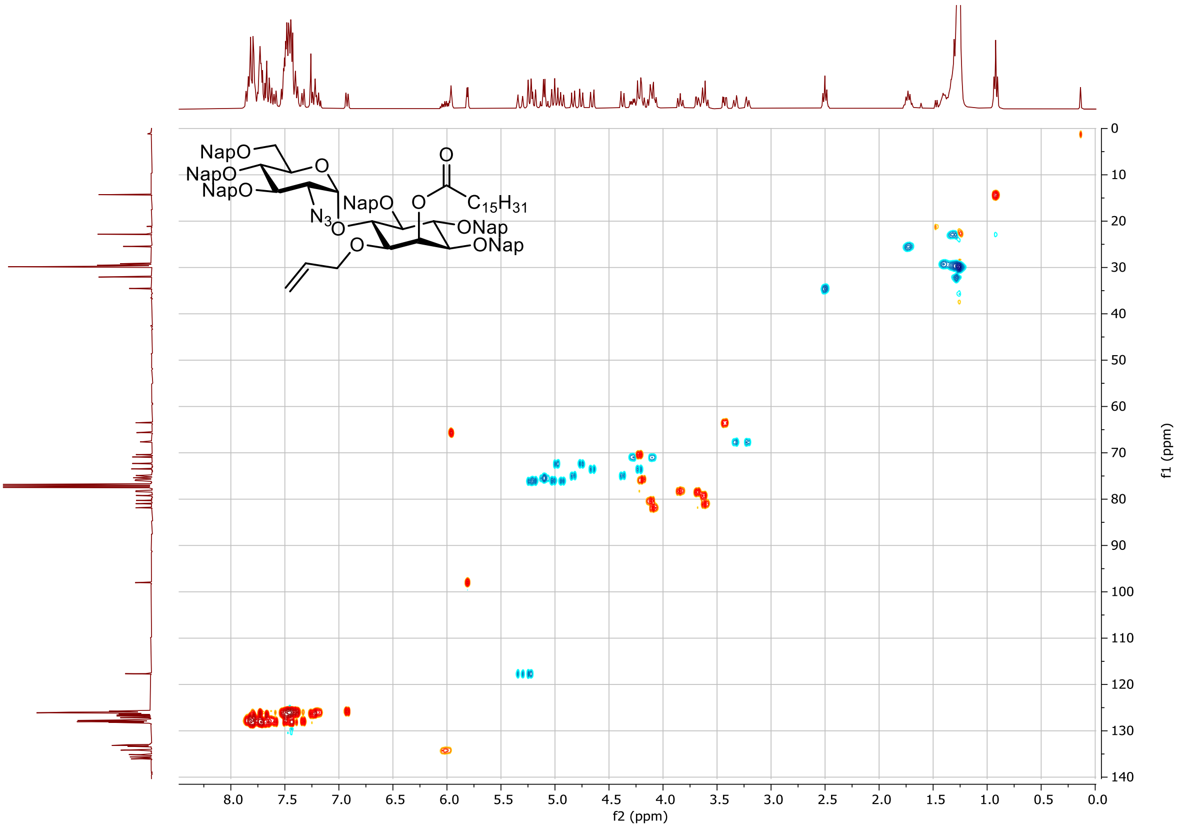

## $^{1}$H NMR spectrum of *pseudo*-disaccharide 18

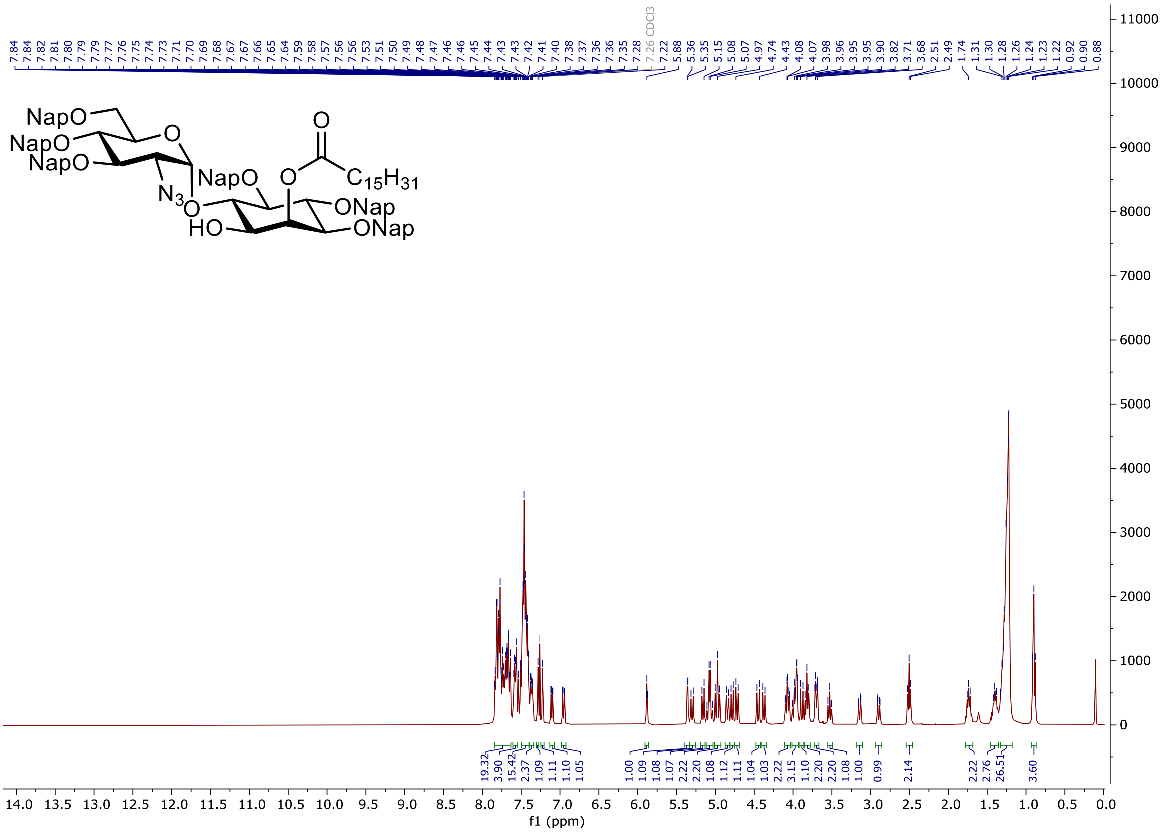

## $^{13}$C NMR spectrum of *pseudo*-disaccharide18

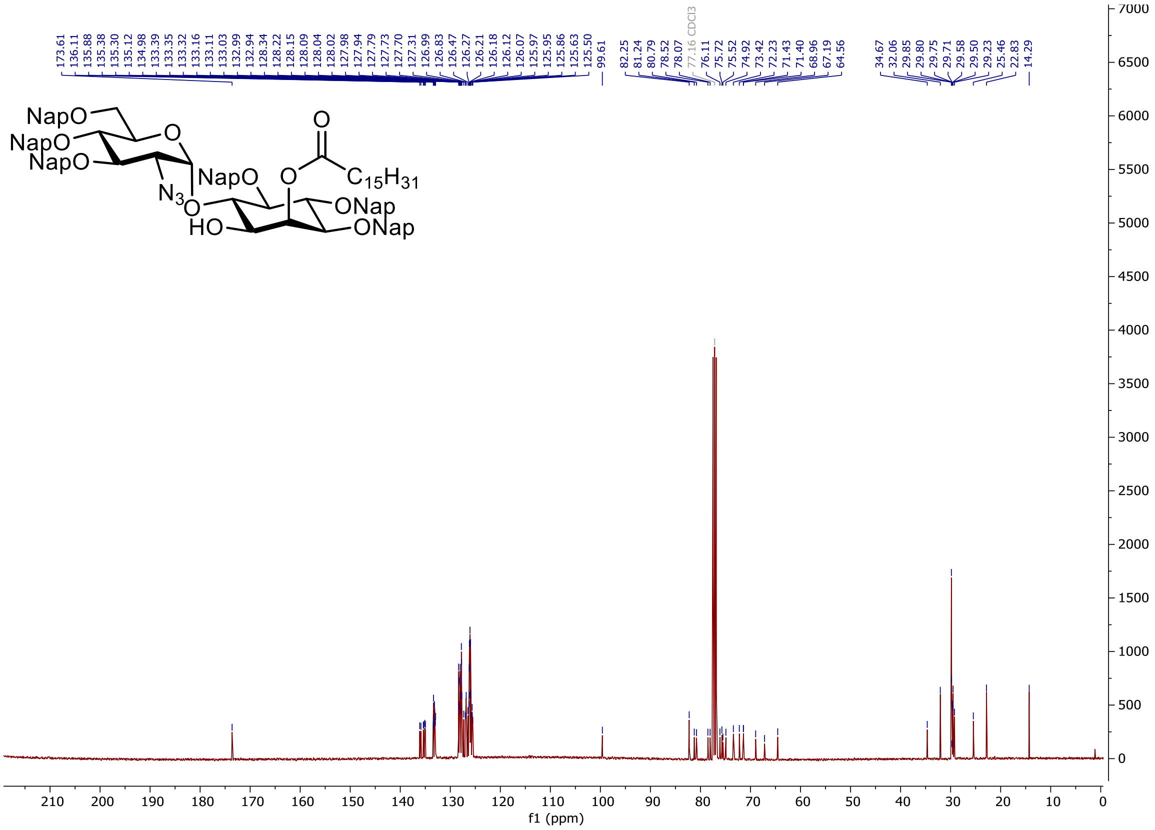

## COSY-NMR spectrum of *pseudo*-disaccharide 18

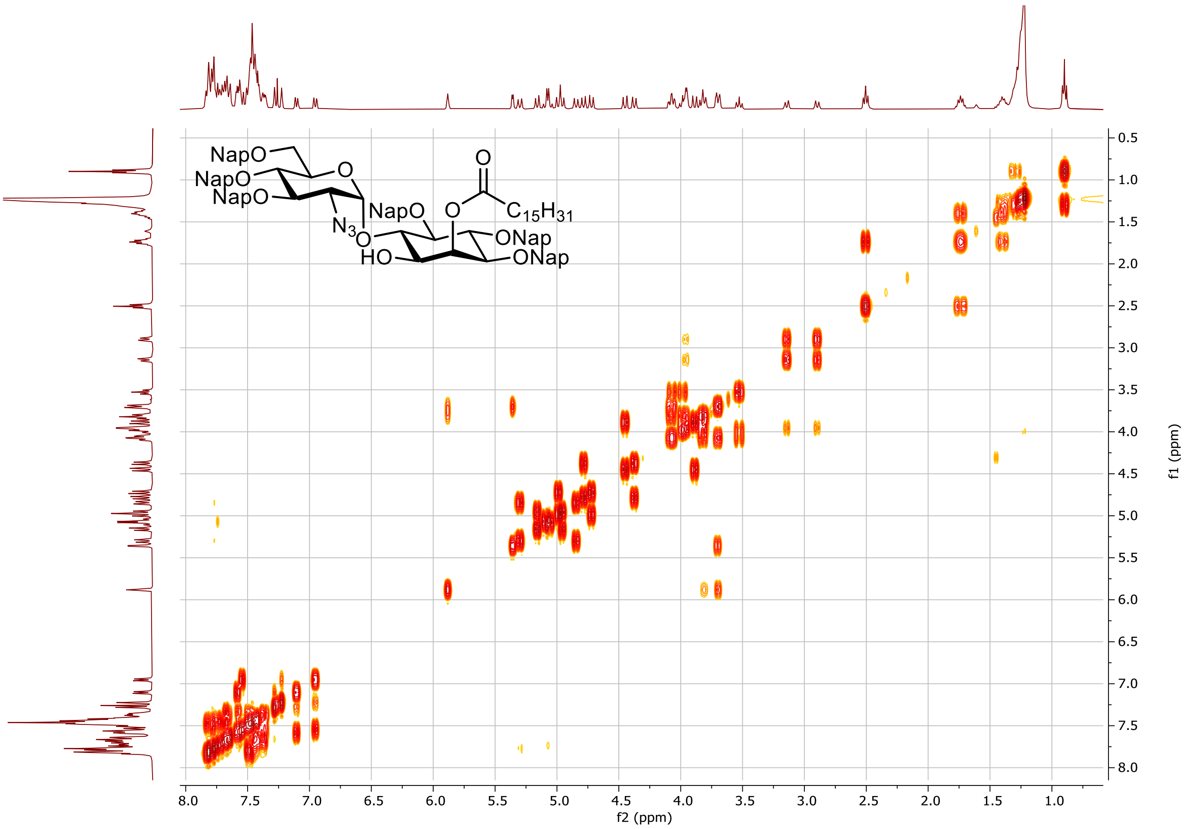

## HSQC-NMR spectrum of *pseudo*-disaccharide 18

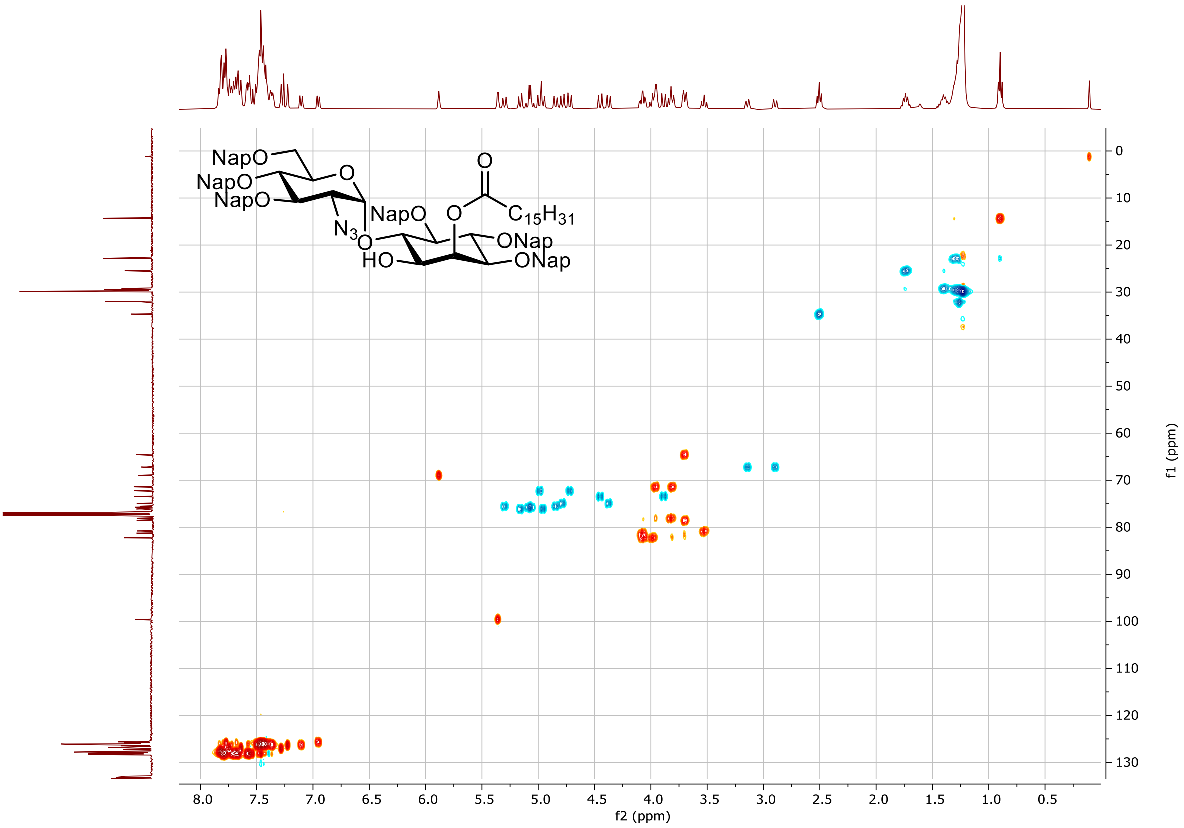

## $^{1}$H NMR spectrum of protected glycolipid 19

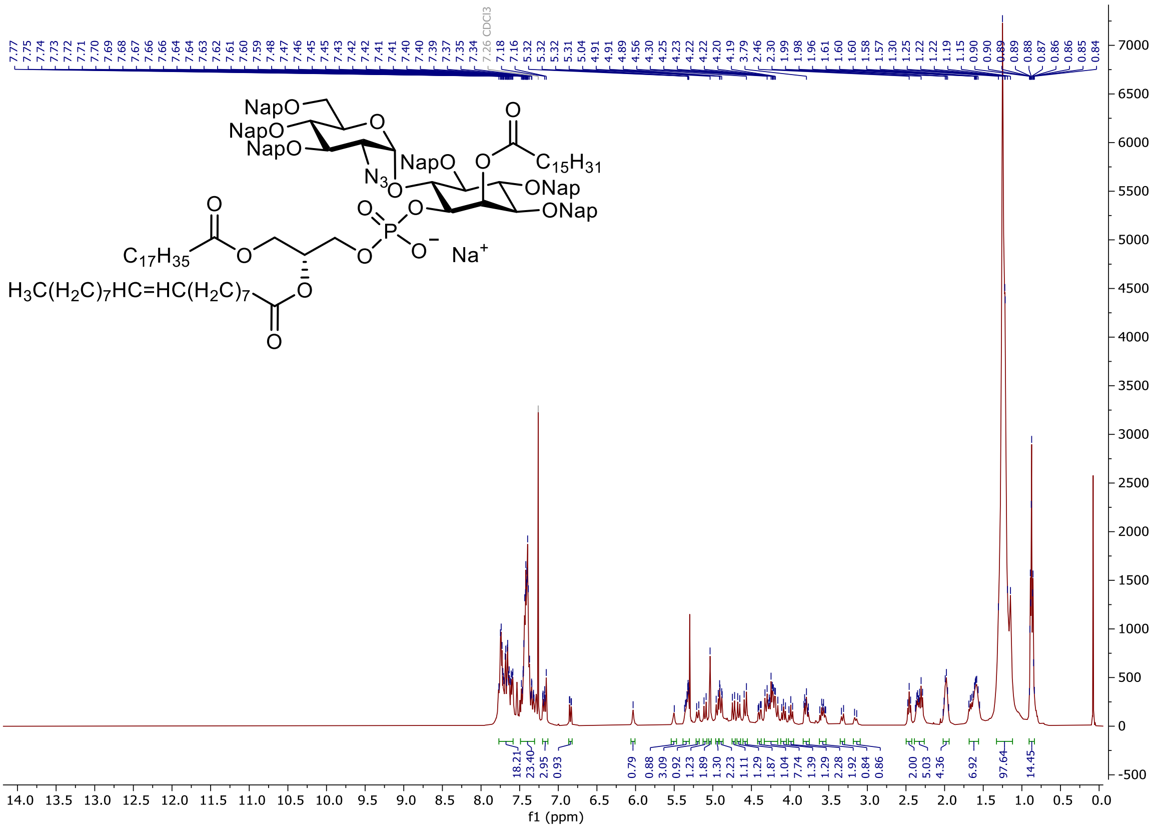

## $^{13}C$ NMR spectrum of protected glycolipid 19

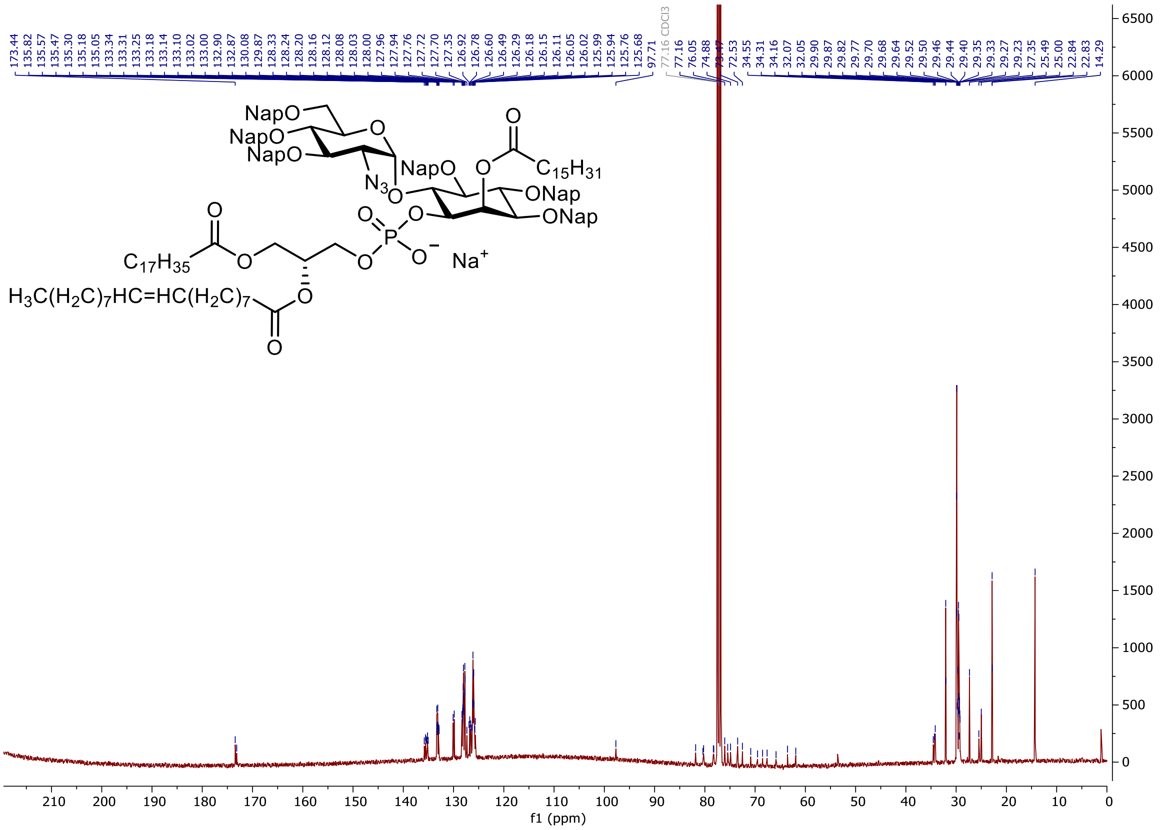

## COSY-NMR spectrum of protected glycolipid 19

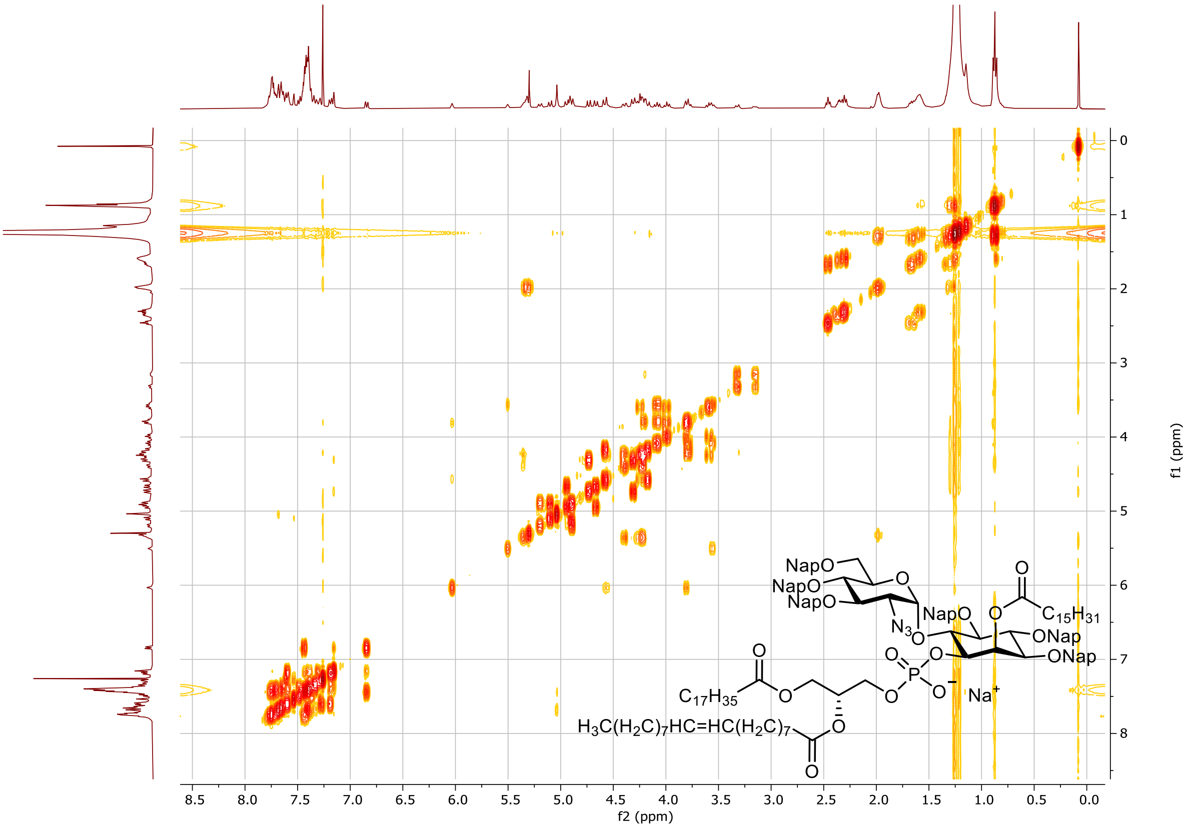

## HSQC-NMR spectrum of protected glycolipid 19

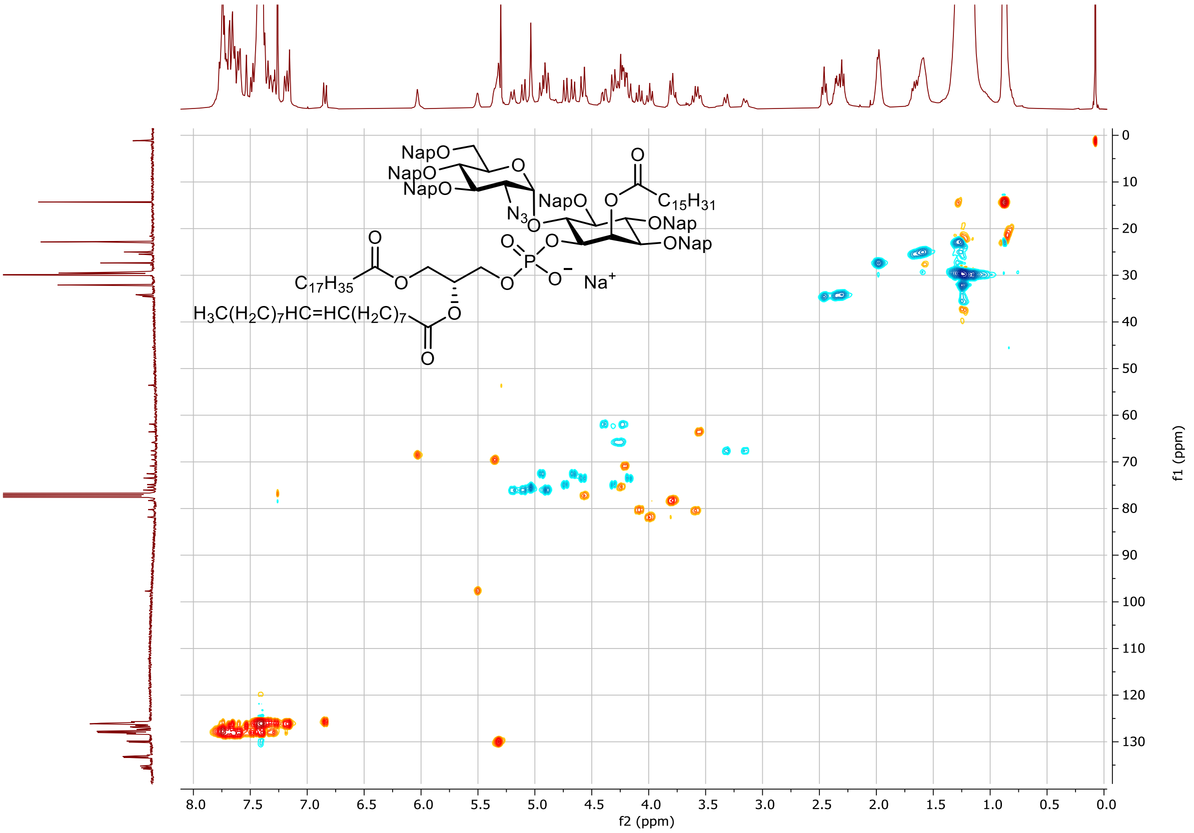


## $^{31}$P NMR spectrum of protected glycolipid 19

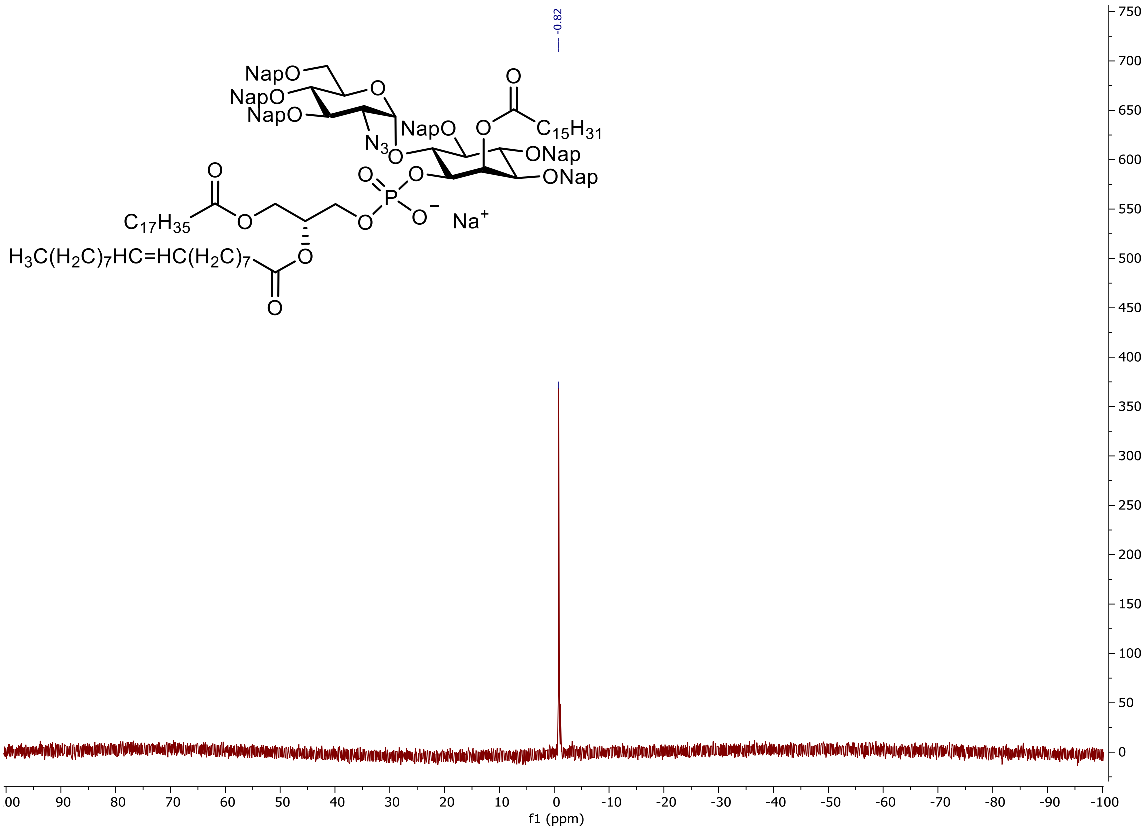

## $^{1}$H NMR spectrum of GPI-Fragment 4

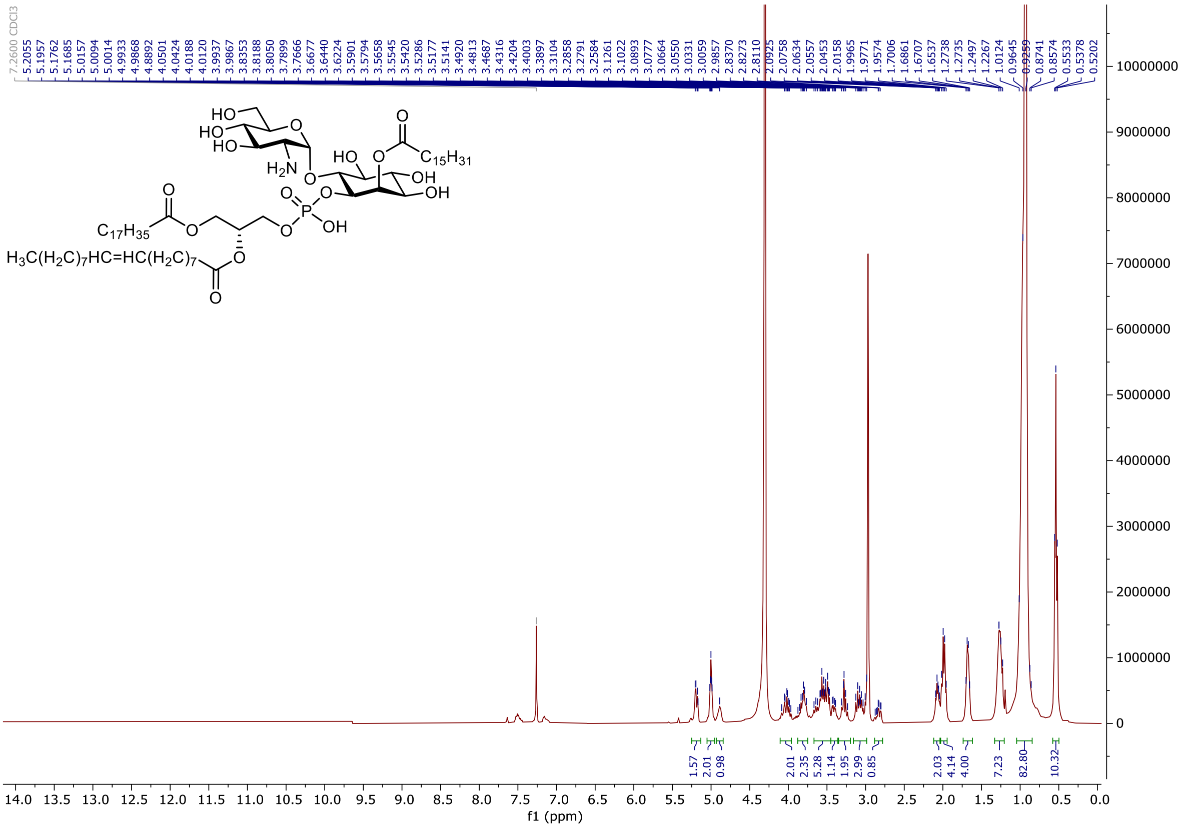

## $^{13}$C-NMR spectrum of GPI-Fragment 4

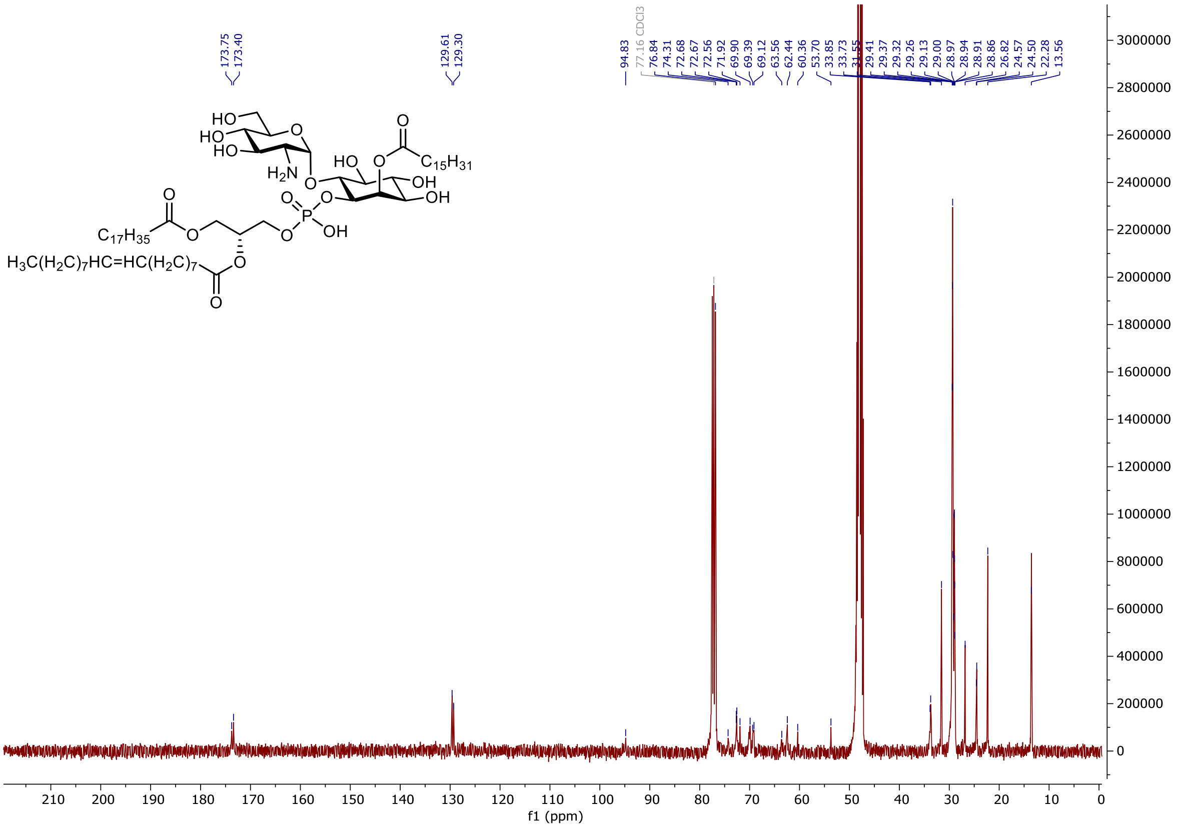

## COSY-NMR spectrum of GPI-Fragment 4

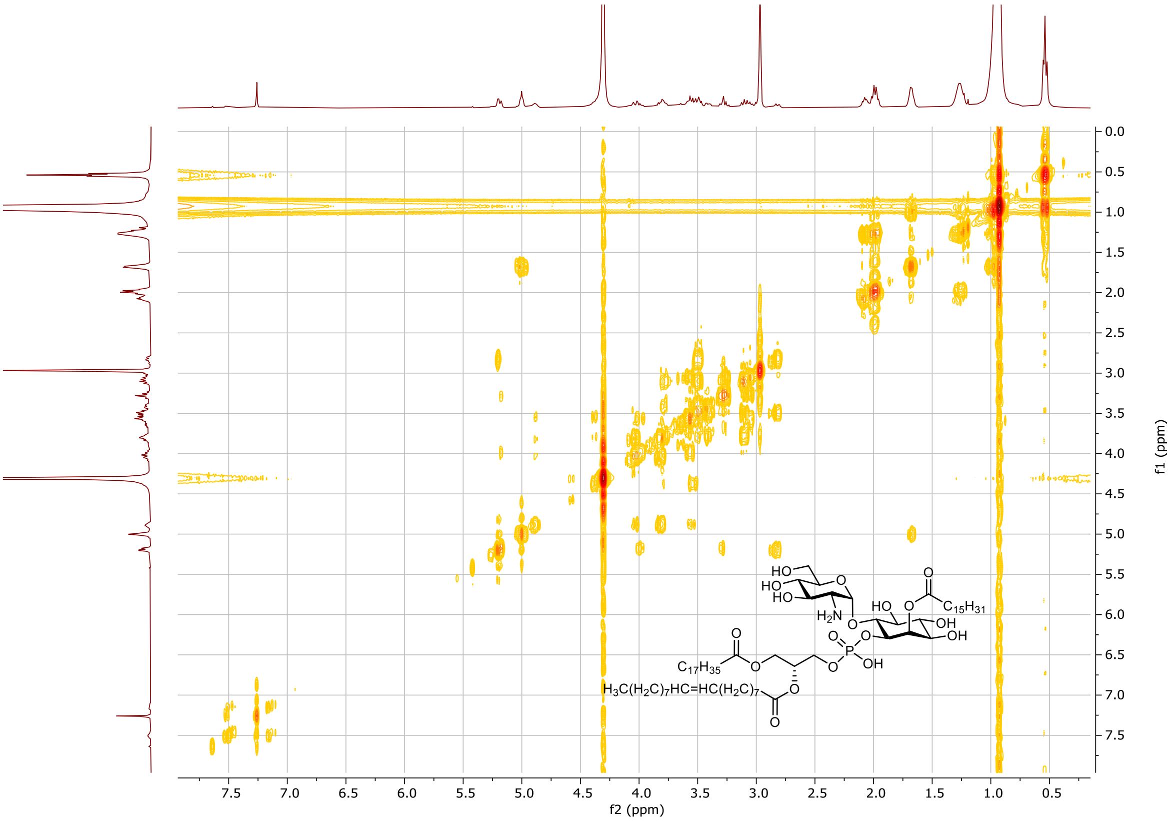

## HSQC-NMR spectrum of GPI-Fragment 4

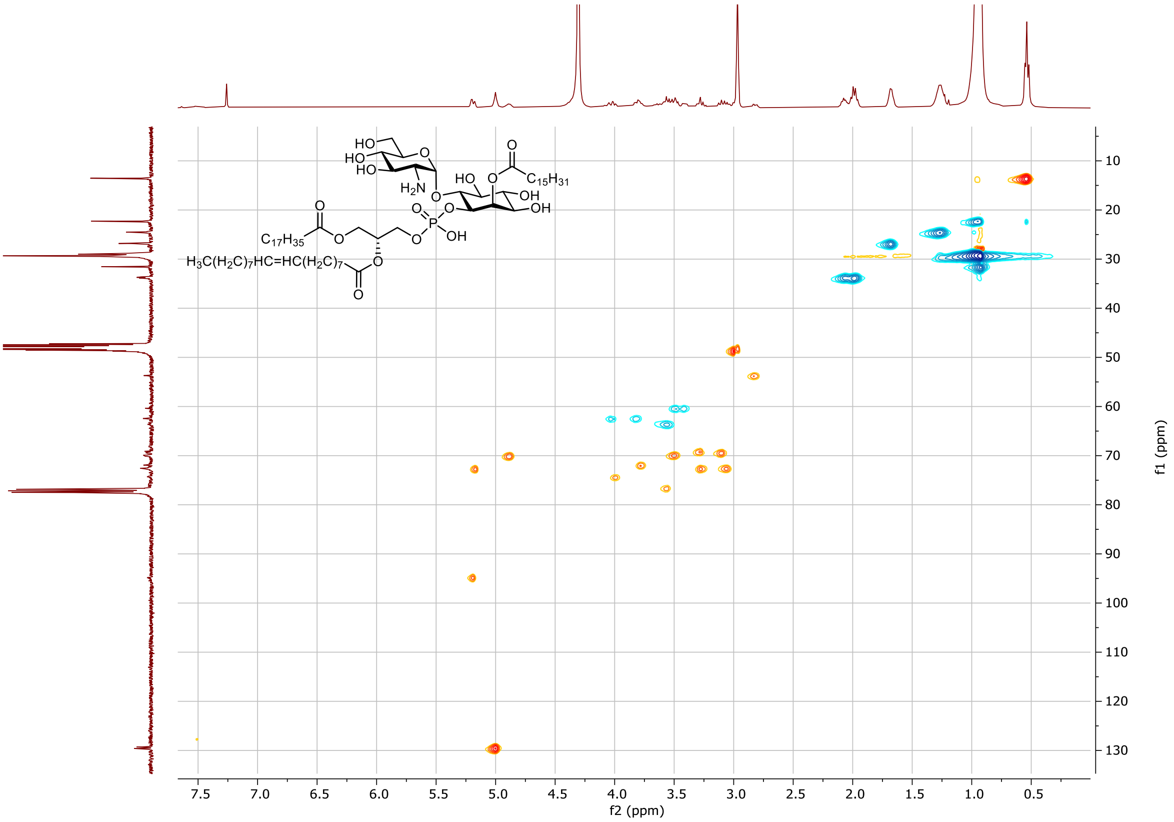

**$^{31}$P-NMR spectrum of GPI-Fragment 4**

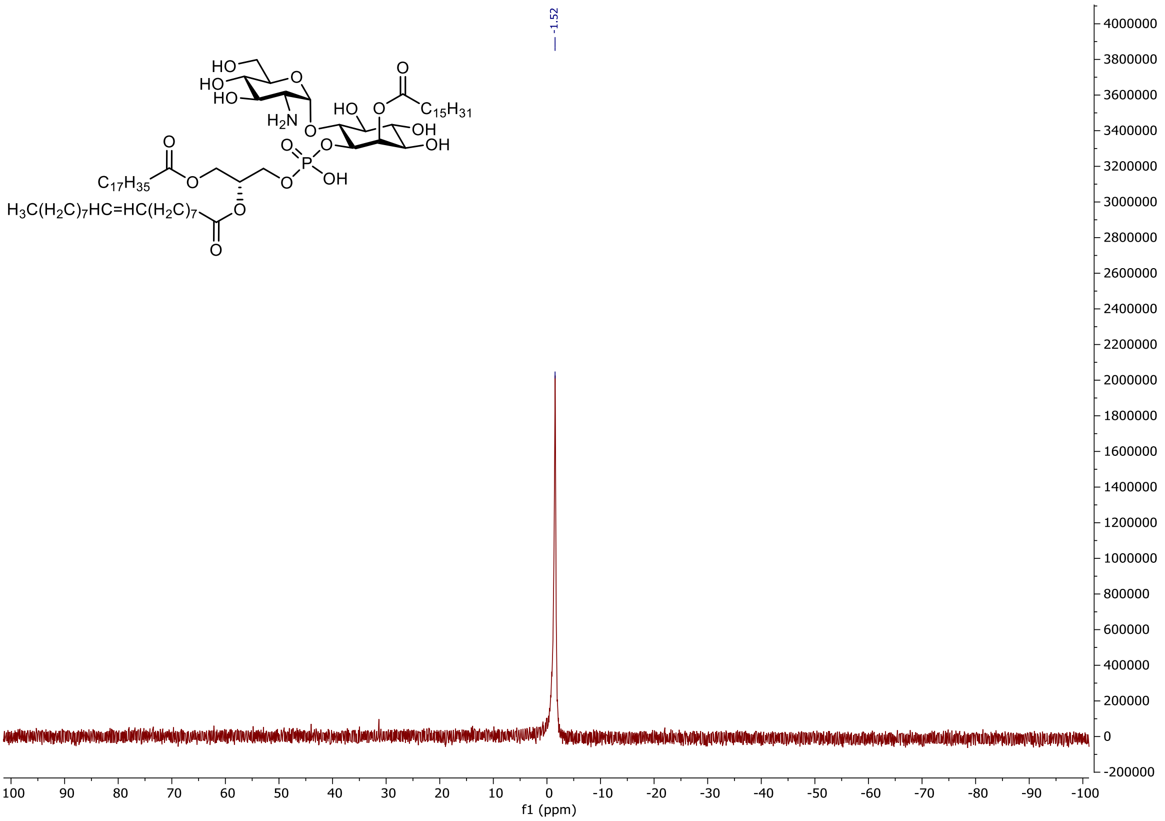

**$^{1}$H NMR spectrum of *pseudo*-disaccharide 20**

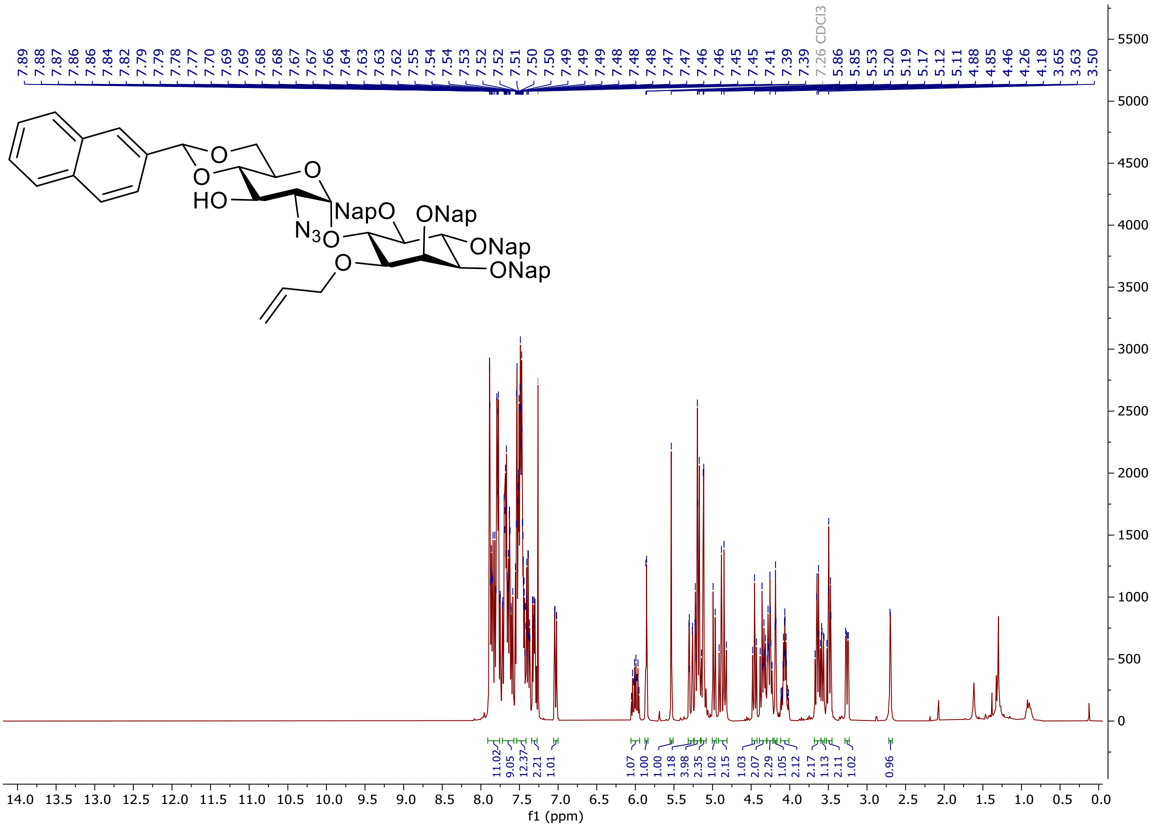

## $^{13}$C NMR spectrum of *pseudo*-disaccharide 20

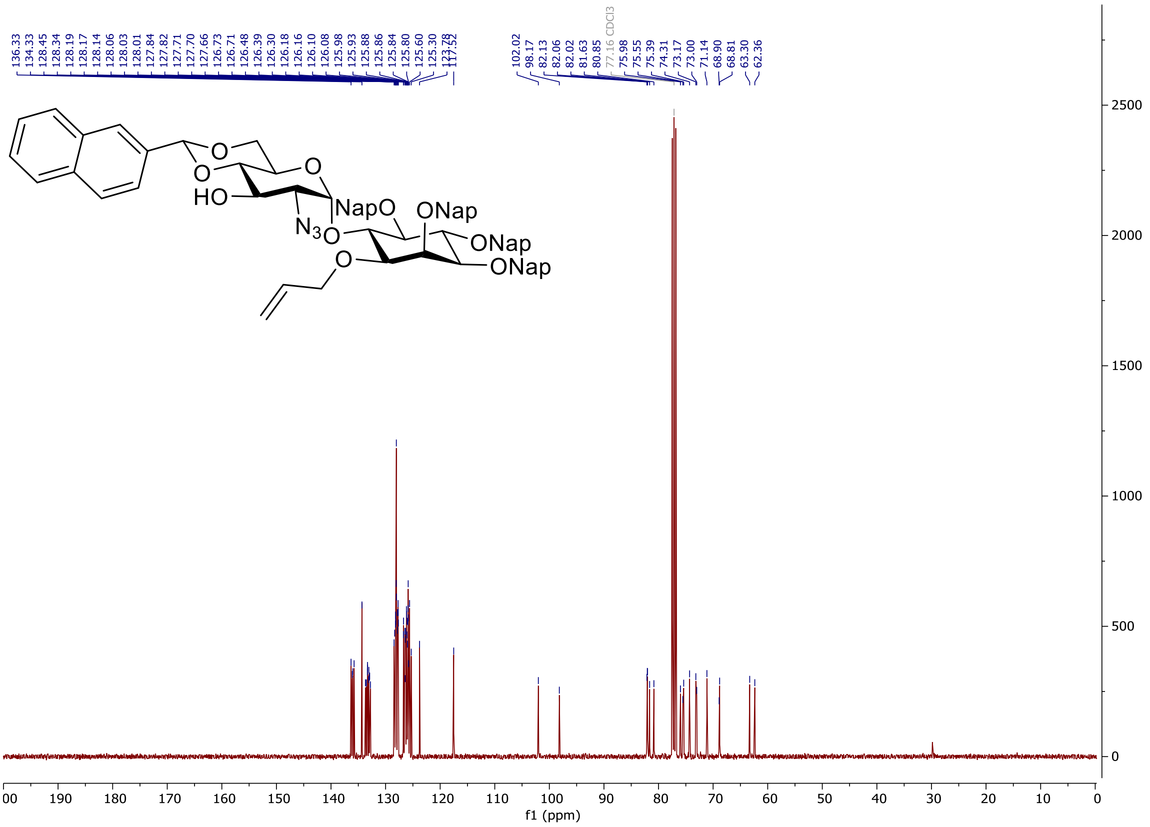


## COSY-NMR spectrum of *pseudo*-disaccharide 20

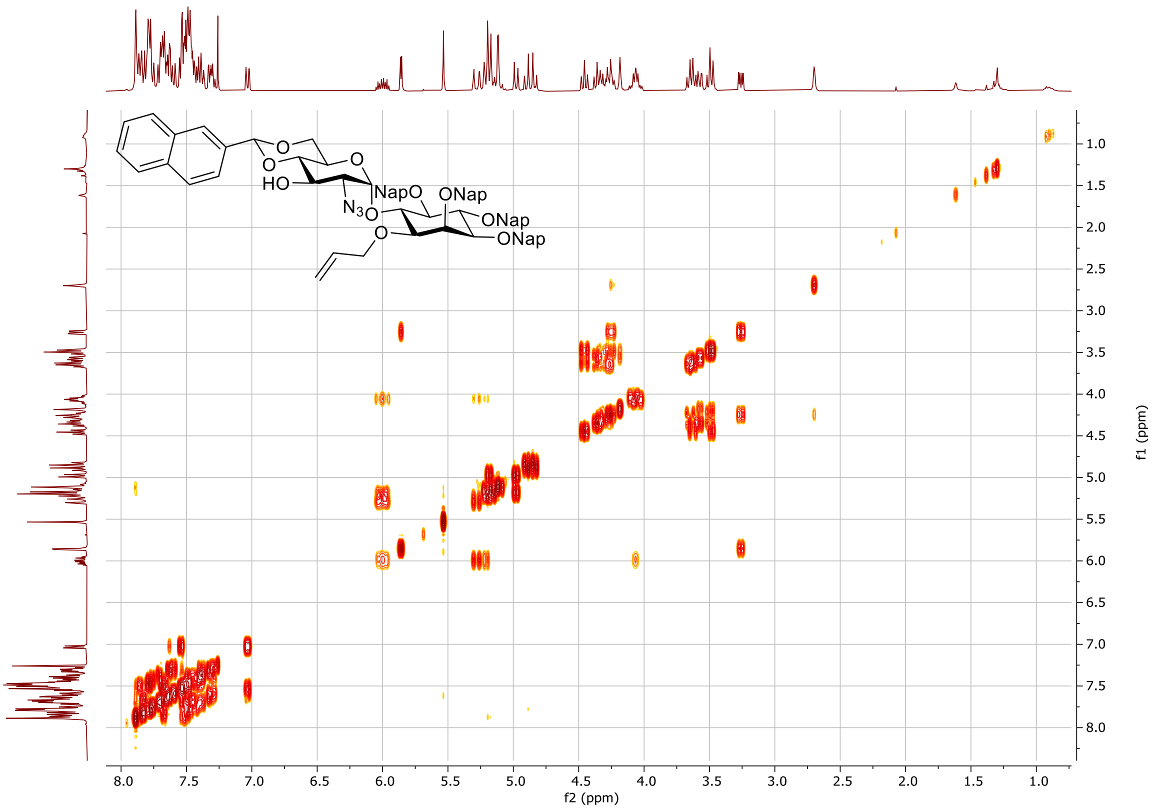

## HSQC-NMR spectrum of *pseudo*-disaccharide 20

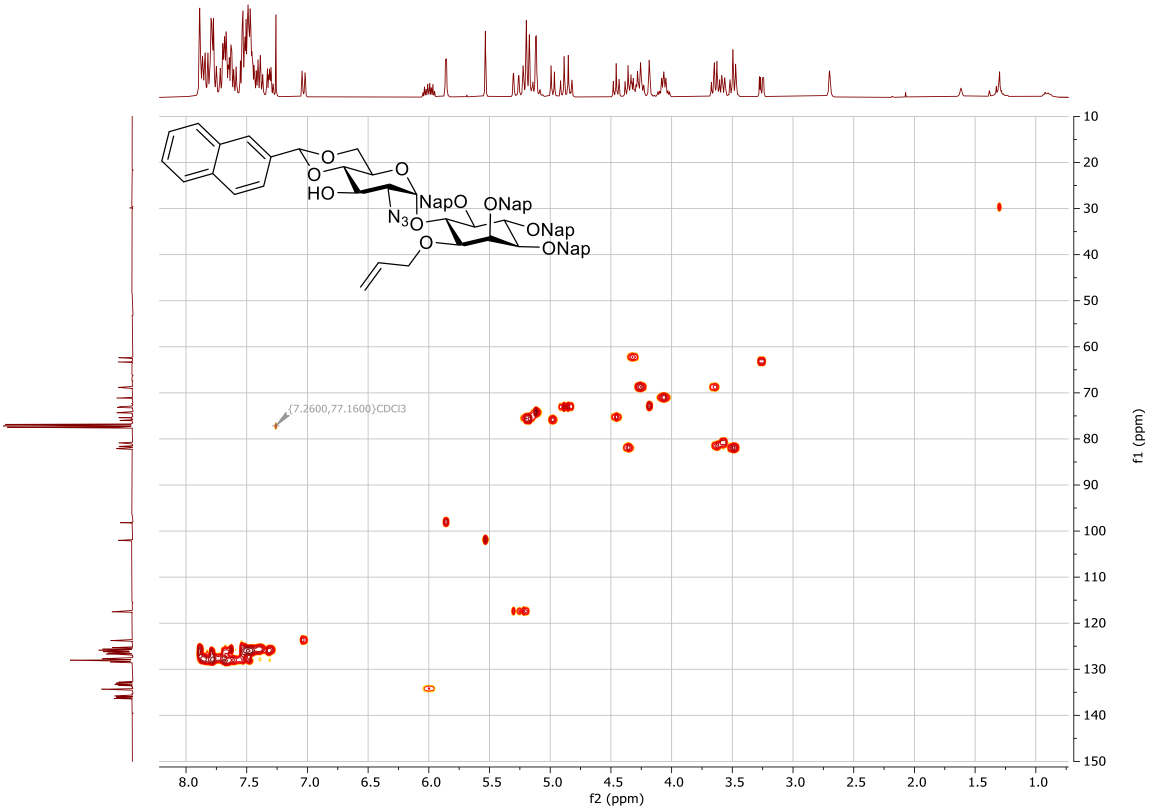

## $^1$H NMR spectrum of *pseudo*-disaccharide 21

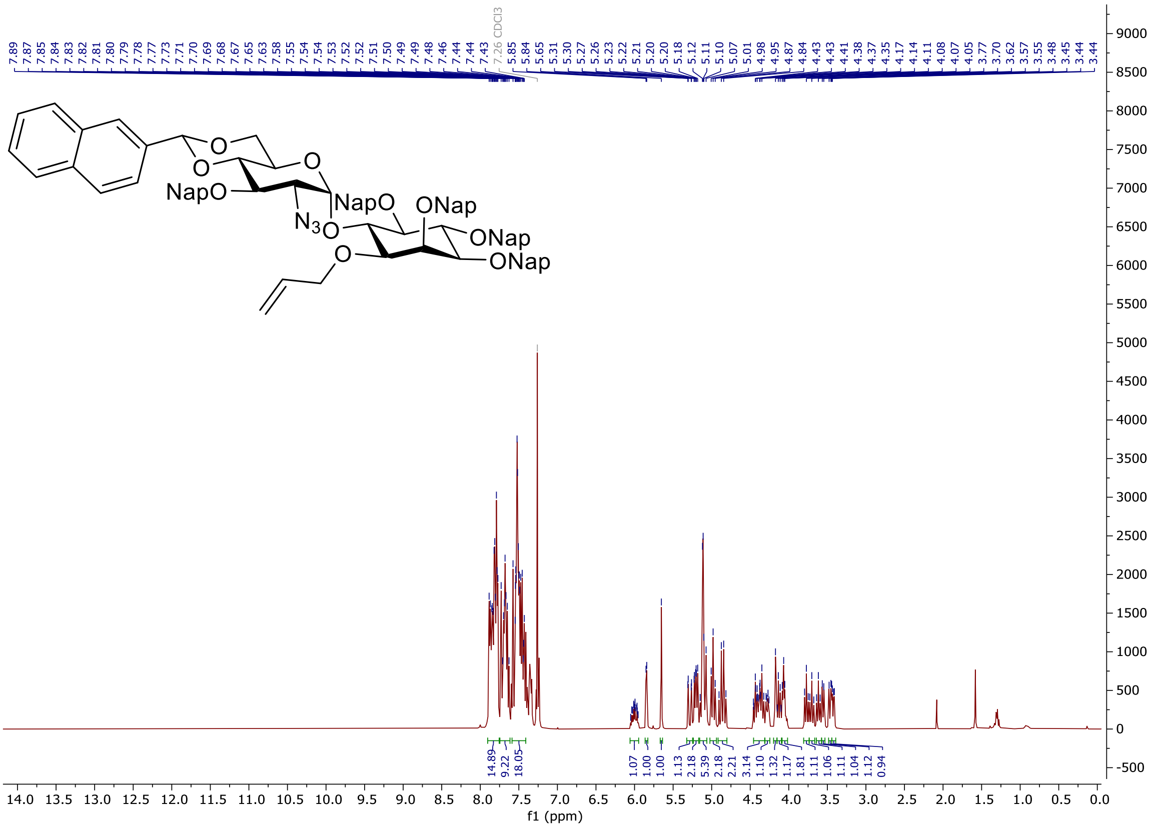

## $^{13}$C NMR spectrum of *pseudo*-disaccharide 21

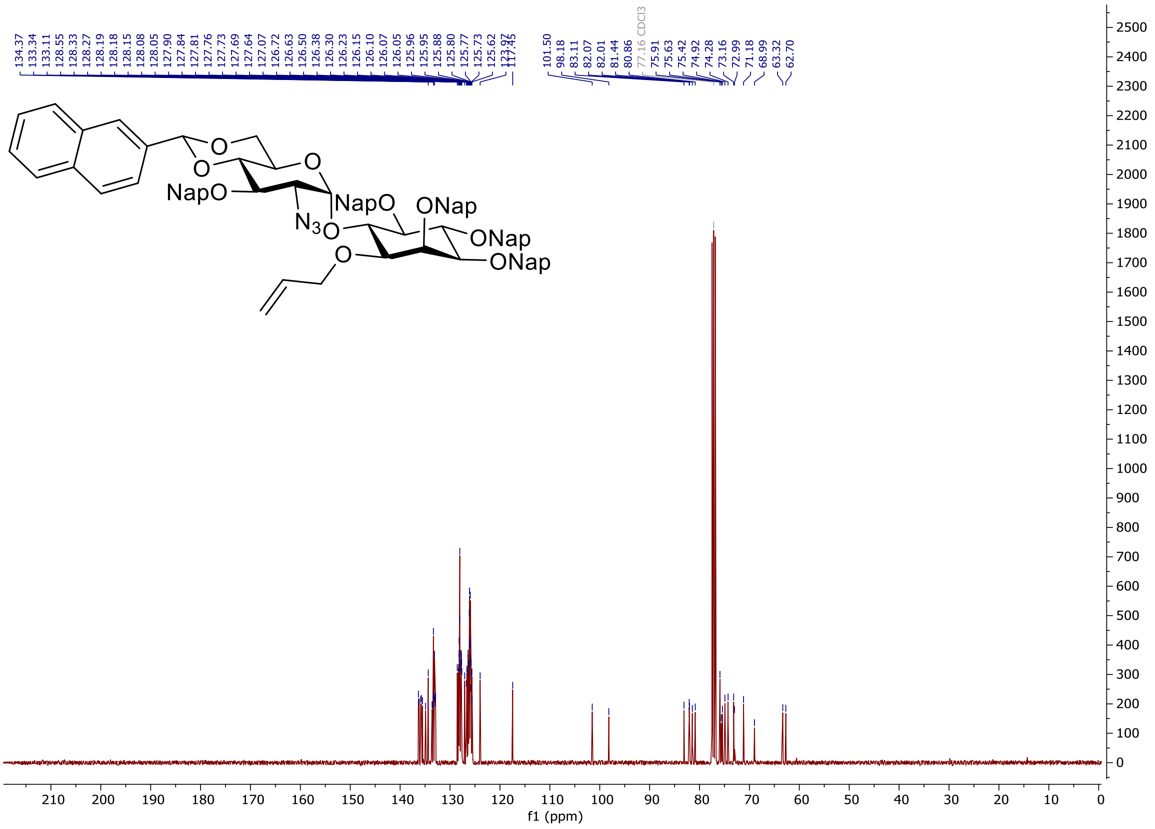

## COSY-NMR spectrum of *pseudo*-disaccharide 21

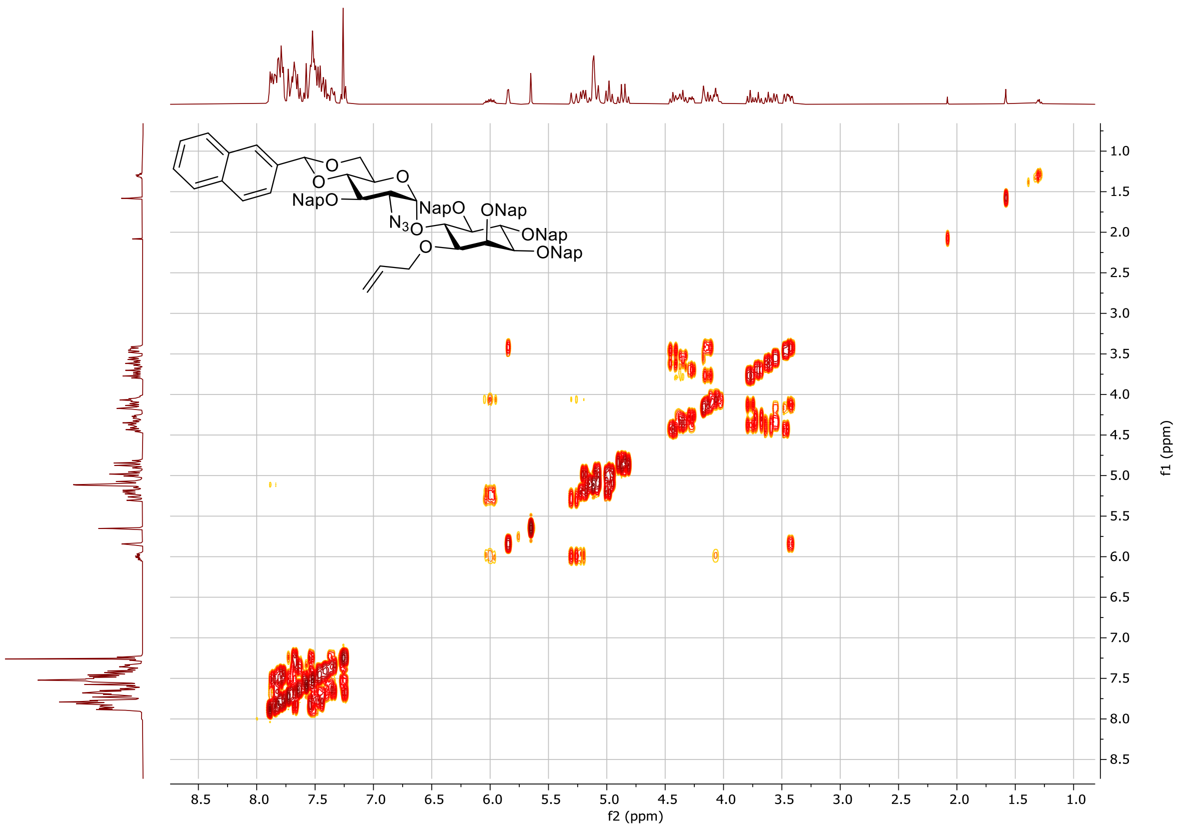

## HSQC-NMR spectrum of *pseudo*-disaccharide 21

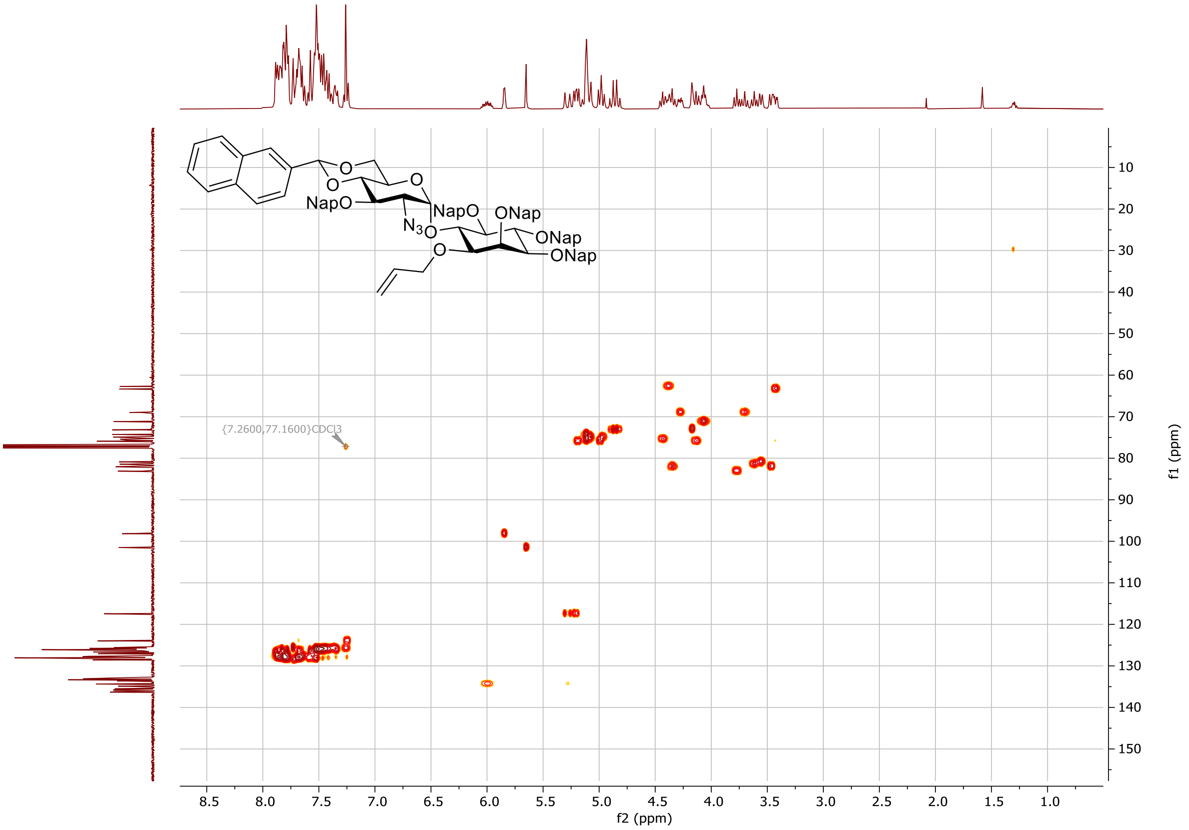

## $^{1}$H NMR spectrum of *pseudo*-disaccharide 22

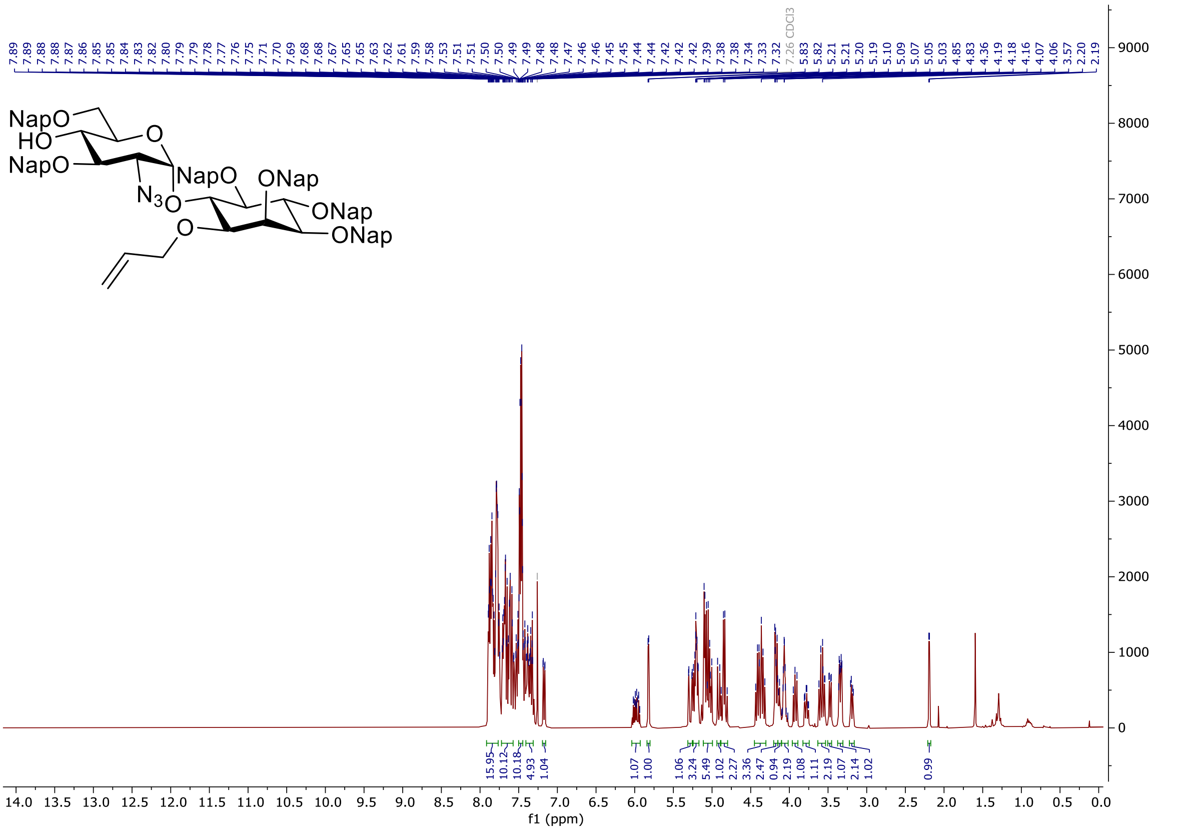

**$^{13}$C NMR spectrum of *pseudo*-disaccharide 22**

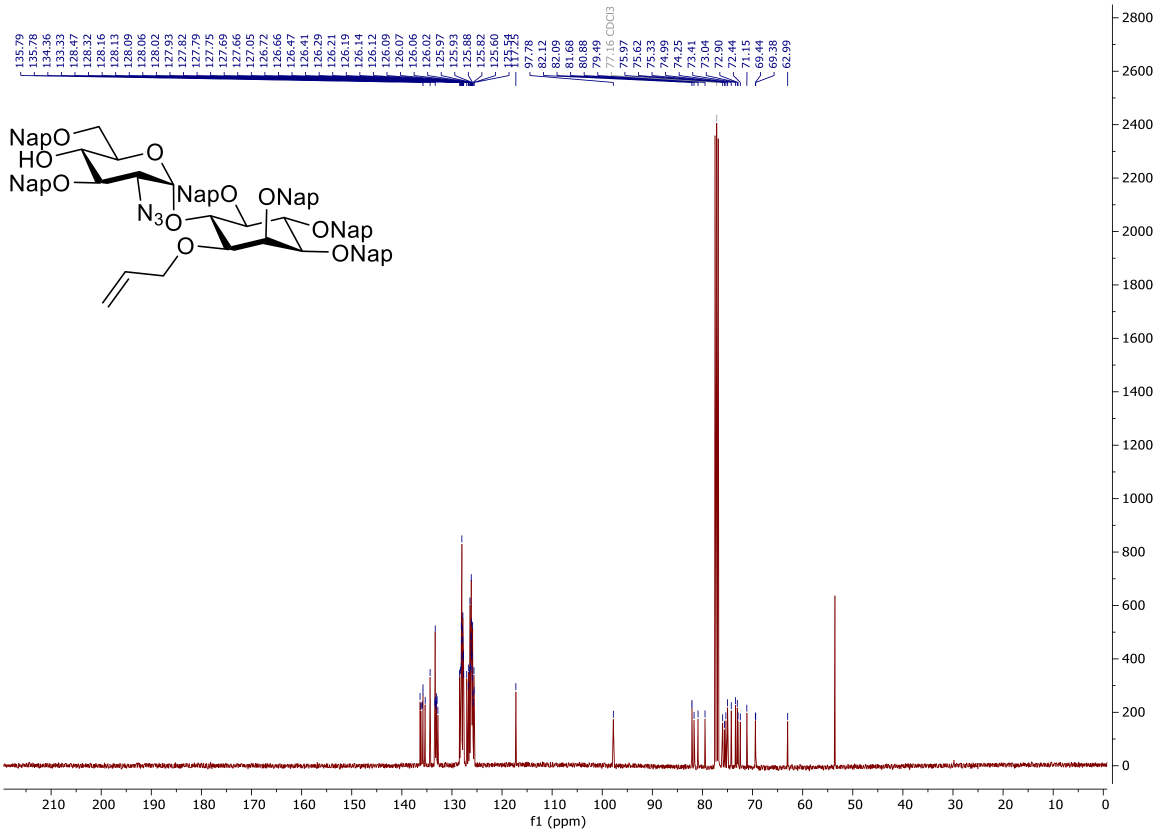

**COSY-NMR spectrum of *pseudo*-disaccharide 22**

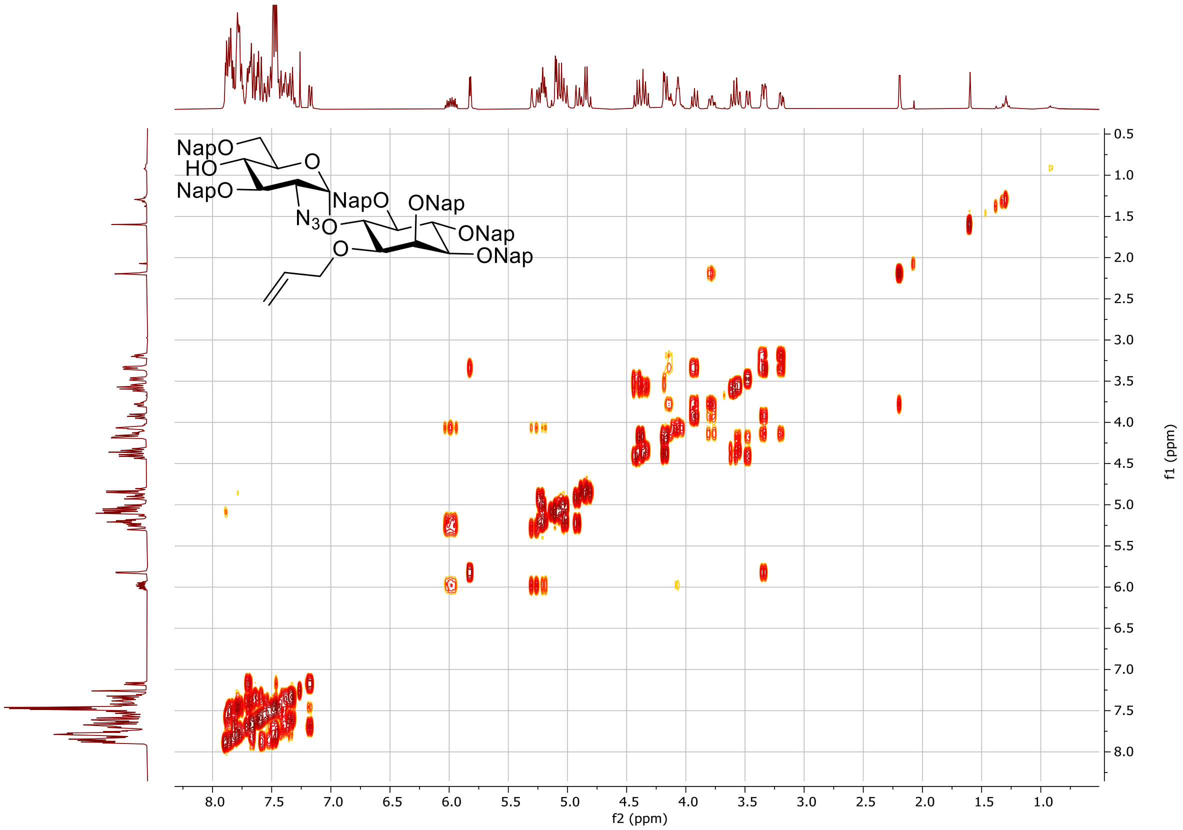

**HSQC-NMR spectrum of *pseudo*-disaccharide 22**

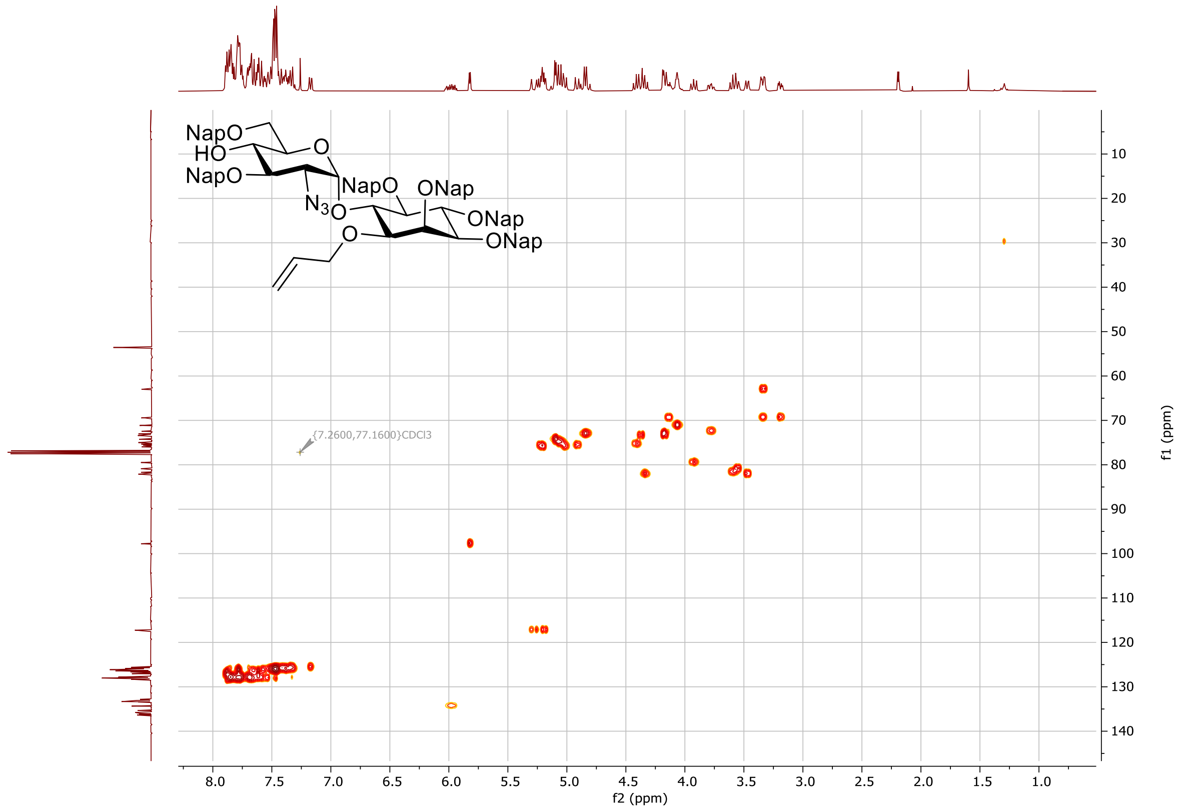

**$^{1}$H NMR spectrum of *pseudo*-trisaccharide 8**

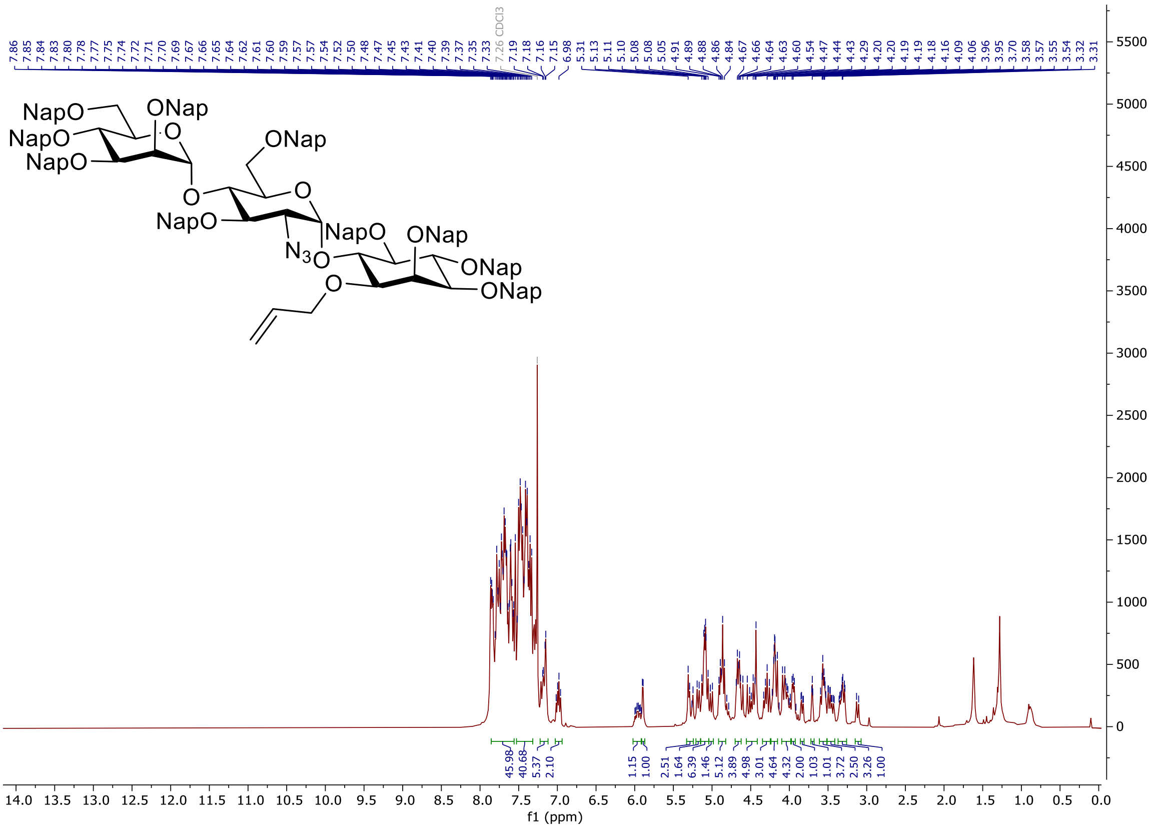

## $^{13}$C NMR spectrum of *pseudo*-trisaccharide 8

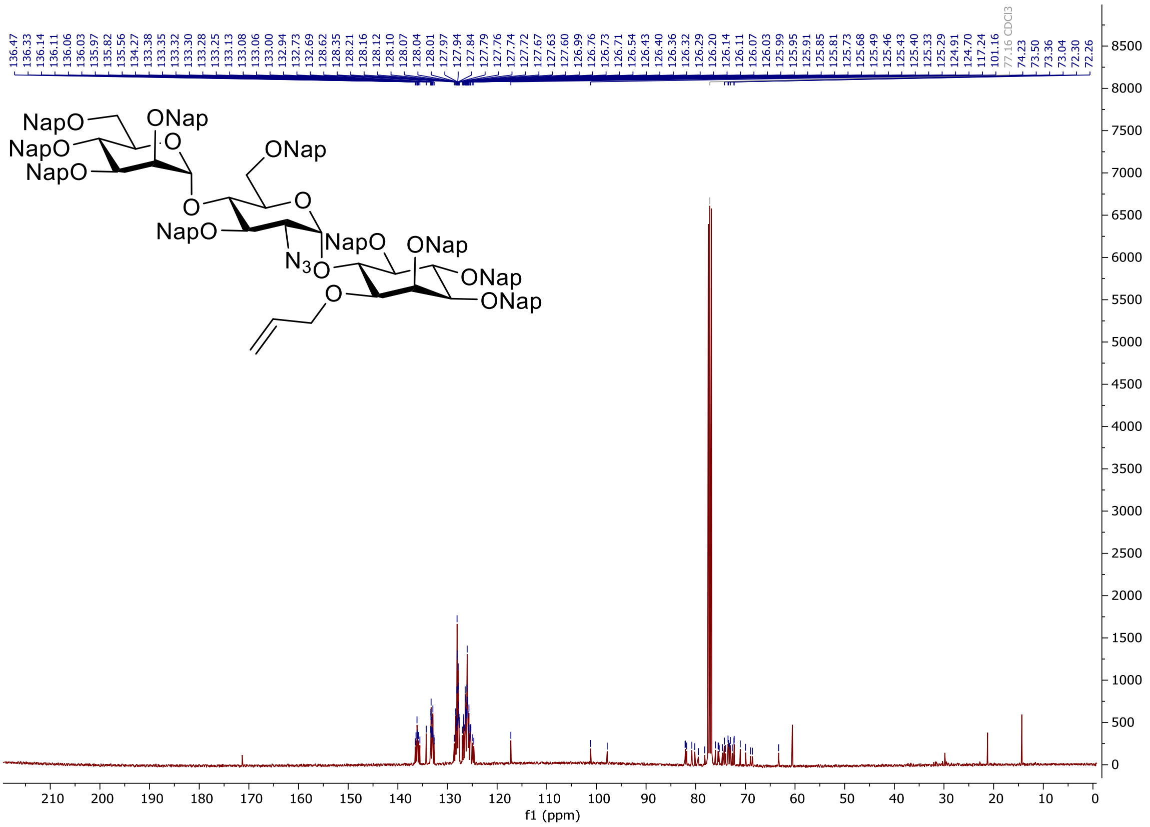


## COSY-NMR spectrum of *pseudo*-trisaccharide 8

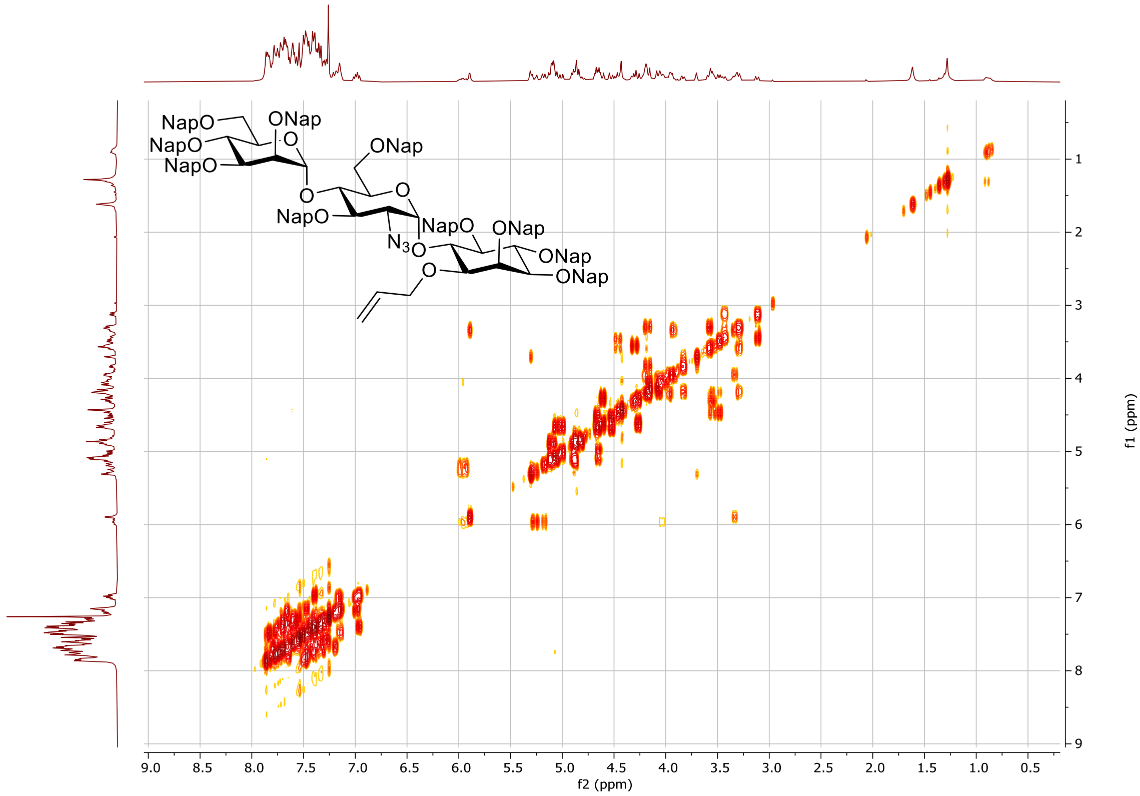

## HSQC-NMR spectrum of *pseudo*-trisaccharide 8

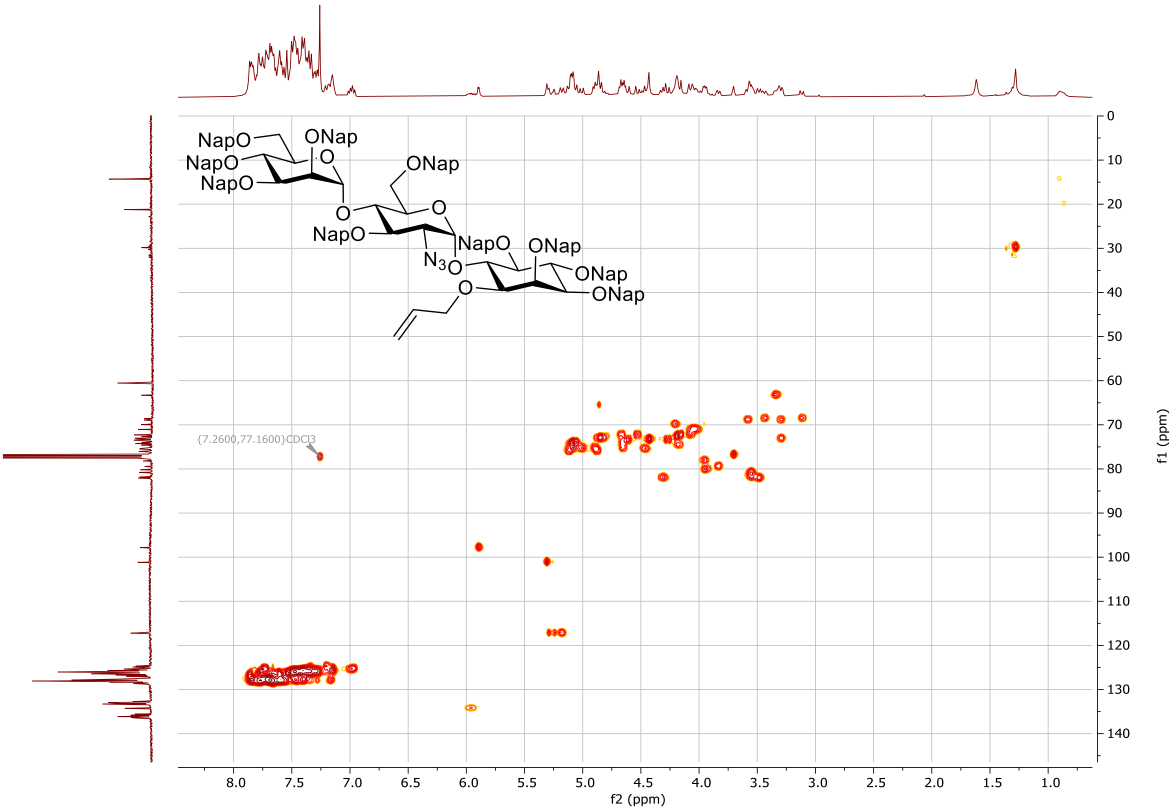

## $^{1}$H NMR spectrum of *pseudo*-trisaccharide 24

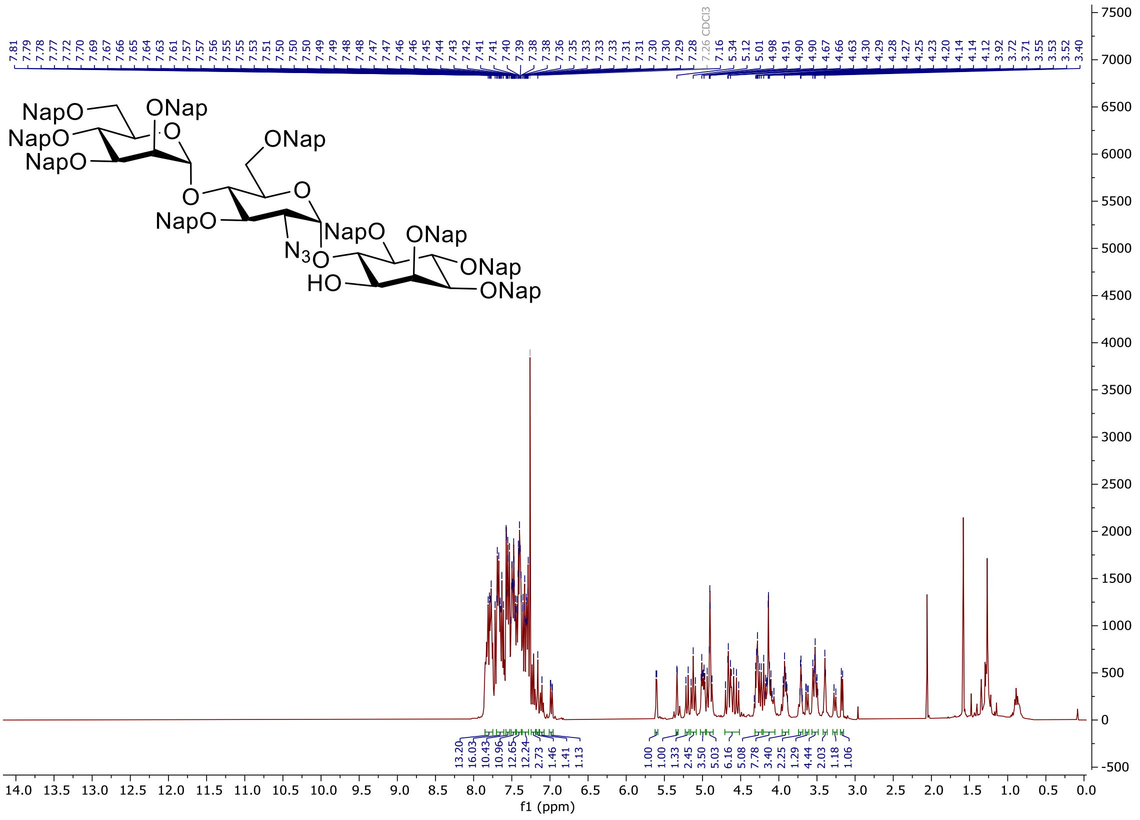

## $^{13}$C NMR spectrum of *pseudo*-trisaccharide 24

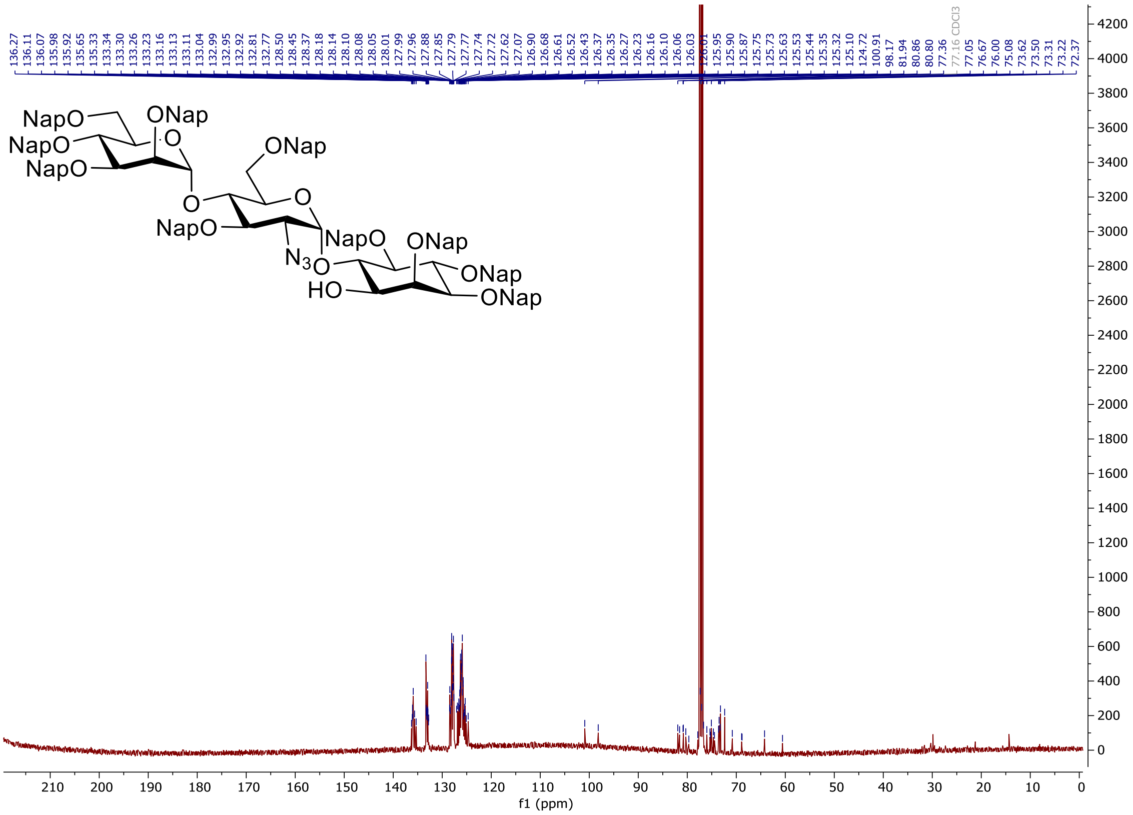


## COSY-NMR spectrum of *pseudo*-trisaccharide 24

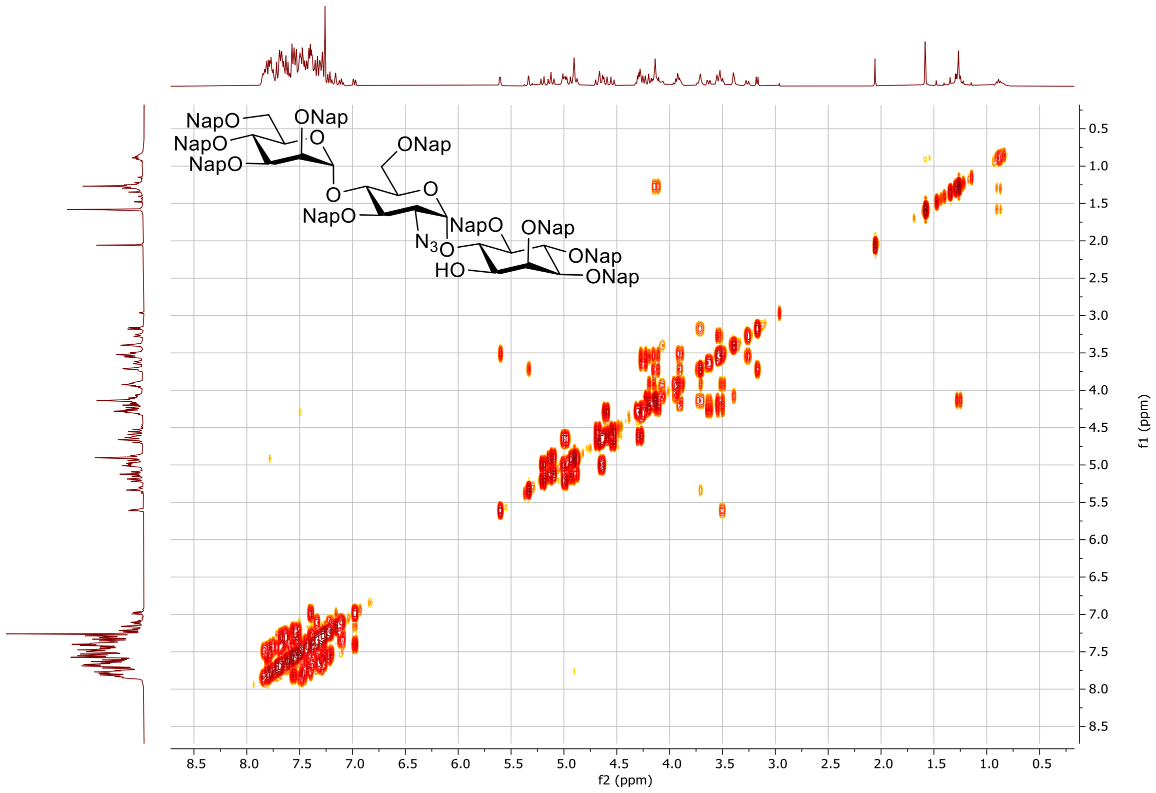

## HSQC-NMR spectrum of *pseudo*-trisaccharide 24

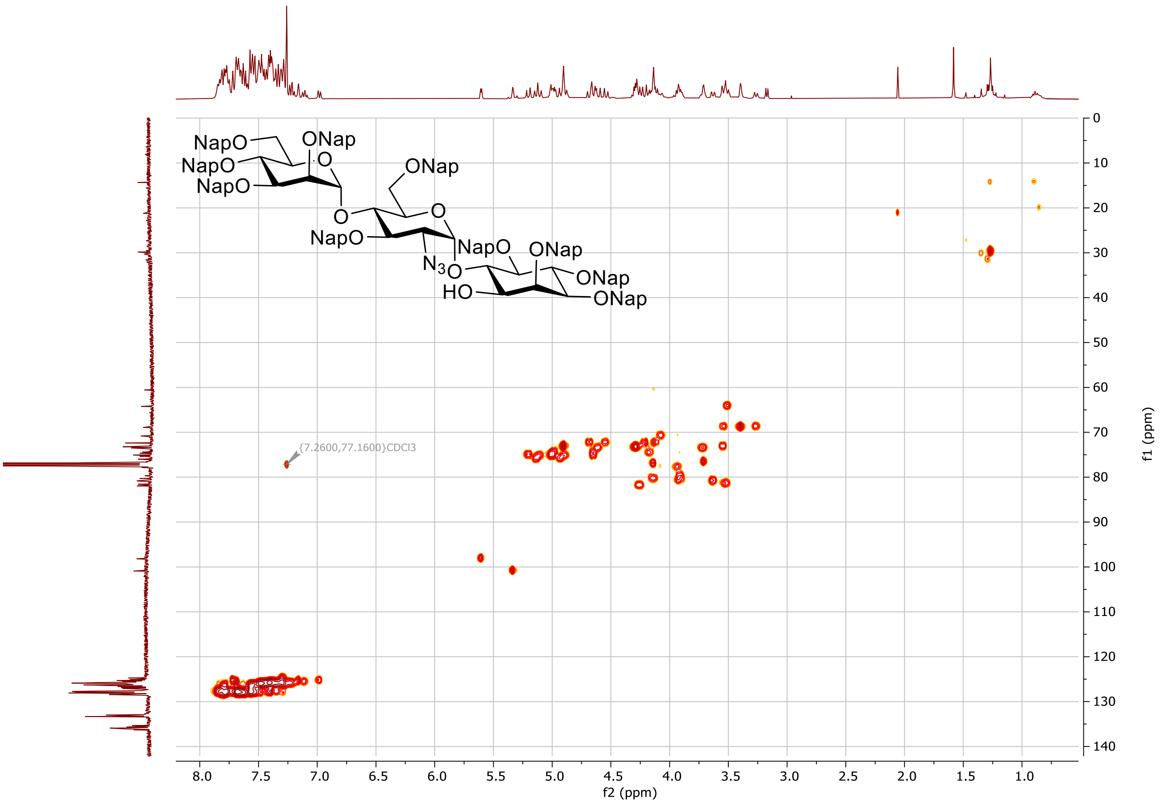

## $^{1}$H NMR spectrum of protected glycolipid 25

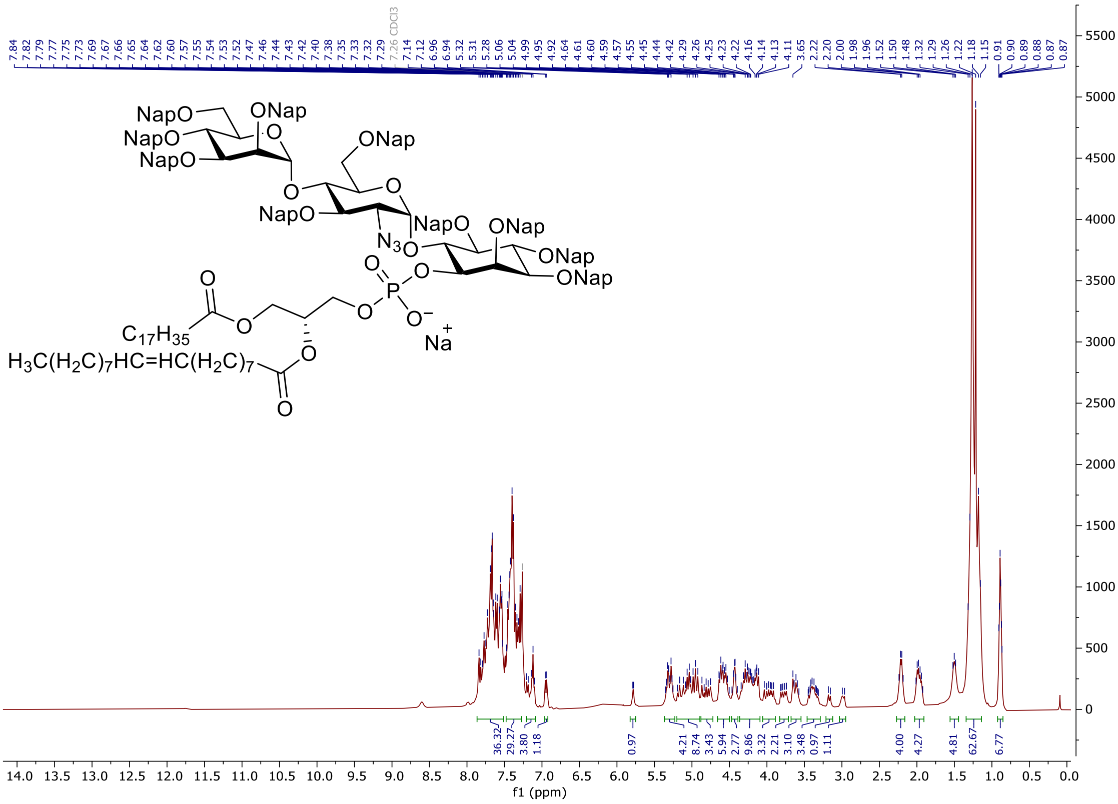

## $^{13}$C NMR spectrum of protected glycolipid 25

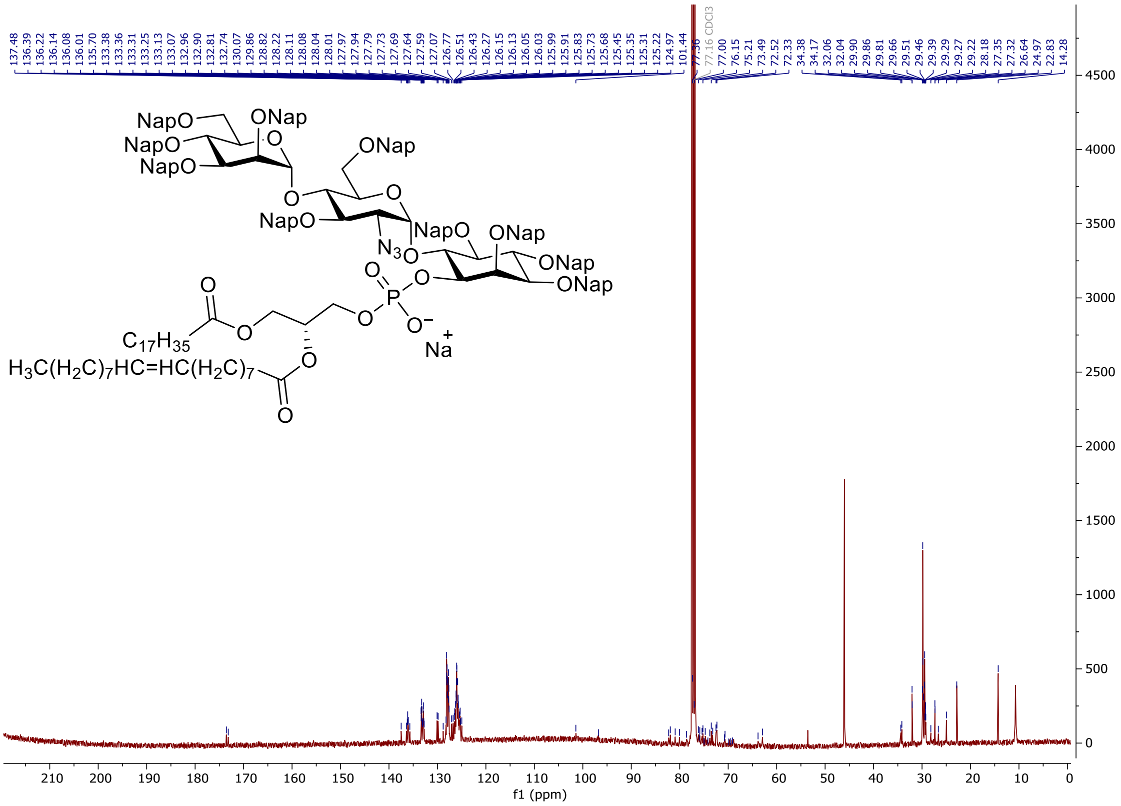

## COSY-NMR spectrum of protected glycolipid 25

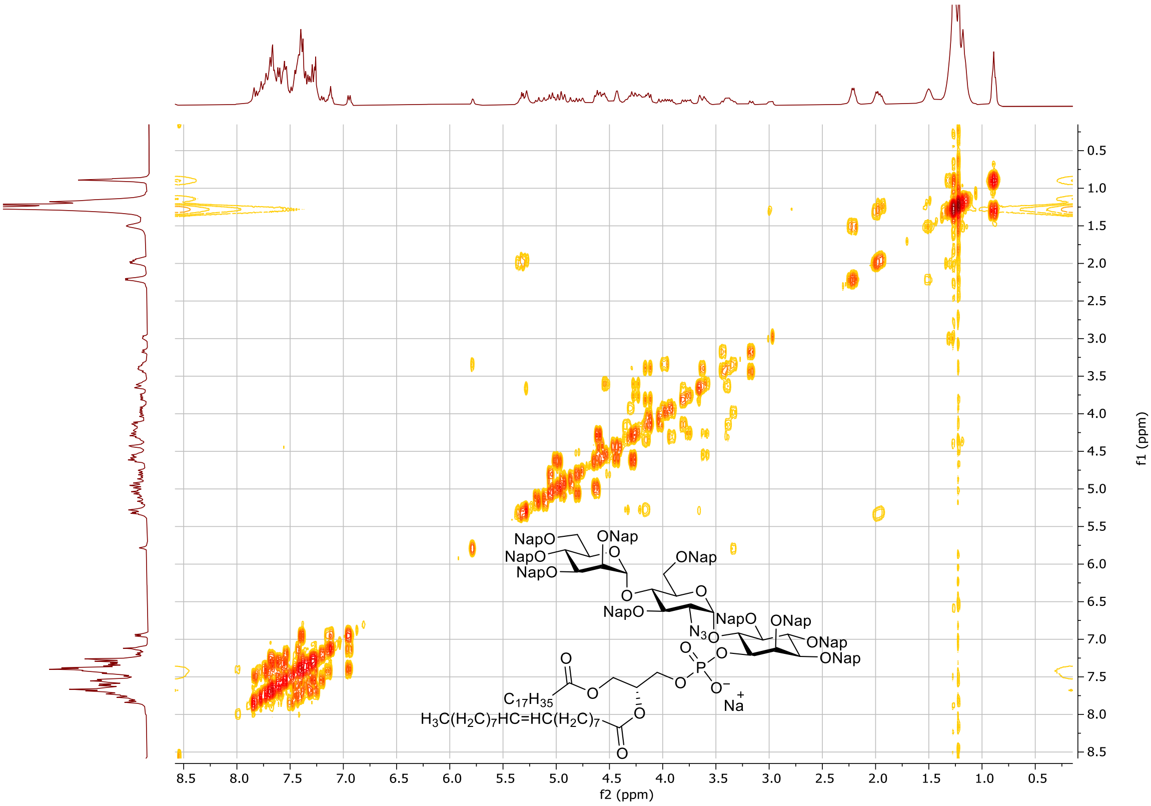

## HSQC-NMR spectrum of protected glycolipid 25

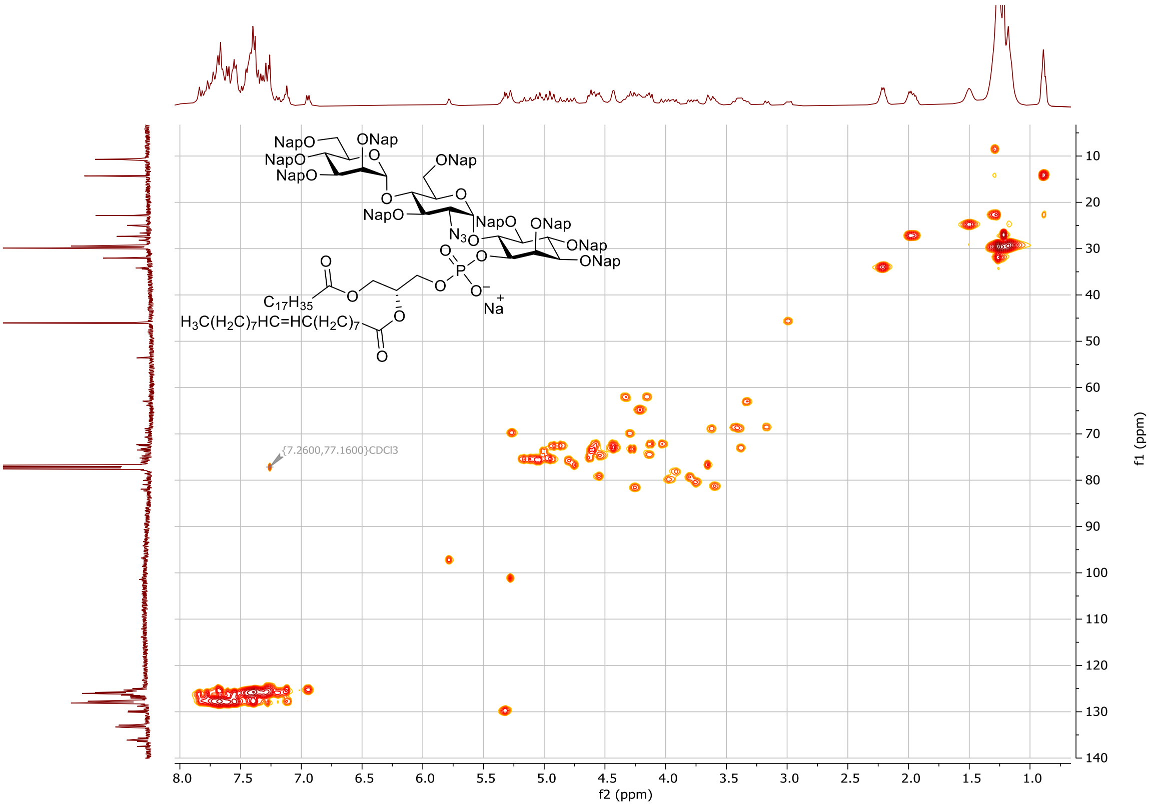


## $^{31}$P NMR spectrum of protected glycolipid 25

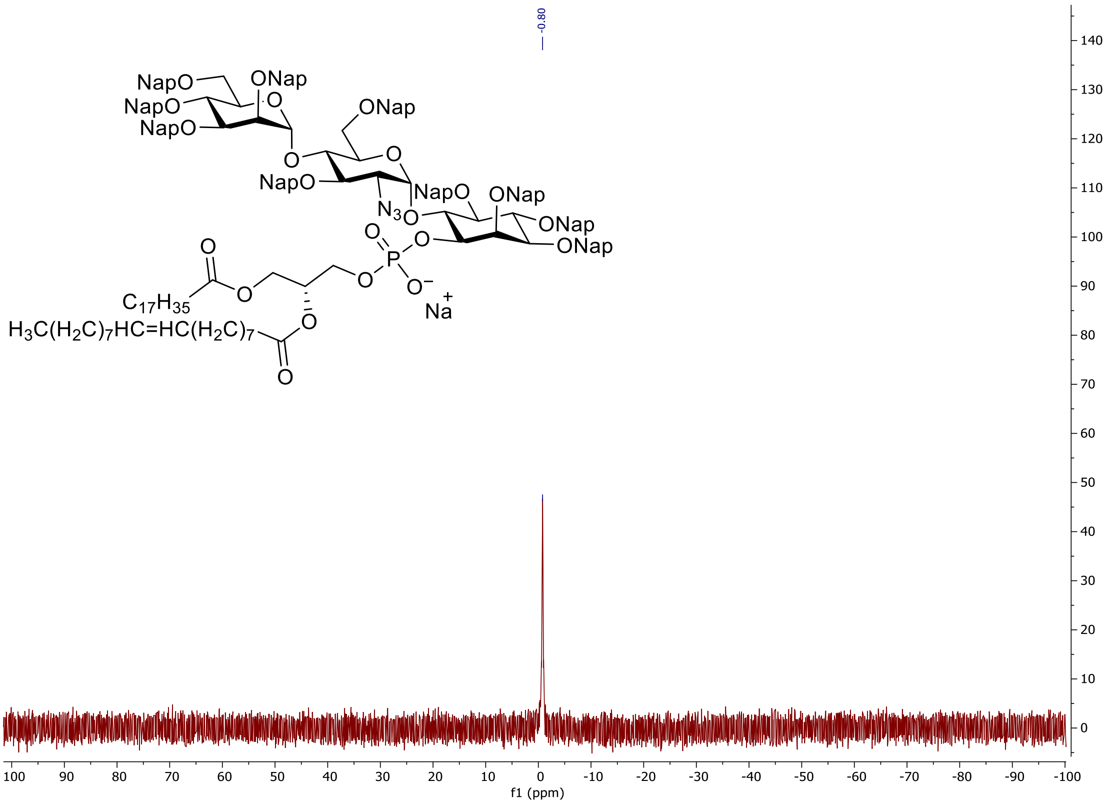

## $^{1}$H NMR spectrum of GPI-Fragment 5

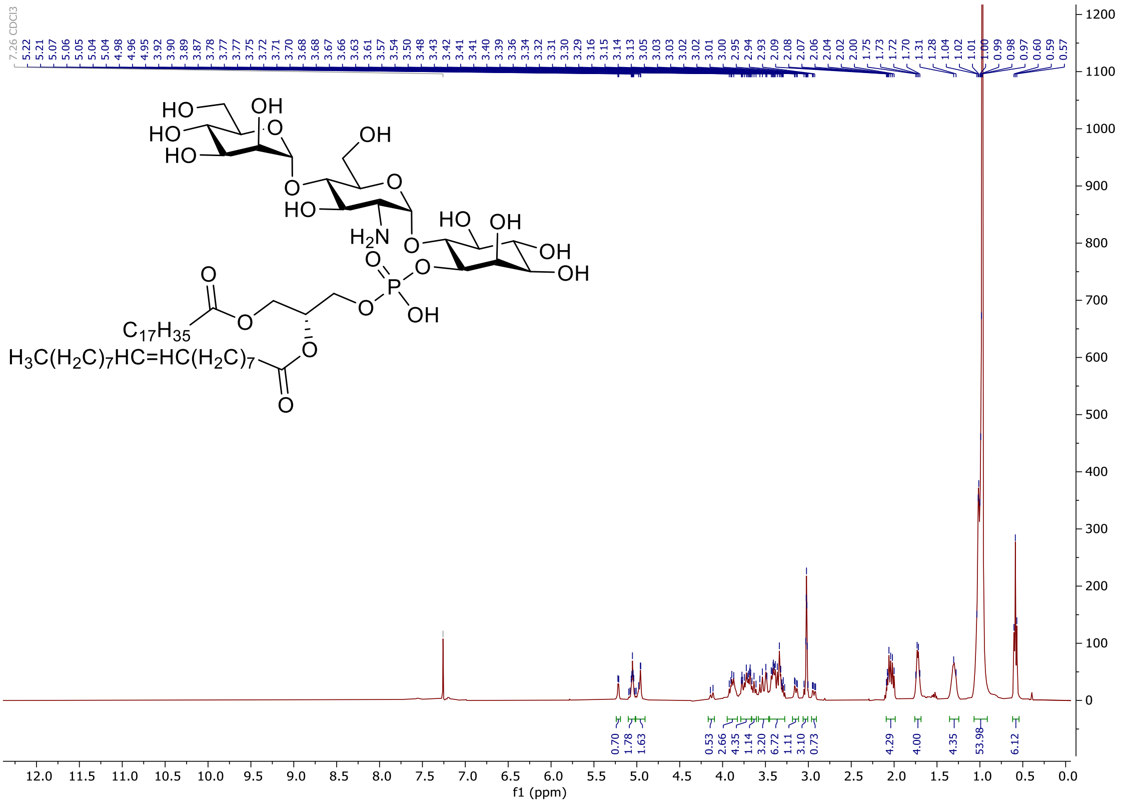

## $^{13}$C NMR spectrum of GPI-Fragment 5

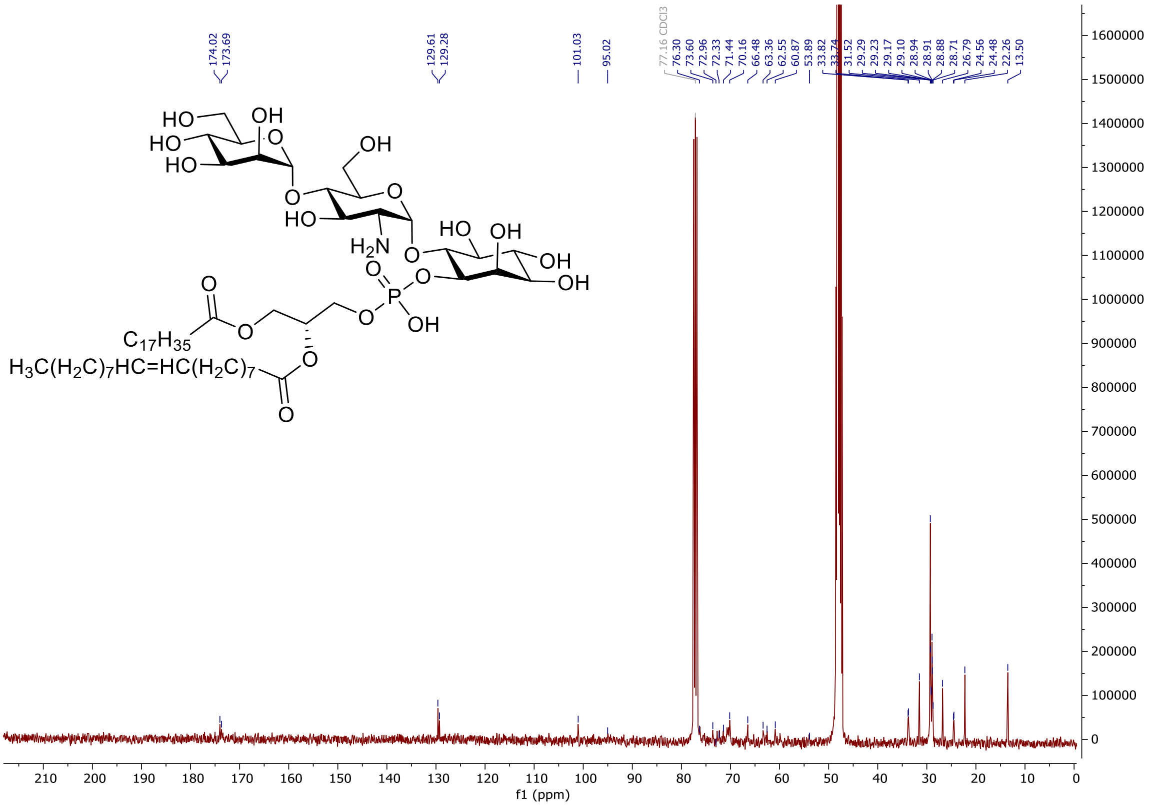

## COSY-NMR spectrum of GPI-Fragment 5

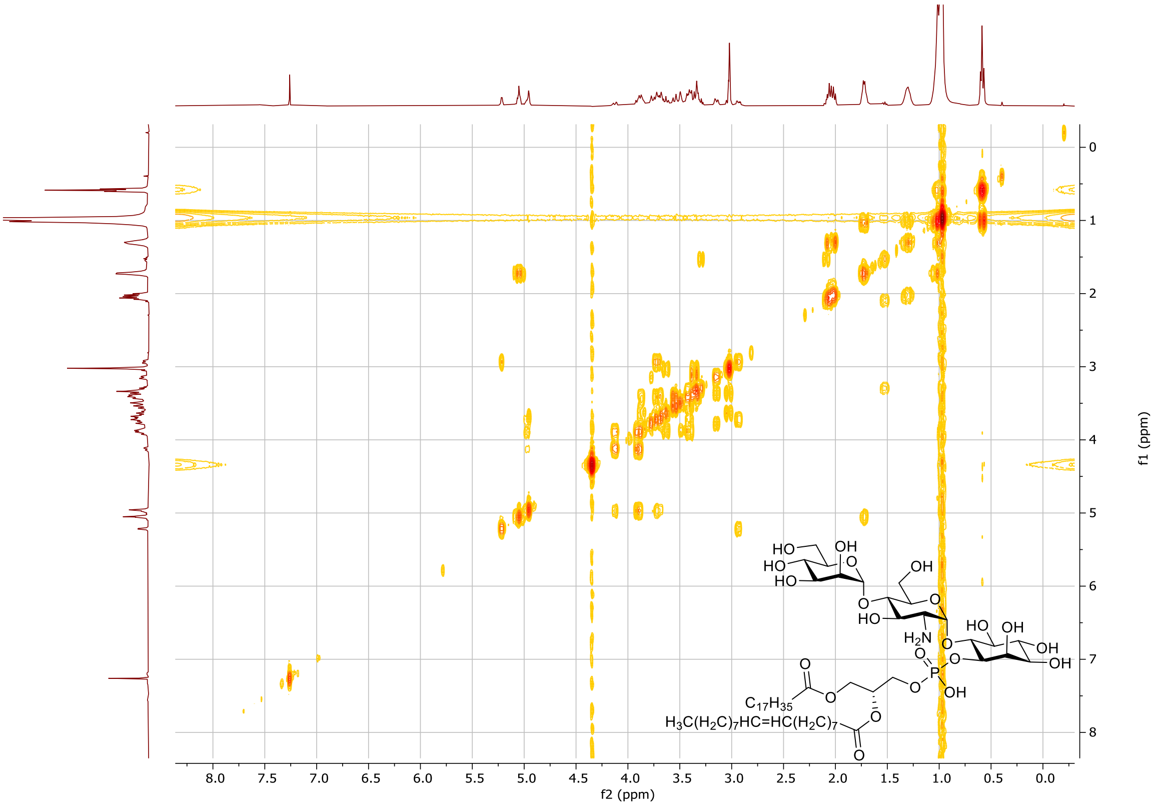


## HSQC-NMR spectrum of GPI-Fragment 5

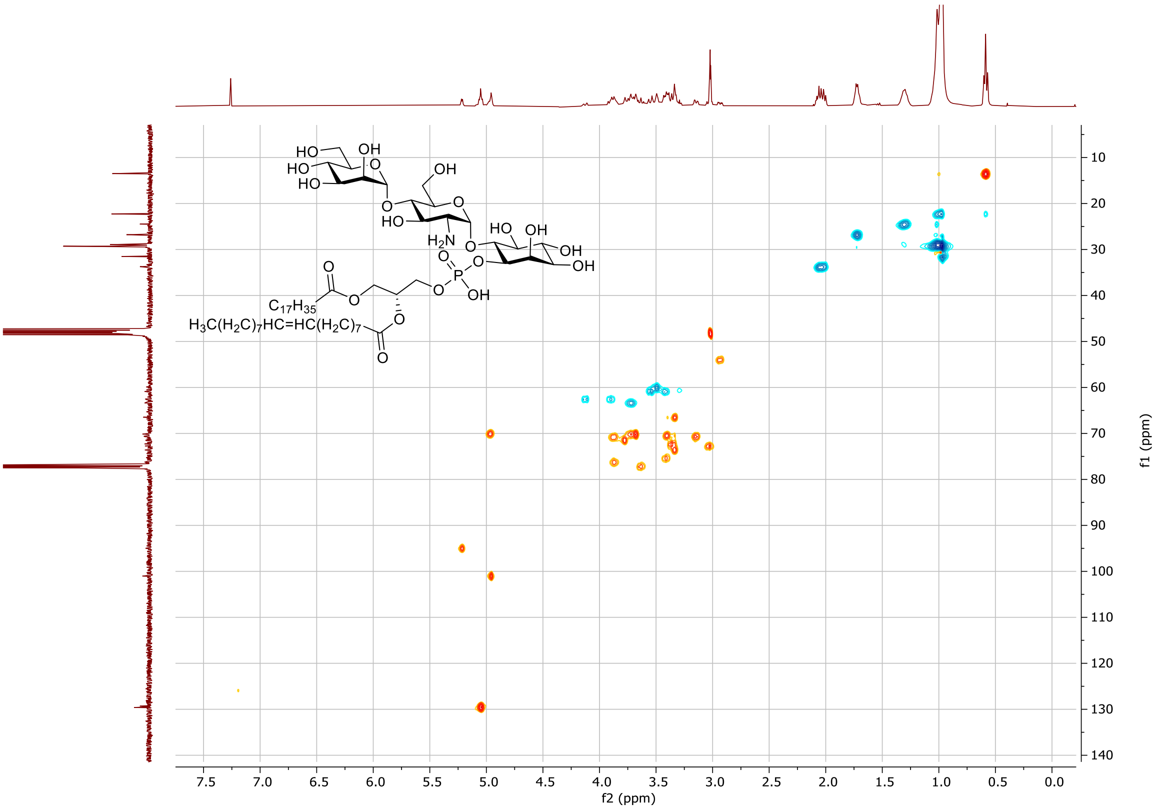

**$^{31}$P-NMR spectrum of GPI-Fragment 5**

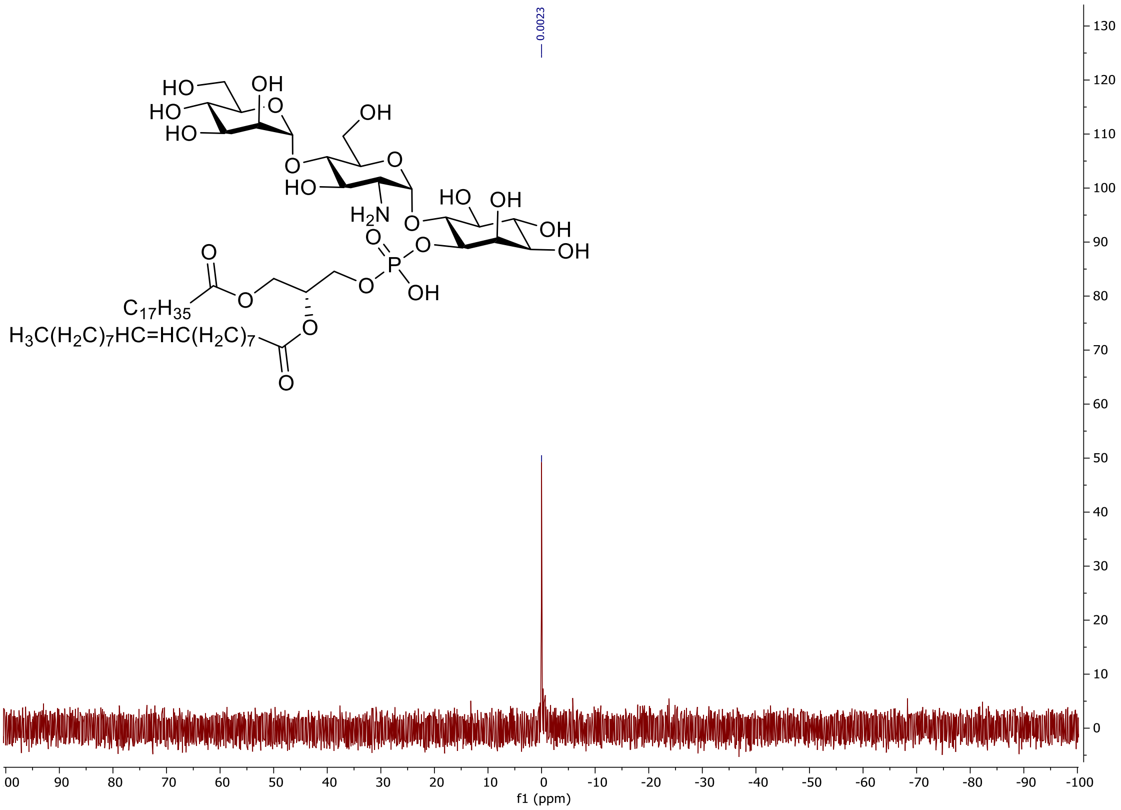

Supplement: Supplementary file 1 — cb1c00465_si_001.pdf [file cb1c00465_si_001.pdf]
